# Supplementary material for: A network-based modeling framework reveals the core signal transduction network underlying high carbon dioxide-induced stomatal closure in guard cells
Source: PLoS Biol. 2024 May 1;22(5):e3002592. doi: 10.1371/journal.pbio.3002592 (PMC11090369; doi:10.1371/journal.pbio.3002592)
Supplement: S1 Table — (DOCX) [file pbio.3002592.s001.docx]

**S1 Table. Summary of evidence from our literature review for CO_2_ network and model construction.**

**Table S1A. Summary of interactions and regulatory relationships collected for CO_2_ network model, from more than 160 articles in the literature.**

In this file we comprehensively list all interactions and regulations we gathered from our review of the guard cell signaling literature, and evaluate which of them should be used to construct the signaling network corresponding to high CO_2_ induced stomatal closure.

**Description of columns**

The first column indicates the regulator node, the second column indicates the target node or target process, the third column indicates the effect of the interaction, i.e. whether the relationship is positive (“promotes”) or negative (“inhibits”), the fourth column indicates the interaction type (direct or not). The fifth column indicates the species in which the interaction was observed; we use the species shorthand notations A.t. (*Arabidopsis thaliana*), V.f. (*Vicia faba* L.), C.c. (*Commelina communis*). X.l. (*Xenopus laevis* oocytes), N.b. (*Nicotiana benthamiana*), V.r. (*Vigna radiata*), S.l. (*Solanum lycopersicum,* tomato), a.c.l. (animal cell lines). The sixth column indicates the references in which the interaction was reported. The seventh column contains a verbal summary and additional information on each interaction.

The eighth column contains the designation of the interaction in one of the five categories described in the main text, based on the evidence strength of the interaction:

(1) S (standing for “Specific”): the interaction is shown to be involved specifically (with evidence) in CO_2_ signaling.

(2) G (for “Generic”): the interaction is generic and is expected/assumed to be involved in CO_2_ signaling, yet no explicit evidence exists. A chemical reaction whose reactants are demonstrated to be present in CO_2_ signaling is an example of such a generic interaction.

(3) N (for “Not in CO_2_ signaling”): evidence exists that the interaction is not involved in CO_2_ signaling.

(4) IE (for “Insufficient Evidence”): there is insufficient evidence to support the involvement of the interaction in CO_2_ signaling.

(5) IC (for “Insufficient Connection”): there is evidence that supports the interaction being involved in high CO_2_ induced closure, but with insufficient connections to known CO_2_ signaling elements.

We use S and G evidence to construct the CO_2_ signaling network and exclude N, IE, and IC evidence.

The last column specifies the usage of S and G evidence, i.e., whether the evidence is used in model construction (C) or in model validation (V). We construct the model using direct interactions, plus a small fraction of indirect interactions without which a signaling element would be disconnected from the network. The rest of the evidence is used as validation. For N, IE and IC categories, the usage is not applicable, thus the cell is left blank.

The table is ordered primarily by Category in the order of decreasing relevance to network model construction and strength of evidence, i.e., in the order S-G-N-IE-IC. Within each category, the table is ordered alphabetically by the first column, Entity/Process A.

| **Row #** | **Entity/**  **Process A** | **Entity/**  **Process B** | **Int. effect** | **Int. type** | **Spe-cies** | **Ref** | **Comments/notes** | **Cate-gory** | **Usage** |
| --- | --- | --- | --- | --- | --- | --- | --- | --- | --- |
|  | HCO_3_^-^ | SLAC1 | Promotes | Not direct/ Direct | A.t.  X.l. | [1, 2] | Gas exchange experiments with *slac1*-transformed plants expressing mutated SLAC1 proteins revealed that the SLAC1 residue R256 is required for CO_2_ regulation of stomatal movements, but not for ABA-induced stomatal closure. Patch clamp analyses show that activation of S-type anion channels by CO_2_/HCO_3_, but not by ABA, was impaired, indicating the relevance of R256 for CO_2_ signal transduction. Molecular dynamics simulations suggest HCO_3_^-^ interaction with SLAC1.We interpret this evidence as HCO_3_^-^ activating SLAC1 via binding to residue R256. | S | C |
|  | ABI1 | Stomatal closure | Inhibits | Not direct | A.t. | [3] | Arabidopsis *abi1* mutant, defective in guard cell ABA signaling, is also compromised in its response to elevated CO_2_. | S | V |
|  | ABI2 | Stomatal closure | Inhibits | Not direct | A.t. | [3] | Arabidopsis *abi2* mutant, defective in guard cell ABA signaling, is also compromised in its response to elevated CO_2_. | S | V |
|  | ABI1 | SLAC1 | Inhibits | Indirect | A.t., X.l. | [4-6] | The HCO_3_^-^-induced activation of S-type anion channels is reduced in the dominant negative PP2C mutant *abi1-1*. ABI1 physically interacts with SLAC1. Since [5] showed that ABI1 inhibits SLAC1 by inhibiting OST1-SLAC1 activation, we assume ABI1-SLAC1 inhibition is indirect. | S | C |
|  | ABI2 | SLAC1 | Inhibits | Indirect | A.t., X.l. | [4-6] | The HCO_3_^-^-induced activation of S-type anion channels is reduced in the dominant negative PP2C mutant *abi2-1*. ABI2 physically interacts with SLAC1. Since [5] showed that ABI2 inhibits SLAC1 by inhibiting OST1-SLAC1 activation, we assume ABI1-SLAC1 inhibition is indirect. | S | C |
|  | CAs | HCO_3_^-^ | Promotes | Direct | A.t. | [7, 8] | The CO_2_-binding carbonic anhydrase proteins that catalyze the reversible reaction of CO_2_ + H_2_O -> HCO_3_^–^ + H^+^ function early in CO_2_ signaling. | S | C |
|  | CAs | RHC1 | Promotes  (assumed) | Direct | A.t.,  X.l. | [9, 10] | RHC1 can interact with CA4 and HT1 at the plasma membrane. | S | C |
|  | CO_2_ Porins | CO_2_ entry of cell | Promotes | Direct | A.t.,  X.l. | [2] | PIP2;1 is a conduit for CO_2_ to enter the guard cell: it interacts with CA4 and confers CO_2_ sensitivity to SLAC1 channel activation in oocytes, provided either OST1 or CPK6 or CPK23 are also present.  We interpret this evidence as existence of “CO_2_ porins” similar to aquaporins that allow CO_2_ entry into the cell, and as PIP2;1 functioning as a CO_2_ porin. | S | C |
|  | CPKs | Stomatal closure | Promotes | Not direct | A.t. | [11] | The *cpk3/5/6/11/23* quintuple mutant plants, but not other analyzed *cpk* quadruple/quintuple mutants, are defective in high CO_2_-induced stomatal closure.  Light-mediated stomatal opening remained unaffected, and ABA responses was slowed in some experiments.  We interpret the evidence as a collective node “CPKs” participating in CO_2_-induced closure. | S | V |
|  | Cytosolic Ca^2+^ | Stomatal closure | Promotes | Not direct | A.t. | [12] | Low/high CO_2_ transitions modulate the cytosolic Ca^2+^ transient pattern in Arabidopsis guard cells. Inhibition of cytosolic Ca^2+^ transients by Ca^2+^ chelator or by not supplying external Ca^2+^ attenuated high CO_2_-induced stomatal closure, and also revealed a Ca^2+^-independent phase of the CO_2_ response. | S | V |
|  | HCO_3_^-^ | RHC1 | Promotes | Indirect | A.t.  X.l. | [9] | RHC1 is required for bicarbonate activation of S-type anion channels in Arabidopsis guard cells. | S | C |
|  | High CO_2_ | HCO_3_^–^ | Promotes | Direct | A.t. | [7, 8, 13] | The CO_2_-binding carbonic anhydrases that catalyze the reversible reaction of CO_2_ + H_2_O -> HCO_3_^–^ + H^+^ function early in CO_2_ signaling based on *ca1ca4* double mutants’ loss of high CO_2_-induced stomatal closure. | S | C |
|  | High CO_2_ | Inhibition of HT1 by MPKs | Promotes | Indirect | A.t. | [14, 15] | Elevated CO_2_ triggers interaction of the MAP kinases MPK4/MPK12 with the HT1 protein kinase, thus inhibiting HT1 kinase activity. | S | C |
|  | High CO_2_ | ROS | Promotes | Not direct | A.t. | [16] | Stomatal closure induced by elevated CO_2_ is inhibited by ROS scavengers Tiron and Tempol.  Elevated CO_2_ stimulates an increase in guard cell ROS concentration (measured by H_2_DCFDA fluorescence) that is blocked in the presence of Tempol or Tiron. | S | V |
|  | High CO_2_ | RbohD/F | Promotes | Not direct | A.t. | [16] | Elevated CO_2_ concentration induced stomatal closure is disrupted in the *rbohD rbohF* double mutant. Taken together with the evidence that elevated CO_2_ stimulates an increase in guard cell ROS concentration, we assume that high CO_2_ promotes the activity of RbohD/F. | S | V |
|  | High CO_2_ | cytosolic Ca^2+^ | Promotes | Not direct | C.c. | [17] | Elevated CO_2_ induces elevated Ca^2+^_cyt_. | S | V |
|  | High CO_2_ | K^+^ efflux | Promotes | Not direct | V.f | [18] | Elevated CO_2_ concentration causes stomatal closure movement and K^+^efflux. | S | V |
|  | High CO_2_ | Anion efflux | Promotes | Not direct | V.f. | [18] | Elevated CO_2_ concentration causes stomatal closure movement and Anion efflux. | S | V |
|  | High CO_2_ | Depolarization | Promotes | Not direct | V.f. | [18] | Elevated CO_2_ concentration causes stomatal closure movement and membrane depolarization. | S | V |
|  | High CO_2_ | NO production | Promotes | Not direct | S.l. | [19] | NITRATE REDUCTASE-silenced tomato has reduced CO_2_ induced NO production. NIA1/2 are the relevant nitric reductases. | S | V |
|  | High CO_2_ | H^+^-ATPase | Inhibits | Not direct | A.t. | [20] | CO_2_ induces the dephosphorylation of the guard-cell plasma membrane H^+^-ATPase, inactivating the H^+^-ATPase. | S | V |
|  | HT1 | OST1 | Inhibits | Direct | A.t., X.l. | [9, 21] | HT1 phosphorylates and inactivates OST1.  HT1 inhibits the activation of the SLAC1 anion channel by the protein kinase OST1 (OPEN STOMATA1) and the pseudokinase GUARD CELL HYDROGEN PEROXIDE-RESISTANT1 (GHR1) in *Xenopus laevis* oocytes.  We interpret this as HT1 inhibiting bothOST1_ nodes in the network model. | S | C |
|  | HT1 | Stomatal closure | Inhibits | Not direct | A.t.,  X.l. | [21-23] | The *ht1-2* mutant has low stomatal conductance and is insensitive to CO_2_; the *ht1-8D* dominant mutant has high stomatal conductance and is insensitive to CO_2_. | S | V |
|  | HT1 | CBC1/2 | Inhibits (assumed) | Direct | A.t. | [14, 24] | CBC1/CBC2 interact with and are phosphorylated by HT1. The *cbc1 cbc2* mutant is irresponsive to high CO_2_, with low stomatal conductance.  Since CBC1/2 are found to regulate SLAC1 in stomatal opening, although its regulation of SLAC1 in stomatal closure is not explicitly explored, we assume CBC1/2 inhibit SLAC1 in stomatal closure. | S | C |
|  | MPK12 | HT1 | Inhibits | Direct | A.t.,  X.l. | [14, 21, 25] | MPK12 interacts with the protein kinase HT1 and functions as an inhibitor of HT1;  HT1 inhibits SLAC1 currents induced by OST1 and GHR1 in oocytes, and this is counteracted by MPK12. | S | C |
|  | MPK4/12 | Stomatal closure | Promotes | Not direct | A.t.,  X.l. | [10, 14, 21, 25] | *mpk12 mpk4GC* homozygous double mutants lack CO_2_-induced stomatal responses. | S | V |
|  | NIA1/2 | High CO_2_ induced NO production | Promotes | Not direct | S.l. | [19] | NITRATE REDUCTASE-silenced tomato has reduced CO_2_ induced NO production. NIA1/2 are the relevant nitric reductases. | S | C |
|  | NO | High CO_2_ induced stomatal closure | Promotes | Not direct | S.l. | [19] | Elevated CO_2_-induced stomatal closure was dependent on the production of NITRATE REDUCTASE (NR)-mediated NO in guard cells in an ABA-independent manner. | S | V |
|  | OST1 | Stomatal closure | Promotes | Not direct | A.t. | [8] | *ost1* loss-of-function alleles showed strongly impaired CO_2_-induced stomatal closing and HCO_3_^−^ activation of anion channels. | S | V |
|  | QUAC1 | Stomatal closure | Promotes | Not direct | A.t. | [26, 27] | *QUAC1* KO has close-to-normal CO_2_ response; *SLAC1* KO + *QUAC1* KO double mutant has more impaired CO_2_ response than *SLAC1* KO. | S | V |
|  | RbohD/F | ROS | Promotes | Direct | A.t. | [16] | Rboh (D and F) enzymes are responsible for production of ROS. This paper confirms the roles of Rboh and ROS in CO_2_ signaling. | S | C |
|  | RHC1 | HT1 | Inhibits | Direct | A.t.,  X.l. | [9] | RHC1 can interact with CA4 and HT1 in the plasma membrane;  RHC1 removes the inhibitory effect of HT1 on SLAC1 activation by OST1. | S | C |
|  | SLAC1 | Stomatal closure | Promotes | Not direct | A.t. | [28] | *slac1* mutations impair CO_2_-, ABA- and dark-induced stomatal closure. | S | V |
|  | 8-Nitro-cGMP | ADPRc | Promotes | Not direct | A.t. | [29] | An antagonist of ADPRc abolishes 8-nitro-cGMP–induced stomatal closure. The research context is for ABA/NO induced stomatal closure. We assume this interaction is involved in CO_2_ signaling because NO is found to be produced under CO_2_, and this regulation is downstream of the NO pathway. | G | C |
|  | 8-Nitro-cGMP | Stomatal closure | Promotes | Not Direct | A.t. | [29] | 8-Nitro-cGMP promotes stomatal closure in a dose-dependent manner.  We assume this interaction is involved in CO_2_ signaling because NO is found to be produced under CO_2_. | G | V |
|  | ABI1 | OST1 | Inhibits | Direct | A.t. | [30-32] | ABI1 physically interacts with OST1 and inhibits its kinase activity. | G | C |
|  | ABI2 | OST1 | Inhibits | Direct | A.t. | [32] | ABI2 physically interacts with OST1 and inhibits its kinase activity. | G | C |
|  | ABI2 | SLAC1 | Inhibits | Direct | A.t.,  X.l. | [6] | ABI2 inhibits CPK-induced activation of SLAC1. | G | C |
|  | AnionEM | Depolarization | Promotes | Direct | V.f | [33] | Anion efflux across the plasma membrane promotes plasma membrane depolarization. | G | C |
|  | AnionEM | H_2_O Efflux | Promotes | Direct | A.t. |  | The efflux of anions is required for H_2_O efflux. | G | C |
|  | AtMPK9 (MPKs node) | AtMPK9 | Promotes | Direct | A.t. | [34] | AtMPK9 maintains its activity through auto-phosphorylation. | G | C |
|  | Ca^2+^ | Ca^2+^ATPase | Promotes | Direct | A.t. | [35] | Ca^2+^ promotes Ca^2+^ ATPase activity. | G | C |
|  | Ca^2+^ | KEV | Promotes | Not direct | V.f | [36] | Ca^2+^ induces K^+^ release through K^+^-permeable channels in the tonoplast. | G | C |
|  | Ca^2+^ | H^+^ ATPase | Inhibits | Direct | V.f | [37] | The H^+^ ATPase is inhibited by cytosolic calcium concentration increase. | G | C |
|  | Ca^2+^ | Depolarization | Promotes | Direct | A.t. | [38] | Ca­­^2+^ influx across the plasma membrane promotes plasma membrane depolarization. | G | C |
|  | Ca^2+^ | CPKs | Promotes | Direct | A.t. | [39-42] | Cytosolic calcium activates CPKs. | G | C |
|  | Ca^2+^ATPase | Ca^2+^_cyt_ | Inhibits | Direct | A.t. | [35] | The Ca­­^2+^ ATPase pumps Ca^­­2+^ from the cytosol to the apoplast. | G | C |
|  | Ca^2+^_cyt_ | Stomatal closure | Promotes | Not direct | C.c. | [43] | Increasing the concentration of Ca^2+^_cyt_ induces stomatal closure. | G | V |
|  | Ca^2+^_cyt_ | Slow anion channels (SLAC1) | Promotes | Not direct | A.t. | [44-47] | Increase the concentration of Ca^2+^_cyt_ increases the activity of slow anion channels. | G | V |
|  | cADPR | CIS | Promotes | Not Direct |  | [48, 49] | cADPR is an important signaling molecule leading to Ca­­^2+^ release from internal stores.  We assume that cADPR is involved in CO_2_ signaling because NO is found to be produced under CO_2_, and this regulation is downstream of the NO pathway. | G | C |
|  | cADPR | 8-nitro-cGMP-induced stomatal closure | Promotes | Not direct | A.t. | [29] | Cyclic adenosine-5’-diphosphate-ribose (cADPR) is a second messenger that modulates intracellular Ca^2+^ levels. Application of an antagonist of cADPR production (nicotinamide or 8-bromo-cADPR) inhibits 8-nitro-cGMP-mediated stomatal closure. These two inhibitors also inhibit NO-mediated stomatal closure.  We assume this effect is involved in CO_2_ signaling because NO was observed to be produced under CO_2_. | G | C |
|  | CaIM | Ca^2+^_cyt_ | Promotes | Direct |  | [50-53] | CaIM (Ca^2+^ Influx across the plasma Membrane) causes increase of cytosolic Ca^2+^. | G | C |
|  | cGMP | 8-nitro-cGMP | Promotes | Direct | A.t. | [29] | cGMP is the substrate for 8-nitro-cGMP synthesis.  We assume cGMP to be involved in CO_2_ signaling because NO is found to be produced under CO_2_, and this regulation is downstream of the NO pathway. | G | C |
|  | CIS | Ca^2+^_cyt_ | Promotes | Direct | V.f, C.c. | [54, 55] | CIS causes increase of cytosolic Ca^2+^.  Ref. [54] applied U-73122, which inhibited both ABA-induced oscillations in Ca^2+^_cyt_ and stomatal closure. In contrast, U-73122 did not inhibit external Ca^2+^-induced oscillations in guard-cell Ca^2+^_cyt_ and stomatal closure.  There is no specific CO_2_ evidence, but we know any Ca^2+^ release from stores will increase Ca^2+^ in the cytosol, so we assume this is “G”. | G | C |
|  | CIS | ABA-induced stomatal closure | Promotes | Not direct | A.t. | [56] | Pharmacological inhibition of the cADP-ribose/ryanodine receptor or the IP3 receptor negatively affects ABA-induced stomatal closure. As both receptors are likely to control the release of Ca^2+^ from intracellular stores, this finding suggests that intracellular Ca^2+^ stores participate in guard cell ABA signaling.  We assume that CIS happens under CO_2_ signaling as a result of CIS being downstream of NO, which is observed to be produced under high CO_2_. This assumption is necessary for NO-induced closure in the model. | G | C |
|  | CPK21 | SLAC1 | Promotes | Direct | X.l., A.t. | [6] | CPK21 directly interacts with SLAC1 and stimulates its activity in Arabidopsis and in *Xenopus laevis* oocytes.  However, the removal of the SLAC1 N terminal (containing CPK’s phosphorylation site) doesn’t impair CO_2_ induced closure, suggesting that CPKs are not necessary in CO_2_ induced closure.  Combining all evidence on CPKs, we interpret it as a collective node “CPKs” participating in CO_2_-induced closure. | G | C |
|  | CPK23 | SLAC1 | Promotes | Direct | X.l., A.t. | [6] | CPK23 directly interacts with SLAC1 and stimulates its activity in Arabidopsis and in *Xenopus laevis* oocytes.  However, the removal of the SLAC1 N terminal (containing CPK’s phosphorylation site) doesn’t impair CO_2_ induced closure, suggesting that CPKs are not necessary in CO_2_ induced closure.  Combining all evidence on CPKs, we interpret it as a collective node “CPKs” participating in CO_2_-induced closure. | G | C |
|  | CPK3/21 | SLAC1 | Promotes | Direct | A.t., X.l. | [6, 39, 47] | Both CPK3 and CPK21 physically interact with SLAC1 and activate SLAC1 activity.  Combining all evidence on CPKs, we interpret it as a collective node “CPKs” participating in CO_2_-induced closure. | G | C |
|  | CPK3/21 | CPK3/21 | Promotes | Direct | A.t. | [57] | CPK3 and other CPKs maintain their activity by auto-phosphorylation. | G | C |
|  | CPK6 | SLAC1 | Promotes | Direct | X.l.,  A.t. | [39, 47, 58] | CPK6 physically interacts with SLAC1 and activates SLAC1 activity.  Combining all evidence on CPKs, we interpret it as a collective node “CPKs” participating in CO_2_-induced closure. | G | C |
|  | CPK6,  CPK23 | SLAC1 | promotes | Direct | A.t., X.l. | [2, 6] | Co-expression of βCA4 and PIP2;1 with OST1-SLAC1 or CPK6/23-SLAC1 in oocytes enabled extracellular CO_2_ enhancement of SLAC1 anion channel activity.  We interpret the evidence as a collective node “CPKs” participating in CO_2_-induced closure. | G | C |
|  | Depolarization | KOUT | Promotes | Direct | A.t. | [59] | K+ efflux through outwardly rectifying K+ channels requires membrane depolarization. | G | C |
|  | External Ca^2+^ | Stomatal closure | Promotes | Not direct | A.t. | [60] | Ca­­^2+^ -induced closure is very similar to high CO_2_ induced closure in magnitude; CO_2_-induced stomatal closing is strongly impaired under conditions that prevent intracellular Ca^2+^ elevation. | G | V |
|  | GHR1 | CPK3 | Interacts, direction unknown  (assumed promotion) | Direct | A.t. | [61] | Biochemical analyses suggested that GHR1-mediated activation of SLAC1 occurs via interacting proteins and that CPK3 interacts with GHR1.  Loss of RCD7/GHR1 impairs stomatal closure. | G | C |
|  | GHR1 | SLAC1 | Promotes | Direct | A.t., X.l. | [62] | GHR1 physically interacts with SLAC1. Upon co-expression in *Xenopus laevis* oocytes, GHR1 physically interacts with SLAC1 and activates SLAC1. *ghr1* mutation impairs the ABA- and ROS activation of anion channels. | G | C |
|  | GHR1 | ROS activation of CaIM  (Ca^2+^ channels) | Promotes | Not direct | A.t. | [62] | *ghr1* mutation impairs ABA- and ROS (H_2_O_2_)-activation of Ca^2+^ channels in Arabidopsis thaliana. It is unknown whether GHR1 physically interacts with Ca^2+^ channels. We assume that ROS activates GHR1 and GHR1 activates CaIM.  This is assumed to be involved in CO_2_ signaling, as a result of ROS being produced under CO_2_. | G | C |
|  | GHR1 | Activation of SLAC1 by ROS | Promotes | Not Direct | A.t. | [62] | GHR1 is a positive regulator of ABA and ROS (H_2_O_2_) mediated stomatal closure. *ghr1* mutation impairs ABA or ROS mediated activation of SLAC1, Ca^2+^ channels and promotion of stomatal closure.  No direct interaction between ROS and GHR1 has been shown. Taken together with the fact that GHR1 interacts with and activates SLAC1, we infer that ROS indirectly promotes GHR1.  We assumed GHR1 to be involved in CO_2_ signaling as a result of ROS being produced under CO_2_. | G | C |
|  | GTP | cGMP | Promotes | Direct | A.t. | [63] | GTP is the substrate for cGMP production. cGMP can be generated from GTP in vitro, in an NO-dependent manner.  We assume this to be involved in CO_2_ signaling because NO is found to be produced under CO_2_, and this regulation is related to the targets of the NO pathway. | G | C |
|  | H^+^ ATPase | Depolarization | Inhibits | Direct | A.t. | [64, 65] | H^+^ ATPase activity negatively regulates plasma membrane depolarization. | G | C |
|  | HAB1 | OST1 | Inhibits | Direct | A.t. | [32] | HAB1 physically interacts with OST1 and inhibits its kinase activity. | G | C |
|  | HT1 | GHR1 | Inhibits | Direct | A.t.,  X.l. | [9, 21] | HT1 inhibits the activation of the SLAC1 anion channel by GHR1 in *Xenopus laevis* oocytes. | G | C |
|  | K^+^ efflux | H_2_O efflux | Promotes | Direct | A.t. | [59] | K^+^ efflux is required for H_2_O efflux. | G | C |
|  | K^+^ efflux | Depolarization | Inhibits | Direct | A.t. | [59] | K^+^ efflux across the membrane negatively regulates plasma membrane depolarization. | G | C |
|  | KEV | K^+^ efflux | Promotes | Direct | V.f | [36] | Sustained efflux of K^+^ from the guard cell requires K^+^ efflux from the vacuole to the cytosol. | G | C |
|  | KEV | Depolarization | Promotes | Direct | V.f | [36] | Release of K^+^ from the vacuole promotes plasma membrane depolarization. | G | C |
|  | KOUT | K^+^ efflux | Promotes | Direct | A.t. | [59] | Outwardly rectifying K^+^ channels (KOUT) promote K^+^ efflux from the cytosol to the apoplast. | G | C |
|  | MPK9/12 | ROS-mediated stomatal closure | Promotes | Not direct | A.t. | [66] | The *mpk9 mpk12* double mutants are impaired in ROS-mediated stomatal responses. No direct interaction between MPK9/12 and ROS has been shown.  We assume this to be involved in CO_2_ signaling as a result of ROS being produced under CO_2_ | G | V |
|  | MPK9/12 | Ca^2+^ induced activation of SLAC1 | Promotes | Not direct | A.t. | [66] | MAP kinases MPK9 and MPK12 play important roles in ABA and Ca^2+^ activation of S-type anion channels.  Direct interaction between MPK9/12 and anion channels has not been shown. We assume that Ca^2+^ promotes MPKs and MPKs promote SLAC1. | G | V |
|  | NAD^+^ | cADPR | Promotes | Direct | A.t. | [67] | NAD^+^ is a coenzyme that is required for cADPR production.  This is assumed to be involved in CO_2_ signaling because NO is found to be produced under CO_2_, and this regulation is downstream of the NO pathway. | G | C |
|  | NADPH | ROS (H_2_O_2_) | Promotes | Direct |  |  | NADPH is a coenzyme that is required for ROS (H_2_O_2_) production by RBOH (NADPH oxidase). | G | C |
|  | NADPH | NO | Promotes | Direct | A.t. | [68] | NADPH is a coenzyme that is required for NO production by NIA1/2.  We assume NADPH is involved in CO_2_ signaling because ROS and NO are found to be produced under CO_2_. | G | C |
|  | NIA1 and NIA2 (NIA1/2) | ROS induced NO production | Promotes | Not direct | A.t. | [69] | In the NIA1/2 double mutant (loss-of-function), *nia1 nia2*, ROS fails to induce NO production.  Taken together with the fact that NIA1 and NIA2 are the enzymes responsible for NO production, we infer that ROS promotes the activity of NIA1/2.  ROS (H_2_O_2_) does not enhance nitrate reductase activity in vitro. This suggests that ROS does not interact directly with NIA1/NIA2.  We assume NIA1 and NIA2 to be involved in CO_2_ signaling because ROS and NO are produced under CO_2_. | G | C |
|  | NIA1/2 | NO | Promotes | Direct | A.t. | [68] | NIA1 and NIA2 enzymes are required for NO production.  We assume this interaction to be involved in CO_2_ signaling because ROS and NO are found to be produced under CO_2_. | G | C |
|  | Nitrite | NO | Promotes | Direct | A.t. | [68] | Nitrite is a substrate required for NO production by NIA1 and NIA2 enzymes.  The reference is focused on ABA and Nitrite induced closure; but we assume that this interaction is involved in CO_2_ signaling because NO is found to be produced under CO_2_. | G | C |
|  | NO | NOGC1 | Promotes | Direct | A.t. | [63] | NO binds to NOGC1 and promotes the enzyme activity of NOGC1, which in turn accelerates the production of cGMP.  This is assumed to be involved in CO_2_ signaling because NO is found to be produced under CO_2_. | G | C |
|  | NO | 8-Nitro-cGMP | Promotes | Not direct | A.t. | [29] | Application of NO (NO donors: NOC5 and SNAP) increases production of 8-nitro-cGMP in guard cells. Application of NO scavenger (cPTIO) reduces ABA-and NO-mediated production of 8-nitro-cGMP. Application of ODQ (GC inhibitor) causes reduction of NO-mediated production of 8-nitro-cGMP. But, simultaneous application of NOC5 (NO donor) and ODQ (GC inhibitor) and cGMP, guard cells show higher level of 8-nitro-cGMP production. These findings suggest that NO promotes the biosynthesis of 8-nitro-cGMP.  We assume this to be involved in CO_2_ signaling because NO is found to be produced under CO_2._ | G | C |
|  | NO | KOUT | Inhibits | Direct | V.f | [70] | Current-voltage assays indicate that outwardly rectifying K^+^ channels are inhibited by NO.  This is assumed to be involved in CO_2_ signaling because NO is found to be produced under CO_2_. | G | C |
|  | NO | Closure | Promotes | Not direct | V.f., | [71, 72] | NO (Applying NO donor) promotes stomatal closure.  This is assumed to be involved in CO_2_ signaling because NO is found to be produced under CO_2_. | G | V |
|  | NO | cGMP | Promotes | Not direct | A.t. | [73] | NO promotes cGMP production in Arabidopsis seedlings. This is assumed as involved in CO_2_ signaling because NO is found to be produced under CO_2_. | G | C |
|  | NOGC1 | cGMP | Promotes | Direct | A.t. | [63] | AtNOGC1 is a guanylate cyclase that binds to NO. NOGC1 is responsible for the production of cGMP.  This is assumed as involved in CO_2_ signaling as a downstream of NO, as NO is found to be produced under CO_2_. | G | C |
|  | NOGC1 | 8-nitro-cGMP | Promotes | Not direct | A.t. | [29] | Guard cells of the *nogc1* mutant show stomatal closure in response to 8-nitro-cGMP. Guard cells of *nogc1* mutant do not show stomatal closure in response to ABA. These findings suggest that 8-nitro-cGMP is downstream of NOGC1 in guard cell ABA signaling.  This is assumed as involved in CO_2_ signaling as a result of NO being produced under CO_2_. | G | C |
|  | NOGC1 | NO-induced stomatal closure | Promotes | direct | A.t. | [29] | Guard cells of the *nogc1* mutant do not show NO-induced stomatal closure, indicating that NOGC1 is required for NO-induced stomatal closure.  This is assumed as involved to be CO_2_ signaling as a result of NO being produced under CO_2_. | G | C |
|  | OST1 | SLAC1 | Promotes | Direct | A.t. | [5, 46, 74] | SLAC1 (slow anion channel 1) is required for ABA-mediated stomatal closure. OST1 physically interacts with SLAC1. OST1 activates SLAC1 by phosphorylation.  Despite OST1 interaction and phosphorylation of SLAC1 being generic, recently no elevation of OST1 kinase activity was observed under high CO_2_ in ref. 74.  We create two nodes, OST1_minimum and OST1_activated in our model, to reflect these observations. | G | C |
|  | OST1 | QUAC1 | Promotes | Direct | A.t., X.l. | [27, 75, 76] | QUAC1 (AtALMT12) encodes a rapid anion channel. OST1 physically interacts with QUAC1. OST1 interaction causes promotion of QUAC1 activity.  QUAC1 (AtALMT12) is required for ABA-induced, CO_2_-induced and calcium-mediated stomatal responses. | G | C |
|  | OST1 | RbohD/F (RBOH) | Promotes | Direct | A.t., a.c.l. | [53, 77-79] | OST1 physically interacts with both RbohD and RbohF. OST1 kinase phosphorylates NADPH oxidase subunits RbohD/F; OST1 promotes ROS production; RbohD and RbohF are required for ABA-mediated ROS production.  This regulation is assumed “G” as ROS is produced in high CO_2_ response. | G | C |
|  | OST1 | PIP2;1 | Promotes | Direct | A.t. | [80] | OST1-mediated phosphorylation activates aquaporin Plasma membrane Intrinsic Protein 2;1 (PIP2;1) in guard cells in response to ABA.  The paper has only ABA evidence; but OST1 phosphorylation of PIP2;1 could be generic. | G | C |
|  | OST2/AHA1  H+-ATPase | ABA-promotion of stomatal closure | Inhibits | Not direct | A.t. | [65] | OST2 encodes the plasma membrane bound H+-ATPase AHA1. OST2/AHA1 is expressed in guard cells.  Constitutive activation of this gene impairs ABA promotion of stomatal closure. We assume the mechanism behind this effect is that the constitutive activation of H^+^-ATPase leads to efflux of cations thus the hyperpolarization of the plasma membrane, and it will. | G | V |
|  | PIP2;1  (Aquaporin) | H_2_O Efflux | Promotes | Direct | A.t. | [80] | Aquaporin (PIP2;1) facilitates water efflux during ABA induced stomatal closure. We assume that this process also happens under high CO_2_ induced closure. | G | C |
|  | QUAC1 | AnionEM | Promotes | Direct | A.t. | [27, 81] | QUAC1 facilitates efflux of malate anions.  QUAC1 is shown to have some effect in CO_2_ induced closure. | G | C |
|  | QUAC1 | Ca^2+^ induced closure | Promotes | Not direct | A.t. | [76] | The QUAC1 loss-of-function mutant shows reduced Ca^2+^-induced stomatal closure. We assume that Ca^2+^ promotes QUAC1. | G | C |
|  | ROS | 8-Nitro-cGMP | Promotes | Not direct | A.t. | [29] | Suppression of ROS production by application of different reagents (reducing agent, DTT; superoxide scavenger, Trion; and H2O2 scavenger, catalase) reduces production of 8-nitro-cGMP.  The *abi1-1* mutant (dominant negative), which does not show ROS production in response to ABA, also does not induce production of 8-nitro-cGMP in response to ABA. These findings suggest that ROS are required for the production of 8-nitro-cGMP.  We assume this to be involved in CO_2_ signaling because NO is found to be produced under CO_2_. | G | C |
|  | ROS | KOUT | Inhibits | Not direct | V.f | [82] | Outwardly rectifying K^+^ channels are inhibited by ROS. | G | C |
|  | ROS | H^+^ ATPase | Inhibits | Not direct | V.f | [83] | The H^+^ ATPase is inhibited by reactive oxygen species. | G | C |
|  | ROS | ABI1 | Inhibits | Direct | A.t. | [84] | ROS inhibit ABI1 activity. | G | C |
|  | ROS | HAB1 | Inhibits | Direct | A.t. | [85] | ROS oxidize H_2_O_2_-sensitive thiols and inhibit HAB1’s catalytic activity. | G | C |
|  | ROS | ABI2 | Inhibits | Direct | A.t. | [86] | ABI2 is negatively regulated by ROS. | G | C |
|  | ROS | NO | Promotes | Not direct | A.t. | [69] | ABA-induced NO generation is dependent on ABA-induced ROS (H_2_O_2_) production.  This is assumed to be involved in CO_2_ signaling because both ROS and NO are produced under CO_2_. | G | C |
|  | ROS | Stomatal closure | Promotes | Not Direct | A.t.,V.f. | [62, 87, 88] | Application of ROS promotes stomatal closure.  Application of ROS can also cause stomatal closure in *gpa1* mutant. | G | V |
|  | SLAC1 | AnionEM | Promotes | Direct | A.t. | [5, 81] | SLAC1 facilitates efflux of chloride and nitrate anions. | G | C |
|  | SLAH3 | AnionEM | Promotes | Direct | A.t. | [81, 89] | SLAH3 facilitates efflux of nitrate anions. | G | C |
|  | ABA | Vacuolar acidification | Promotes | Not direct | A.t.,  V.f. | [90] | The vacuolar lumen is acidified during ABA-mediated stomatal closure. Loss of vacuolar pump activities delays stomatal closure in response to ABA.  Vacuolar acidification is assumed not to happen under CO_2_ signaling because no pH_c_ change is observed under high CO_2_. | N |  |
|  | ABI1 | ABA induced pH_c_ increase | Inhibits | Not direct | A.t. | [91] | ABA does not induce cytosolic alkalization in *abi1-*dominant mutant.  This regulation is not involved in high CO_2_ signaling, as pH_c_ does not increase under high CO_2_. | N |  |
|  | ABI2 | ABA induced pH_c_ increase | Inhibits | Not direct | A.t. | [91] | ABA does not induce cytosolic alkalization in *abi2-1* dominant mutant.  This regulation is not involved in high CO_2_ signaling, as pH_c_ does not increase under high CO_2_. | N |  |
|  | Ca^2+^ | pH_c_ | Promotes | Not direct | A.t. | [91] | Exogenous Ca^2+^ induces cytosolic alkalization.  This is confirmed to not be relevant to CO_2_ signaling, as pH_c_ is observed not to increase in response to elevated CO_2_. | N |  |
|  | CO_2_ | Cytosolic acidification | Promotes | Not direct | Multiple | [92] | CO_2_ causes cytosolic acidification in *Rumex*, potato, and *Pelargonium zonale*.  We interpret this as consistent with the lack of cytosolic pH increase. | N |  |
|  | High CO_2_ | Cytosolic pH increase | Does not promote | Not direct | V.f. | [8, 18] | Cytosolic pH increase was not observed under high CO_2_. | N |  |
|  | OST1 | HT1 | Does not interact | Direct | A.t.,  X.l. | [9, 21] | OST1 does not phosphorylate HT1. | N |  |
|  | OST1 | ABA induced pH_c_ increase | Promotes | Not direct | A.t. | [91] | ABA does not induce cytosolic alkalization (pH_c_ increase) in the *ost1-2* mutant.  pH_c_ is known not to increase under CO_2_. | N |  |
|  | pH_c_  increase | Vacuolar acidification | promotes | Direct | V.f. | [90] | Inhibition of ABA-induced cytosolic alkalization by butyrate (a weak acid) application causes suppression of vacuolar acidification in response to ABA.  pH_c_ is observed not to increase under CO_2_ signaling. | N |  |
|  | pH_c_ increase | KOUT | Promotes | Not Direct | V.f | [93] | Voltage-current assays indicate that outwardly rectifying K^+^ channels are activated by cytosolic pH increase.  pH_c_ increase does not occur under high CO_2_. | N |  |
|  | pH_c_ increase | H^+^ ATPase | Inhibits | Not direct | N.b. | [94] | The H^+^ ATPase is inhibited by cytosolic H^+^ concentration decrease.  pHc increase does not occur under high CO_2_. | N |  |
|  | pH_c_ increase | ABI1 | Promotes | Direct | A.t. | [95] | pH increase activates enzyme activity of ABI1.  pH_c_ increase does not occur under high CO_2_. | N |  |
|  | pH_c_ increase | pH_c_ | Inhibits | Not direct | A.t., pea | [64, 96, 97] | Time course data indicate that in response to ABA, in guard cells, pH_c_ value increases quickly and then decreases gradually, subsequently stabilizing above the resting level. No signaling components have been identified.  pH_c_ increase does not occur under high CO_2_. | N |  |
|  | pH_c_ increase | ABA activation of RBOH | Promotes | Not Direct | A.t. | [97] | ABA-induced cytosolic alkalization (pH_c_ increase) is necessary for ROS production.  pH_c_ is known not to increase under high CO_2_. | N |  |
|  | pH_c_ increase | ABA activation of SLAC1 | Promotes | Not Direct | A.t. | [98] | Clamping cytosolic pH inhibits ABA activation of slow anion channel activity.  pH_c_ is known not to increase under high CO_2_. | N |  |
|  | pH_c_ increase | ABA-promotion of NO production | Promotes | Not direct | Pea | [99] | Guard cell pH rises after 6 min of ABA application, peaking at 18 min. NO production starts after 9 min of ABA application and peaks at 18 min. Application of butyrate (a weak acid) reduces cytosolic pH, decreases NO production and prevents stomatal closure in response to ABA. In contrast, application of methylamine (a weak base) enhances cytosolic alkalization and promotes stomatal closure in response to ABA. | N |  |
|  | PtdIns(3,5)P2 | ABA-induced vacuolar acidification | Promotes | Not direct | A.t.,  V.f. | [90] | By employing pharmacological approaches the authors have shown that inhibition of PtdIns(3,5)P_2_ biosynthesis causes reduced ABA induction of vacuolar pH.  pH_c_ is known not to increase under high CO_2_, so we assume that vacuolar acidification does not happen under high CO_2_ either. | N |  |
|  | Vacuolar acidification | pH_c_ | Promotes | Direct | V.f. | [90] | By employing a pharmacological approach, the authors showed that vacuolar acidification is necessary for cytoplasmic alkalization (pH_c_ increase).  Inhibition of ABA-induced cytosolic alkalization by butyrate (a weak acid) application causes suppression of vacuolar acidification in response to ABA.  These above findings suggest that vacuolar acidification and cytosolic alkalization are interdependent during ABA-mediated stomatal closure.  This is known not to happen under CO_2_ because no pH_c_ change is observed under high CO_2_. | N |  |
|  | Vacuolar acidification | KEV | Promotes | Not Direct | V.f | [36] | Vacuolar acidification induces K^+^ efflux from the vacuole.  Vacuolar acidification is assumed not to happen under CO_2_ signaling because no pH_c_ change is observed under high CO_2_. | N |  |
|  | V-ATPase | Vacuolar acidification | Promotes | Direct | Yeast,  A.t.,  V.f. | [90, 100, 101] | The functioning of vacuolar pumps is essential for acidification of vacuole. In yeast, the vacuolar proton ATPase (V-ATPase) proton pump plays an important role in vacuolar acidification. Loss of vacuolar pump (V-ATPase) activity delays stomatal closure in response to ABA.  This is assumed not to be involved in the CO_2_ signaling because no pH_c_ change is observed under high CO_2_, thus vacuolar acidification is assumed to not happen. | N |  |
|  | V-PPase  (vacuolar pump) | Vacuolar acidification | Promotes | Direct | A.t.,  V.f. | [90] | Proton pumping vacuolar pyrophosphatase (V-PPase) uses energy of PPi hydrolysis to acidify the vacuole. Loss of vacuolar pump (V-PPase) activity delays stomatal closure in response to ABA.  This is assumed not involved in the CO_2_ signaling because no pH_c_ change is observed under high CO_2_, thus vacuolar acidification is assumed not happening. | N |  |
|  | ABA | RCARs | Promotes | Direct | A.t. | [30, 102-104] | RCARs are soluble ABA receptors. These proteins directly bind ABA. RCARs also mediate ABA-mediated stomatal closure.  The role of RCARs under CO_2_ signaling in under debate | IE |  |
|  | ABA | PEPC | Inhibits | Not direct | V.f | [105, 106] | ABA inhibits PEP carboxylase (PEPC) activity in guard cells. PEPC activity is hypothesized as an important regulatory feature of stomatal opening. | IE |  |
|  | ABA | PI3P5K | Promotes | Not direct | A.t. | [90] | ABA indirectly promotes PI3P5K activity. Rapid ABA-induced stomatal closure requires PtdIns(3,5)P2. | IE |  |
|  | ABA | SPHK1/2 | Promotes | Not direct | A.t. | [107-110] | SPHK1 and SPHK2 are sphingosine kinases. Guard cell sphingosine kinase activity is stimulated by ABA in Arabidopsis. There are no studies of SPHK signaling under high CO_2_. | IE |  |
|  | ABA | AtRAC1 | Inhibits | Not direct | A.t. | [111] | ABA treatment causes inactivation of AtRAC1 and promotion of actin reorganization in guard cells. | IE |  |
|  | ABA | Malate | Inhibits | Not direct | C.c. | [112] | ABA negatively regulates malate concentration by inducing malate breakdown. | IE |  |
|  | ABA | Actin reorganization | Promotes | Not direct | A.t. | [111] | ABA treatment promotes actin reorganization in guard cells.  There are no studies of actin reorganization under high CO_2_. | IE |  |
|  | ABA | Microtubule Depolymerization | Promotes | Not Direct | A.t.,  V.f. | [113] | Application of ABA (10 µM) causes reduction of resolved microtubule structures.  There are no studies of microtubule’s involvement under high CO_2_. | IE |  |
|  | ABH1 | CaIM | Inhibits | Not direct | A.t. | [114] | The *abh1* mutant shows greater increases in cytosolic Ca^2+^ in response to ABA compared to the wild type. | IE |  |
|  | ABI1 | AtRAC1 | Promotes | Not direct | A.t. | [111] | Both ABI and AtRAC1 are negative regulators of stomatal closure. The dominant negative form of AtRAC1 was able to recover ABA-induced stomatal closure in *abi1-1*(dominant negative mutant), so one may assume that ABI1 promotes AtRAC1.  Experiments are done on ABA-induced closure. No studies have investigated this under CO_2_ signaling. | IE |  |
|  | ABI1 | SLAH3 | Inhibits | Direct | A.t. | [89] | ABI1 inhibits CPK-mediated activation of SLAH3.  Although CPKs are involved in CO_2_ signaling, no studies have investigated SLAH3 under CO_2_ signaling. | IE |  |
|  | ABI1 | ABA activation of RBOH | Inhibits | Not direct | A.t. | [115] | Guard cells of the *abi1-1* mutant (dominant negative) do not show ABA induced ROS production. | IE |  |
|  | ABI1 | SLAC1 | Inhibits | Direct | A.t. | [6] | CPK21 and CPK23 mediated activation of SLAC1 is suppressed by ABI1.  This piece of evidence is insufficient as we know CPKs are not the main cause of SLAC1 activation. However, we do have other evidence that SLAC1 is suppressed by ABI1 under CO_2_. | IE |  |
|  | ABI1 | CPK3 and CPK6 activation of SLAC1 | Inhibits | Direct | A.t., X.l. | [39, 58] | ABI1 inhibits CPK3/6-mediated activation of SLAC1. (in vitro kinase assay)  This piece of evidence is insufficient as we know CPKs are not the main cause of SLAC1 activation. However, we do have other evidence that SLAC1 is suppressed by ABI1 under CO_2_. | IE |  |
|  | ABI2 | CPK6 and CPK23  activation of SLAC1 | Inhibits | Direct | A.t., X.l. | [6, 58] | ABI2 inhibits CPK6/23 mediated activation of SLAC1.  This piece of evidence is insufficient as we know CPKs are not the main cause of SLAC1 activation. | IE |  |
|  | Actin reorganization | CaIM | Promotes | Not direct | V.f | [116] | Actin reorganization promotes stretch-activated Ca^2+^ channels.  Experimental assays are mainly patch-clamp, and conductance measurements. | IE |  |
|  | AGB1 | Calcium-induced stomatal closure | Promotes | Not direct | A.t. | [117] | Stomatal movements of *agb1* mutants and *agb1/gpa1* double-mutants, as well as those of the *agg1agg2* Gγ double-mutant, were insensitive to extracellular Calcium. *AGB1* knockouts also lost [Ca^2+^]_cyt_ oscillations in response to extracellular Ca^2+^. | IE |  |
|  | AnionEM | Malate | Inhibits | Direct | A.t. | [27, 81] | Anion efflux (AnionEM) negatively regulates intracellular malate concentration by releasing malate from the cytosol. | IE |  |
|  | ARP2/3 complex | Actin reorganiza-tion | Promotes | Direct | A.t. | [118, 119] | ARP2 (Actin Related Protein C2) encodes the ARPC2 subunit of the ARP2/3 complex. Purified, active Arp2/3 complex binds to the sites of existing actin filaments and nucleates new ‘daughter’ filaments. The *arp2* mutant is deficient in ABA- and CaCl_2_-induced stomatal closure. This mutant does not show actin reorganization in response to ABA in guard cells. Upon addition of cytochalasin D (which induces depolymerization of actin filaments) *arp2* mutant guard cells show similar ABA-mediated stomatal response as wild type.  There is no study of actin reorganization under CO_2_ signaling. | IE |  |
|  | AtRAC1 | Actin reorganiza-tion | Inhibits | Not direct | A.t. | [111] | Expression of a dominant-positive mutant of AtRAC1 inhibits ABA-induced actin reorganization whereas expression of a dominant-negative mutant of AtRAC1 promotes actin reorganization in the absence of ABA.  There is no study of actin reorganization under CO_2_ signaling. | IE |  |
|  | AtSPP1 | S1P | Inhibits | Direct | A.t. | [120] | SPP1, a long-chain base 1-phosphatase, has been implicated as a negative regulator of S1P accumulation in plants. There is no study of S1P production under CO_2_ signaling. | IE |  |
|  | AtSPP1 | ABA-induced stomatal closure | Inhibits | Not direct | A.t. | [120] | Long-chain base 1-phosphates (LCBP) are sphingolipid metabolites. LCBPs are synthesized by LCB kinase and dephosphorylated by LCBP phosphatase and degraded by LCBP lyase.  AtSPP1 (Arabidopsis thaliana sphingoid phosphate phosphatase 1) is an LCBP phosphatase.  Guard cells of *atspp1* mutant show slightly enhanced stomatal closure compared to wild type in response to ABA.  There are no studies that investigate AtSPP1 being involved in CO_2_ signaling. | IE |  |
|  | AtTCTP | Microtubule depolymeriza-tion | Promotes | Direct | A.t. | [121] | Interaction between AtTCTP and microtubules promotes microtubule depolymerization.  There are no studies that investigate its involvement in CO_2_ signaling. | IE |  |
|  | AtTCTP | Ca^2+^-mediated stomatal closure | Promotes | Not Direct | A.t. | [121] | TCTP encodes a translationally controlled tumor protein (known as P23 in human) and belongs to a family of calcium- and tubulin-binding proteins. Plants overexpressing AtTCTP show faster Ca^2+^-mediated stomatal closure.  This is possibly generic regulation as calcium influx is generic; however, there are no studies that investigate this being involved in CO_2_ signaling. | IE |  |
|  | AtTCTP | ABA-mediated stomatal closure | Promotes | Not Direct | A.t. | [121] | Plants overexpressing AtTCTP show faster ABA-mediated stomatal closure.  There are no studies that investigate this being involved in CO_2_ signaling. | IE |  |
|  | Ca^2+^ | AtTCTP | Promotes | Direct | A.t. | [121] | Ca^2+^ promotes the interaction between AtTCTP and microtubules (in vitro protein-protein interaction analysis).  There are no studies of microtubule alteration in CO_2_ response. | IE |  |
|  | Ca^2+^ | PLDα1 (PLDα node) | Promotes | Direct | A.t. | [122] | Ca^2+^ is required for activation of PLDα1 and also promotes translocation of PLDα1 to the plasma membrane and tonoplast where lipid substrates are available for the enzyme.  There are no studies of the involvement of PLDs in high CO_2_ induced closure. | IE |  |
|  | Ca^2+^ | PLC | Promotes | Direct | A.t. | [123] | Ca^2+^ is required for PLC activity.  There are no studies of the involvement of PLDs in high CO_2_ induced closure. | IE |  |
|  | Ca^2+^ | V-ATPase | Promotes | Not Direct | A.t. | [124] | Ca^2+^_c_ has been implicated as a positive regulator of the V-ATPase.  (Calcium binds to CBL2 and CBL3; *cbl2 cbl3* double mutant exhibits reduced vacuolar H^+^-ATPase activity and elevated vacuolar pH;  measured total V-ATPase activity)  This would have been “Generic” regulation; but the only successor of V-ATPase is vacuolar acidification, which does not happen under high CO_2_ induced closure because pH_c_ doesn’t increase. | IE |  |
|  | Ca^2+^ | 8-nitro-cGMP-mediated stomatal closure | Promotes | Not Direct | A.t. | [29] | Application of cell-permeating Ca^2+^ chelator (BAPTA-AM) inhibits 8-nitro-cGMP-mediated stomatal closure. Similarly, application of BAPTA-AM inhibits NOC5- (NO donor) mediated stomatal closure. These findings suggest that Ca^2+^ is a required signaling component in 8-nitro-cGMP-mediated stomatal closure.  There are no studies that investigate this being involved in CO_2_ signaling. | IE |  |
|  | Ca^2+^ | ABA induction of KEV | Promotes | Not direct | C.c. | [125, 126] | Above-threshold level of Ca^2+^_c_ is required for ABA activation of KEV and K^+^ ion release from vacuole. Inhibition of Ca^2+^ influx through Ca^2+^-permeable channels and Ca^2+^ release from internal stores blocks K^+^ release from vacuoles.  There are no studies of the effect of inhibition of Ca^2+^ influx on CO_2_ induced NO production. | IE |  |
|  | Ca^2+^ | ABA-induced NO production | Promotes | Not direct | Pea | [99] | Application of EGTA (a calcium chelator) restricts ABA-induced NO production and stomatal closure.  There are no studies of calcium chelator’s effect on CO_2_ induced NO production. | IE |  |
|  | CPK23 | SLAH3 | Promotes | Direct | X.l. | [39, 89] | CPK23 interacts with SLAH3. CPK23 promotes SLAH3 activity.  There are no studies of SLAH3’s involvement in high CO_2_ induced closure. | IE |  |
|  | CPK3/21 | SLAH3 | Promotes | Direct | X.l. | [39, 89] | CPK3 and CPK21 interact with SLAH3.  There are no studies of SLAH3’s involvement in high CO_2_ induced closure. | IE |  |
|  | CPK6 | SLAH3 | Promotes | Direct | X.l. | [39] | CPK6 interacts with SLAH3. CPK6 promotes SLAH3 activity.  There are no studies of SLAH3’s involvement in high CO_2_ induced closure. | IE |  |
|  | CPKs | Calcium-induced stomatal closure | Promotes | Not direct | A.t. | [60] | Ca^2+^ oscillation-induced stomatal closing is partially impaired in knock-out mutations of several guard cell-expressed Ca^2+^-dependent protein kinases (CDPKs), including the *cpk4cpk11* double and *cpk10* mutants; however, abscisic acid-regulated stomatal movements remain relatively intact in the *cpk4cpk11* and *cpk10* mutants. | IE |  |
|  | Cytosolic Malate | H_2_O efflux | Inhibits | Not direct | C.c. | [112] | Malate is an osmoticum that inhibits H_2_O efflux by decreasing water potential in the cell.  There are no studies of malate’s involvement in high CO_2_ induced closure. | IE |  |
|  | DAG | PA | Promotes | Direct |  | [127] | DAG can be converted into PA by DAGK-mediated phosphorylation.  There are no studies of their involvement in high CO_2_ induced closure. | IE |  |
|  | DAGK | PA | Promotes | Direct |  | [127] | DAG can be converted into PA by DAGK-mediated phosphorylation.  There are no studies of their involvement in high CO_2_ induced closure. | IE |  |
|  | EMS1 | CAs | Promotes | Direct | A.t. | [128] | EMS1 biochemically interacts with βCA proteins. Loss of function of βCA genes caused defective tapetal cell differentiation, while overexpression of βCA1 led to the formation of extra tapetal cells. EMS1 phosphorylates βCA1 at four sites, resulting in increased βCA1 activity.  There has not yet been an investigation of EMS’s role in stomatal closure. | IE |  |
|  | ERA1 | ROP10 | Promotes | Not direct | A.t. | [129] | Localization of ROP10 to the plasma membrane is required ROP10 function. Functional ERA1 is required for plasma membrane localization of ROP10 indicating that ERA1 positively affects ROP10 function. The role of ROP10 or ERA1 in high CO_2_ induced closure has not yet been investigated yet. | IE |  |
|  | ERA1 | CaIM | Inhibits | Not direct | A.t. | [130] | At low ABA concentrations, greater increases in cytosolic Ca^2+^ and stomatal closure activation were observed in the *era1-2* loss-of-function mutant in comparison to the wild type. These observations suggest that ERA1 inhibits cytosolic Ca^2+^ influx. The role of ROP10 or ERA1 in high CO_2_ induced closure has not yet been investigated yet. | IE |  |
|  | GAPC1 and GAPC2 | PLDδ | Promotes | Direct | A.t. | [131] | GAPC1 and GAPC2 interact with PLDδ which in turn increases enzyme activity of PLDδ, to transduce the ROS signal.  There are no studies of PLDδ’s involvement in high CO_2_ induced closure. | IE |  |
|  | GAPC1, GAPC2 | ROS activation of PLDδ | Promotes | Direct | A.t. | [131] | Upon interaction with ROS, GAPC1/2 (Glyceraldehyde-3-phosphate dehydrogenases 1 and 2) interact with PLDδ and transduce the ROS (H_2_O_2_)-signal.  Knockout of GAPCs decreased ABA- and H2O2-induced activation of PLD and stomatal sensitivity to ABA.  There are no studies of PLD’s involvement in the CO_2_ response, though ROS induced activation of PLD could be generic. | IE |  |
|  | GCR1 | GPA1 | Inhibits | Direct | A.t. | [132] | GCR1 interacts with GPA1. Loss-of-function *gcr1* mutants show hypersensitivity in S1P induced stomatal responses, in contrast *gpa1* loss-of-function mutants show insensitivity. These findings suggesting that GCR1 is a negative regulator of GPA1.  There are no studies for GCR1’s involvement in high CO_2_ induced closure. | IE |  |
|  | GEF1, GEF4, GEF10 | ROP11 | Promotes | Direct | A.t. | [133, 134] | GEF1, GEF4, and GEF10 interact with ROP11.  Guanine nucleotide exchange factors (GEFs) regulate the function of ROPs. *gef1 ge4* double mutant and *gef1/4/10* triple mutants are hypersensitive to ABA-mediated stomatal responses. The *gef* double and triple mutants do not show different aperture size in the absence of exogenous ABA.  These interactions are assumed generic but the mutant behaviors are ABA specific. There are no studies of their involvement in high CO_2_ induced closure. | IE |  |
|  | GPA1 | ABA activation of RBOH | Promotes | Not direct | A.t. | [88] | RbohD and RbohF (RBOH) are vital components for ABA-induced ROS production. Guard cells of gpa1 mutant plants do not show significant ROS production in response to ABA. The role of GPA1 in high CO_2_ induced closure has not been reported yet. | IE |  |
|  | GPA1 | inward K^+^ channels | Promotes | Not direct | A.t. | [98] | *gpa1* mutants lack ABA inhibition of guard cell inward K^+^ channels. The relevance of this regulation to high CO_2_-induced closure is not studied. | IE |  |
|  | GPA1 | AnionEM | Promotes | Not direct | A.t. | [98] | *gpa1* mutants lack pH-independent ABA activation of anion channels. The relevance of this to high CO_2_-induced closure is not studied. | IE |  |
|  | GPA1 | PLDα1 | Promotes | Direct | A.t. | [135] | Addition of Gα to PLDα1 inhibited PLDα1 activity, whereas the PLDK564A mutation that disrupted the Gα-PLDα1 binding abolished the inhibition. GTP relieved the Gα inhibition of PLDα1 activity and also inhibited the binding between PLDα1 and Gα. Meanwhile, the PLDα1-Gα interaction stimulated the intrinsic GTPase activity of Gα.  There are no studies of PLDα’s involvement in high CO_2_ induced closure. | IE |  |
|  | GPA1 | S1P – induced closure | Promotes | Not direct | A.t. | [107] | GPA1 is required for S1P-mediated stomatal response.  There are no studies of S1P’s involvement in CO_2_ signaling. | IE |  |
|  | GPA1 | ABA-induced activation of Ca^2+^-permeable channels | Promotes | Not direct | A.t. | [88] | Guard cells of gpa1 mutant plants do not show ABA-activation of Ca^2+^-permeable channels suggesting that GPA1 is a positive regulator or mediator of ABA activation of Ca^2+^-permeable channels in guard cells.  There are no studies that investigate this regulation being involved in CO_2_ signaling. | IE |  |
|  | InsP3 | CIS | Promotes | Not direct | C. c. | [54] | InsP3 can release calcium from internal stores.  The release of Ins(1,4,5)P3 in guard cells has also been reported to inhibit the plasma-membrane inward K^+^ channel.  Also, this regulation might be direct: it’s unknown in plants, but known to be direct in other organisms.  There are no studies of InsP3’s involvement in high CO_2_ induced closure. | IE |  |
|  | InsP3 | InsP6 | Promotes | Direct |  | [136] | Triple phosphorylation of InsP_3_ yields InsP_6_.  There are no studies of InsP3 & InsP6’s involvement in high CO_2_ induced closure. | IE |  |
|  | InsP6 | CIS | Promotes | Not direct | V.f | [55] | InsP6 can release calcium from internal stores.  There are no studies of InsP6’s involvement in high CO_2_ induced closure. | IE |  |
|  | Microtubule depolymeri-zation | Stomatal closure | Promotes | Not direct | A.t. | [137] | Microtubule depolymerization is necessary for stomatal closure.  This regulation could be generic, but the paper focuses on ABA signaling, and there are no studies of microtubule depolymerization in high CO_2_ induced closure. | IE |  |
|  | Microtubule depolymeri-zation | Microtubule depolymeriza-tion | Promotes | Direct |  | [138] | Once started, microtubule depolymerization continues for a sustained period; a process termed catastrophe.  There are no studies of microtubule depolymerization in high CO_2_ induced closure. | IE |  |
|  | MRP5  (ATP binding cassette (ABC) protein) | ABA activation of Ca^2+^- channels | Promotes | Not direct | A.t. | [139] | Guard cells of *mrp5* mutant do not show ABA activation of Ca^2+^- channels. *mrp5* loss-of-function mutant plants show partially impaired ABA-induced stomatal closure.  There are no studies that investigate MRP5 being involved in CO_2_ signaling. | IE |  |
|  | MRP5 | Ca^2+^ activation of slow (S-type) anion channel | promotes | Not direct | A.t. | [139] | MRP5 encodes an ATP binding cassette (ABC) protein. *mrp5* mutant shows less pronounced Ca^2+^ activation of anion currents. *mrp5* mutant also does not show ABA activation of S-type anion currents.  There are no studies that investigate MRP5 being involved in CO_2_ signaling. | IE |  |
|  | NO | NtSyp121-Sp2-mediated inhibition of ABA-induced stomatal closure | Inhibits | Not Direct | N.b. | [140] | Application of NO donor, SNAP, elevates the intracellular Ca^2+^ level and partially blocks the inhibitory effect of NtSyp121-Sp2 fragment on ABA-induced stomatal closure. This finding indicates that expression of NtSyp121-Sp2 fragment inhibits stomatal closure by modulating Ca^2+^.  There are no studies that investigate NtSyp121-Sp2 being involved in CO_2_ signaling. | IE |  |
|  | NtSyp121-Sp2  (SNARE protein) | CaIM | Inhibits | Not Direct | N.b. | [140] | SNAREs, soluble NSF (N-ethylmaleimide-sensitive factor) attachment protein receptors, are membrane trafficking proteins that play important role in vesicle fusion and membrane trafficking. Expression of NtSyp121-Sp2 fragment inhibits gating (opening) of Ca^2+^ permeable channels. These findings implicate that SNARE proteins are positive regulators in Ca^2+^ channel gating.  This evidence is ABA specific. There are no studies that investigate its involvement in high CO_2_ signaling. | IE |  |
|  | NtSyp121-Sp2 | Ca^2+^ transient | Inhibits | Not Direct | N.b. | [140] | Expression of NtSyp121-Sp2 fragment inhibits the induction of intracellular Ca^2+^ transient. This suggests that NtSyp121 promotes Ca^2+^ increase.  This evidence is ABA-specific; there are no studies that investigate its involvement in CO_2_ signaling. | IE |  |
|  | NtSyp121-Sp2 | ABA-induced stomatal closure | Inhibits | Not Direct | N.b. | [140] | Expression of an inhibitory (dominant negative) form of the SNARE NtSyp121 inhibits ABA-induced stomatal closure. This finding suggests that SNARE proteins play positive regulatory role in ABA-mediated stomatal closure. | IE |  |
|  | OST1 | CaIM  (Ca^2+^ channels) | Promotes | Not direct | A.t. | [53] | *ost1* mutants are insensitive to ABA-activation of Ca^2+^ channels but plants overexpressing OST1 are hypersensitive to ABA-activation of Ca^2+^ channels. It is unknown whether OST1 interacts with Ca^2+^ channels.  This evidence is ABA specific; there are no studies that investigate this interaction in CO_2_ signaling. | IE |  |
|  | PA | ABI2 | Inhibits | Not direct | A.t. | [141] | Ca^2+^ application to cellular lysates at physiological concentrations inhibits PP2C phosphatase activity. A possible pathway for this inhibition is via PA inhibiting ABI2, which is supported by computational analysis.  There are no studies of PA’s involvement under CO_2_ signaling. | IE |  |
|  | PA | ABI1 | Inhibits | Direct | A.t. | [142, 143] | PA interacts with ABI1, which in turn decreases the phosphatase activity of ABI1. PA also regulates ABI1 by sequestering it to the plasma membrane.  PLDα and abi1 mutant lose ABA-induced closure  There are no studies of PA being involved in CO_2_ signaling. | IE |  |
|  | PA | SPHK1 and SPHK2 (SPHK1/2) | Promotes | Direct | A.t. | [110] | PA interacts with SPHK1 and SPHK2. PA promotes the binding of substrate to SPHKs that in turn accelerates the activity of SPHKs.  There are no studies of these signaling elements’ involvement in the CO_2_ response. | IE |  |
|  | PA | RBOH | Promotes | Direct | A.t. | [144] | PA binds to RbohD/F. Binding of PA activates NADPH oxidase activity of RBOH.  The *rbohD* mutant expressing non-PA-binding RbohD was compromised in ABA-mediated ROS production and stomatal closure.  There are no studies of PA’s involvement in the CO_2_ response. | IE |  |
|  | PC | PA | Promotes | Direct | A.t. | [122] | PC is a substrate for PA production by both PLDα and PLDδ.  There are no studies of their involvement in the CO_2_ response. | IE |  |
|  | PEPC | Malate | Promotes | Not direct | V.f | [105] | PEPC was phosphorylated in vivo when ‘‘isolated’’ guard cells were treated with fusicoccin (FC), which stimulates stomatal opening. The phosphorylation was positively correlated with guard cell malate content and with stomatal aperture size.  There are no studies of the role of PEPC and malate in high CO­_2_ induced stomatal closure. | IE |  |
|  | PI3P5K | PtdIns(3,5)P2 | Promotes | Direct | A.t. | [90] | The enzyme PI35PK produces PtdIns(3,5)P2.  There are no studies of this being involved in high CO_2_ induced closure. | IE |  |
|  | PLC | PtdIns(4,5)P2 (PI4P5P2) | Promotes | Direct | A.t. | [145] | PI45P_2_ (PtdIns(4,5)P2) can be cleaved into inositol 1,4,5-trisphosphate (IP_3_) and diacylglycerol by phospholipase C. There are no studies of PLC’s involvement in high CO_2_ induced closure. | IE |  |
|  | PLC | NO-mediated stomatal closure | Promotes | Not direct | V.f. | [71] | By employing a pharmacological approach, the authors showed that addition of U73122 (PLC inhibitor) inhibits NO induced stomatal closure. This finding suggests that PLC activity is required for NO-mediated stomatal closure.  There are no studies of PLC’s involvement in high CO_2_ induced closure. | IE |  |
|  | PLC | NO-mediated PA production | Promotes | Not direct | V.f. | [71] | PLC hydrolyses PtdIns(4,5)P_2_ into IP_3_ and DAG. Subsequently, DAG can be phosphorylated to PA by DAG kinase (DAGK). PA production increases in response to NO treatment in Vicia. Addition of PLC inhibitor (U73122) causes reduced production of PA in response to NO. This finding suggests that PLC is involved NO-induced PA production.  However, there are no studies of PLC’s involvement under CO_2_ signaling. | IE |  |
|  | PLDα | PA | Promotes | Direct | A.t. | [143] | PA is a product of PLDα.  There are no studies of their involvement in high CO_2_ induced closure. | IE |  |
|  | PLDδ | PA | Promotes | Direct | A.t. | [146] | PA is a product of PLDδ.  There are no studies of their involvement in high CO_2_ induced closure. | IE |  |
|  | PLDδ | NO induced stomatal closure | Promotes | Not direct | A.t. | [147] | PLDδ knockout disrupts NO induced closure. One can assume that NO promotes PLDδ.  There are no studies of PLDδ’s involvement under CO_2_ signaling. | IE |  |
|  | PLDδ | ROS  induced stomatal closure | Promotes | Not direct | A.t. | [131, 147] | PLDδ knockout disrupts ROS induced closure. Taken together with the effect of ROS on GAPC1/2's interaction with PLDδ.  There are no studies of PLDδ’s involvement under CO_2_ signaling. | IE |  |
|  | PP2C.D6/9 | H^+^-ATPase dephosphorylation | Promotes | Direct | A.t. | [20, 148] | Transient expression experiments using Arabidopsis mesophyll cell protoplasts revealed that all PP2C.D isoforms dephosphorylate the endogenous PM H^+^-ATPase; the *pp2c.d6/9* double mutant displayed wider stomatal apertures and greater PM H+-ATPase phosphorylation in response to blue light, but delayed dephosphorylation of PM H^+^-ATPase in guard cells.  Moreover, PP2C D6 and D9 were required for the dephosphorylation and promoted stomatal closure upon the light–dark transition.  There is no investigation of PP2C.D’s role in high CO_2_ induced stomatal closure. | IE |  |
|  | PP2CA | SLAC1 | Inhibits | Direct | A.t., X.l. | [149] | PP2CA means protein phosphatase 2C (also known as AHG3 (ABA Hypersensitive to Germination3). PP2CA physically interacts with SLAC1. Upon coexpression in *Xenopus laevis* oocytes, PP2CA inhibits the activity of SLAC1. A PP2CA phosphatase-dead mutant does not inhibit SLAC1 activity.  It’s possible for PP2CA to be involved in high CO_2_ induced closure like the other PP2Cs, but so far no studies have investigated it. | IE |  |
|  | PP2CA | OST1 | Inhibits | Direct | A.t. | [149] | PP2CA physically interacts with OST1. Upon physical interaction, PP2CA forms a complex with OST1 that in turn blocks the activity of OST1.  No studies have investigated PP2CA’s involvement in high CO_2_ induced closure. | IE |  |
|  | PP2CA | Stomatal closure | Inhibits | Not direct | A.t. | [150] | Overexpression of PP2CA causes ABA insensitivity in ABA induced stomatal closure whereas loss-of-function mutants are hypersensitive in ABA-mediated stomatal closure.  The evidence ABA specific; there are no studies that investigate its involvement in CO_2_ signaling. | IE |  |
|  | PP2CA | CPK6-mediated activation of SLAC1 activity | Inhibits | Direct | A.t.,  X.l. | [58] | PP2CA inhibits CPK6-mediated activation of SLAC1 activity in oocytes. PP2CA physically interacts with SLAC1.  There are no studies that investigate PP2CA being involved in CO_2_ signaling. | IE |  |
|  | PtdIns(3,5)P2 | V-PPase | Promotes | Direct | V.r. | [90] | PtdIns(3,5)P_2_ binds to the V-PPase (vacuolar pyrophosphatase) and activates it.  So far, no studies have investigated the involvement of PtdIns(3,5)P_2_ under high CO_2_ signaling. | IE |  |
|  | PtdIns(3,5)P2 | V-PPase | Promotes | Direct | A.t.,  V.f. | [90] | The functional significance of the interaction between PtdIns(3,5)P_2_ and V-PPase not determined yet. The authors speculate that upon interaction PtdIns(3,5)P_2_ may activate the V-PPase.  So far, no studies have investigated PtdIns(3,5)P_2_’s involvement under high CO_2_ signaling. | IE |  |
|  | PtdIns(3,5)P2 | ABA-induced stomatal closure | Promotes | Not direct | A.t.  V.f. | [90] | PtdIns3P 5-kinase (PI3P5K) is the enzyme responsible for production of PtdIns(3,5)P_2_ from phosphatidylinositol 3-phosphate.  Pharmacological inhibition of PtdIns(3,5)P_2_ biosynthesis causes delayed stomatal closure in response to ABA. In addition, loss-of-function mutants of PI3P5Ks show delayed ABA-induced stomatal closure.  There are no studies of PtdIns(3,5)P2’s involvement under CO_2_ signaling. | IE |  |
|  | PtdIns(4,5)P2 | DAG | Promotes | Direct |  |  | PLC (enzyme) uses substrate PtdIns(4,5)P_2_ for production of DAG.  There are no studies that investigate its involvement in CO_2_ signaling. | IE |  |
|  | PtdIns(4,5)P2 | InsP3 | Promotes | Direct |  |  | PLC (enzyme) uses substrate PtdIns(4,5)P2 for production of InsP3.  There are no studies that investigate its involvement in CO_2_ signaling. | IE |  |
|  | PtdInsP3 | ABA induced ROS production | Promotes | Not direct | V.f.  C.c. | [151-153] | PI3K inhibitors (which deplete PtdInsP3) inhibit the increase of cytosolic calcium and production of ROS in response to ABA in guard cells.  In plants, PtdInsP3 direct regulation of NADPH oxidases (AtrbohD/F) has not been studied. In neutrophils, the NADPH oxidase complex, which consists of many components, is responsible for ROS generation, and is activated by the binding of PtdInsP3to one of the components.  There are no studies of PtdInsP3’s involvement in CO_2_ signaling | IE |  |
|  | PtdInsP3 and PtdInsP4 | Actin reorganization | Promotes | Not direct | C.c. | [152] | (PtdInsP3: phosphatidylinositol 3-phosphate)  (PtdInsP4: phosphatidylinositol 4-phosphate)  LY29402 (inhibitor of PtdInsP3 biosynthesis) and wortmanin (inhibitor of PtdInsP4 biosynthesis) inhibit ABA-induced actin reorganization in guard cells. PtdInsP3 and PtdInsP4 have been implicated as negative regulators of actin binding proteins (ABPs). It is unknown whether InsP3 and InsP4 bind to actin.  There are no studies that investigate the involvement of actin reorganization in CO_2_ signaling. | IE |  |
|  | PtdInsP4 | PtdIns(4,5)P2 | Promotes | Direct |  | [145] | Precursor-product relationship.  There are no studies that investigate its involvement in CO_2_ signaling. | IE |  |
|  | RCARs | ROS | Promote | Not direct | A.t. | [16] | ROS production does not happen in nced mutants, which are ABA deficient. | IE |  |
|  | RCARs | ABI1/ABI2/HAB1 | Inhibits | Direct | A.t. | [30, 102, 104] | Upon ABA binding, RCARs interact with ABI1/ABI2/HAB1 that in turn causes inhibition of their phosphatase activity. | IE |  |
|  | RCARs | PP2CA | Inhibits | Direct | A.t. | [154] | In an in vitro study, it has been shown that soluble ABA receptors RCARs (PYR1, PYL1, PYL2, PYL4, PYL5, PYL6, PYL8) inhibit the phosphatase activity of PP2CA in the presence of ABA. | IE |  |
|  | RCN1 | ABA induced ROS production | Promotes | Not direct | A.t. | [155, 156] | RCN1 is required for full ABA-mediated stomatal closure and ROS production.  There are no studies of RCN’s involvement under CO_2_ signaling. | IE |  |
|  | ROP11 | ABI2 | Promotes | Direct | A.t. | [133] | ROP11 physically interacts with ABI2. The physical interaction between ROP11 and ABI2 promotes phosphatase activity of ABI2, which in turn inhibits OST1 kinase activity.  rop11/arac10 mutants were hypersensitive to ABA.  The interaction part of the evidence may be generic; but there are no studies that investigate its involvement in CO_2_ signaling. | IE |  |
|  | ROP11 | ABI1 | Promotes | Direct | A.t. | [157, 158] | ROP11 physically interacts with ABI1. ROP11 protects the phosphatase activity of ABI1 by interfering with the inhibitory effect of the ABA receptor RCAR1.  There are no studies that investigate its involvement in CO_2_ signaling. | IE |  |
|  | ROS (H_2_O_2_) | HPCA1 | Promotes | Direct | A.t. | [159] | HPCA1 (*hydrogen-peroxide-induced Ca^2+^* *increases*) is activated by H_2_O_2_ via covalent modification of extracellular cysteine residues, which leads to autophosphorylation of HPCA1. HPCA1 mediates H_2_O_2_-induced activation of Ca^2+^ channels in guard cells and is required for stomatal closure. There are no studies that investigate the role of HPCA1 in CO_2_ signaling. | IE |  |
|  | ROS (H_2_O_2_) | Microtubule Depolymerization | Promotes | Not Direct | A.t.,  V.f. | [113] | Application of ROS (H_2_O_2_) causes reduction of resolved microtubule structures.  There are no studies that investigate the involvement of microtubules in CO_2_ signaling. | IE |  |
|  | S1P | Stomatal closure | Promotes | Not direct | A.t., C.c | [107, 160] | Application of S1P induces stomatal closure.  There are no studies of S1P’s involvement in CO_2_ signaling. | IE |  |
|  | S1P | ABA-induced Ca^2+^ increase | Promotes | Not direct | C.c | [160] | S1P causes an increase in Ca^2+^ in response to ABA. As both S1P and Ca^2+^_c_ are part of the strongly connected component of the network, there are paths between them in both directions.  There are no studies of S1P’s involvement in CO_2_ signaling. | IE |  |
|  | S1P/PhytoS1P | S1P/PhytoS1P | Inhibits | Not direct | A.t., pea | [64, 96, 107] | Time course data indicate that in response to ABA, S1P accumulation in guard cells increases quickly and then decreases gradually, subsequently stabilizing above the resting level. This biological phenomenon has been implicated as negative feedback regulation of S1P/PhytoS1P.  There are no studies that investigate its involvement in CO_2_ signaling. | IE |  |
|  | SCAB1 | Actin reorganization | Promotes | Direct | A.t. | [161] | SCAB1 (stomatal closure related actin binding protein1) binds actin filaments. *scab1* mutant shows delayed stomatal closure-associated with slower actin reorganization in response to ABA.  *scab1* mutant shows reduced stomatal movements in response to ABA, ROS and CaCl_2_.  There are no studies that investigate its involvement in CO_2_ signaling. | IE |  |
|  | Sph | S1P/phytoS1P | Promotes | Direct | A.t. | [107] | Sph is a substrate needed for S1P production.  There are no studies that investigate S1P’s involvement in CO_2_ signaling. | IE |  |
|  | SPHK1 and SPHK2 | ABA-induced stomatal closure | Promotes | Not direct | A.t. | [108, 109] | *sphk1* and *sphk2* mutants are deficient in ABA-mediated stomatal closure.  There are no studies of SPHK1/2’s involvement in CO_2_ signaling. | IE |  |
|  | SPHK1/2 | S1P  /phytoS1P | Promotes | Direct | A.t. | [110, 162] | SPHK1 and SPHK2 (SPHK1/2) enzymes are responsible for S1P production.  There are no studies that investigate their involvement in CO_2_ signaling. | IE |  |
|  | SPHK1/2 | ABA activation of PLDα | Promotes | Not direct | A.t. | [110] | ABA activation of PLDα1 is attenuated in *sphk* mutants, as is PA production.  There are no studies of for SPHK1/2’s involvement in CO_2_ signaling. | IE |  |
|  | AGB1 | AGG1 | Binds | Direct | A.t. | [163] | GPA1, AGB1 and AGG1 form a heterotrimer. GPA1 is a known regulator in ABA induced closure. AGB1 physically interacts with AGG1 in yeast (Y2H) and in plants (BiFC). AGB1 and AGG1 function as a heterodimer. There are no studies of the role of AGB1 and AGG1 in high CO_2_ induced closure. | IC |  |
|  | AtABCB14 | Stomatal closure | Inhibits | Not direct | A.t. | [164] | The ABC transporter AtABCB14, identified as a malate uptake transporter in the guard cell plasma membrane, functions as a negative regulator of CO_2_-induced stomatal closure.  Stomatal closure induced by high CO_2_ levels was accelerated in plants lacking AtABCB14.  There is not enough information on the regulation target of AtABCB14 to include it in the network model. | IC |  |
|  | BIG1 | CO_2_-induced stomatal closure | promotes | Not direct | A.t. | [165] | BIG mutants are compromised in elevated CO_2_-induced stomatal closure and bicarbonate activation of S-type anion channel currents.  BIG1 is not included in the network, because it isn’t known to connect to any nodes in the network. | IC |  |
|  | GCA2 | cytosolic Ca^2+^ | Promotes | Not direct | A.t. | [12] | The mutant of growth controlled by abscisic acid (*gca2*) shows impairment in CO_2_ modulation of the cytosolic Ca^2+^ transient rate. This interaction is not included in the model because there is no known regulator or target of GCA2. | IC |  |
|  | GCA2 | Stomatal closure | Promotes | Not direct | A.t. | [12] | The mutant of growth controlled by abscisic acid (*gca2*) shows strong impairment in high CO_2_-induced stomatal closing. This interaction is not included in the model because there is no known regulator or target of GCA2. | IC |  |
|  | GPA1 | AGB1 | Binds | Direct | A.t. | [166] | GPA1 interacts with AGB1, which facilitates the formation of the heterotrimeric G-protein complex. GPA1 is a known regulator in ABA induced closure.  The interaction is generic; however, AGB and AGG don’t regulate any node of the network, so we judge this evidence as “IC”. | IC |  |
|  | High CO_2_ | TPK1 | Promotes | Not direct | A.t. | [167] | During ABA- and CO_2_-mediated closure, TPK1 is phosphorylated and activated by the KIN7 receptor-like protein kinase (RLK), which co-expresses in the tonoplast and plasma membrane.  This interaction is not included in the model, because there is no known target/successor that TPK1 regulates. | IC |  |
|  | High CO_2_ | apoplastic malate concentration | Promotes | Not direct | V.f | [168] | The apoplastic malate concentration rises in response to high CO_2_, which may affect the sensitivity of anion channels in guard cells.  The connections of apoplastic malate concentration to other nodes in the network are unclear, thus it is not included in the model. | IC |  |
|  | RCARs | CO_2_-induced stomatal closure | Promotes | Not direct | A.t. | [169] | PYL4 and PYL5 are found essential in the responses to CO_2_. | IC |  |
|  | KIN7 | TPK1 | Promotes | Direct | A.t. | [167] | During ABA- and CO_2_-mediated closure, TPK1 is phosphorylated and activated by the KIN7 receptor-like protein kinase (RLK), which is co-expressed with it in the tonoplast and plasma membrane.  This interaction is not included in the model, because there is no known target/successor that TPK1 regulates. | IC |  |

**Table S1B. list of known CO_2_-related signaling elements that are not included in our network.**

Here we highlight the nodes with evidence of function under high CO2 induced stomatal closure, yet with insufficient connections to be included in our network model (i.e. signaling elements in the IC category in Table S1A).

| **Signaling element** | **Role/experiment** | **Ref.** | **Comment** |
| --- | --- | --- | --- |
| Apoplastic malate concentration | The apoplastic malate concentration rises in response to high CO_2_; providing external malate to guard cells causes stomatal closure. | [168] | Connection to other signaling elements in the high CO_2_ network is not known. |
| AtABCB14 | The ABC transporter AtABCB14, identified as a malate uptake transporter in the guard cell PM, functions as a negative regulator of CO_2_-induced stomatal closure.  Stomatal closure induced by high CO_2_ levels was accelerated in plants lacking AtABCB14 | [164] | The point of connection to the high CO_2_ network is not known. |
| BIG1 | *big1* mutants are compromised in elevated CO_2_-induced stomatal closure and bicarbonate activation of S-type anion channel currents, but show normal CO_2_ inhibition of opening. | [165] | The point of connection to the high CO_2_ network is not known. |
| GCA2 | The mutant *growth controlled by abscisic acid* (*gca2*) shows impairment in modulation of the cytosolic Ca^2+^ transient rate by CO_2_ concentration and strong impairment in high CO_2_-induced stomatal closure. | [12] | The gene identity of *gca2* remains unknown; the point of connection to the network is not known. |
| KIN7 | The *tpk1*and *kin7* mutants have reduced stomatal closure in ABA and almost no closure in CO_2_. During ABA- and CO_2_-mediated closure, TPK1 is phosphorylated and activated by the KIN7 receptor-like protein kinase (RLK), which co-expresses in the tonoplast and plasma membrane. | [167] | The point of connection to the high CO_2_ network is not known. |
| RCARs | PYL4 and PYL5 are found essential in the responses to CO_2_. | [169] | The point of connection to the high CO_2_ network is not known. |
| TPK1 | ABA causes phosphorylation of TPK1; *tpk1*and *kin7* mutants have reduced stomatal closure in ABA and almost no closure in high CO_2_. | [167] | The point of connection to the high CO_2_ network is not known. |

**References**

1. Zhang, J., et al., *Identification of SLAC1 anion channel residues required for CO2/bicarbonate sensing and regulation of stomatal movements.* Proc Natl Acad Sci U S A, 2018. **115**(44): p. 11129-11137.

2. Wang, C., et al., *Reconstitution of CO2 Regulation of SLAC1 Anion Channel and Function of CO2-Permeable PIP2;1 Aquaporin as CARBONIC ANHYDRASE4 Interactor.* Plant Cell, 2016. **28**(2): p. 568-82.

3. Webb, A.A. and A.M. Hetherington, *Convergence of the abscisic acid, CO2, and extracellular calcium signal transduction pathways in stomatal guard cells.* Plant Physiol, 1997. **114**(4): p. 1557-60.

4. Merilo, E., et al., *PYR/RCAR receptors contribute to ozone-, reduced air humidity-, darkness-, and CO2-induced stomatal regulation.* Plant Physiol, 2013. **162**(3): p. 1652-68.

5. Geiger, D., et al., *Activity of guard cell anion channel SLAC1 is controlled by drought-stress signaling kinase-phosphatase pair.* Proc Natl Acad Sci U S A, 2009. **106**(50): p. 21425-30.

6. Geiger, D., et al., *Guard cell anion channel SLAC1 is regulated by CDPK protein kinases with distinct Ca2+ affinities.* Proc Natl Acad Sci U S A, 2010. **107**(17): p. 8023-8.

7. Hu, H., et al., *Carbonic anhydrases are upstream regulators of CO2-controlled stomatal movements in guard cells.* Nat Cell Biol, 2010. **12**(1): p. 87-93; sup pp 1-18.

8. Xue, S., et al., *Central functions of bicarbonate in S-type anion channel activation and OST1 protein kinase in CO2 signal transduction in guard cell.* EMBO J, 2011. **30**(8): p. 1645-58.

9. Tian, W., et al., *A molecular pathway for CO2 response in Arabidopsis guard cells.* Nature Communications, 2015. **6**(1): p. 6057.

10. Tõldsepp, K., et al., *Mitogen-activated protein kinases MPK4 and MPK12 are key components mediating CO.* Plant J, 2018. **96**(5): p. 1018-1035.

11. Schulze, S., et al., *A role for calcium-dependent protein kinases in differential CO.* New Phytol, 2021. **229**(5): p. 2765-2779.

12. Young, J.J., et al., *CO(2) signaling in guard cells: calcium sensitivity response modulation, a Ca(2+)-independent phase, and CO(2) insensitivity of the gca2 mutant.* Proc Natl Acad Sci U S A, 2006. **103**(19): p. 7506-11.

13. Sun, P., et al., *Countering elevated CO2 induced Fe and Zn reduction in Arabidopsis seeds.* New Phytologist, 2022. **235**(5): p. 1796-1806.

14. Takahashi, Y., et al., *Stomatal CO2/bicarbonate sensor consists of two interacting protein kinases, Raf-like HT1 and non-kinase-activity requiring MPK12/MPK4.* Science Advances, 2022. **8**(49): p. eabq6161.

15. Yeh, C.-Y., et al., *MPK12 in stomatal CO2 signaling: function beyond its kinase activity.* New Phytologist, 2023. **239**(1).

16. Chater, C., et al., *Elevated CO2-Induced Responses in Stomata Require ABA and ABA Signaling.* Curr Biol, 2015. **25**(20): p. 2709-16.

17. Webb, A.A.R., et al., *Carbon dioxide induces increases in guard cell cytosolic free calcium.* The Plant Journal, 1996. **9**(3): p. 297-304.

18. Brearley, J., M.A. Venis, and M.R. Blatt, *The effect of elevated CO2 concentrations on K+ and anion channels of Vicia faba L. guard cells.* Planta, 1997. **203**(2): p. 145-154.

19. Shi, K., et al., *Guard cell hydrogen peroxide and nitric oxide mediate elevated CO2 -induced stomatal movement in tomato.* New Phytol, 2015. **208**(2): p. 342-53.

20. Ando, E., et al., *Elevated CO2 induces rapid dephosphorylation of plasma membrane H+-ATPase in guard cells.* New Phytologist, 2022. **236**(6): p. 2061-2074.

21. Hõrak, H., et al., *A Dominant Mutation in the HT1 Kinase Uncovers Roles of MAP Kinases and GHR1 in CO2-Induced Stomatal Closure.* Plant Cell, 2016. **28**(10): p. 2493-2509.

22. Hashimoto, M., et al., *Arabidopsis HT1 kinase controls stomatal movements in response to CO2.* Nat Cell Biol, 2006. **8**(4): p. 391-7.

23. Hashimoto-Sugimoto, M., et al., *Dominant and recessive mutations in the Raf-like kinase HT1 gene completely disrupt stomatal responses to CO2 in Arabidopsis.* J Exp Bot, 2016. **67**(11): p. 3251-61.

24. Hiyama, A., et al., *Blue light and CO2 signals converge to regulate light-induced stomatal opening.* Nat Commun, 2017. **8**(1): p. 1284.

25. Jakobson, L., et al., *Natural Variation in Arabidopsis Cvi-0 Accession Reveals an Important Role of MPK12 in Guard Cell CO2 Signaling.* PLoS Biol, 2016. **14**(12): p. e2000322.

26. Jalakas, P., et al., *Combined action of guard cell plasma membrane rapid- and slow-type anion channels in stomatal regulation.* Plant Physiol, 2021. **187**(4): p. 2126-2133.

27. Meyer, S., et al., *AtALMT12 represents an R-type anion channel required for stomatal movement in Arabidopsis guard cells.* Plant J, 2010. **63**(6): p. 1054-62.

28. Negi, J., et al., *CO2 regulator SLAC1 and its homologues are essential for anion homeostasis in plant cells.* Nature, 2008. **452**(7186): p. 483-6.

29. Joudoi, T., et al., *Nitrated cyclic GMP modulates guard cell signaling in Arabidopsis.* Plant Cell, 2013. **25**(2): p. 558-71.

30. Nishimura, N., et al., *PYR/PYL/RCAR family members are major in-vivo ABI1 protein phosphatase 2C-interacting proteins in Arabidopsis.* Plant J, 2010. **61**(2): p. 290-9.

31. Umezawa, T., et al., *Type 2C protein phosphatases directly regulate abscisic acid-activated protein kinases in Arabidopsis.* Proc Natl Acad Sci U S A, 2009. **106**(41): p. 17588-93.

32. Vlad, F., et al., *Protein phosphatases 2C regulate the activation of the Snf1-related kinase OST1 by abscisic acid in Arabidopsis.* Plant Cell, 2009. **21**(10): p. 3170-84.

33. Levchenko, V., et al., *Cytosolic abscisic acid activates guard cell anion channels without preceding Ca2+ signals.* Proc Natl Acad Sci U S A, 2005. **102**(11): p. 4203-8.

34. Nagy, S.K., et al., *Activation of AtMPK9 through autophosphorylation that makes it independent of the canonical MAPK cascades.* Biochem J, 2015. **467**(1): p. 167-75.

35. Sanders, D., et al., *Calcium at the crossroads of signaling.* Plant Cell, 2002. **14 Suppl**: p. S401-17.

36. Ward, J.M. and J.I. Schroeder, *Calcium-Activated K+ Channels and Calcium-Induced Calcium Release by Slow Vacuolar Ion Channels in Guard Cell Vacuoles Implicated in the Control of Stomatal Closure.* Plant Cell, 1994. **6**(5): p. 669-683.

37. Kinoshita, T., M. Nishimura, and K. Shimazaki, *Cytosolic Concentration of Ca2+ Regulates the Plasma Membrane H+-ATPase in Guard Cells of Fava Bean.* Plant Cell, 1995. **7**(8): p. 1333-1342.

38. Pei, Z.M., et al., *A transient outward-rectifying K+ channel current down-regulated by cytosolic Ca2+ in Arabidopsis thaliana guard cells.* Proc Natl Acad Sci U S A, 1998. **95**(11): p. 6548-53.

39. Scherzer, S., et al., *Multiple calcium-dependent kinases modulate ABA-activated guard cell anion channels.* Mol Plant, 2012. **5**(6): p. 1409-12.

40. Harmon, A.C., C. Putnam-Evans, and M.J. Cormier, *A Calcium-Dependent but Calmodulin-Independent Protein Kinase from Soybean 1.* Plant Physiology, 1987. **83**(4): p. 830-837.

41. Harper, J.F., et al., *A Calcium-Dependent Protein Kinase with a Regulatory Domain Similar to Calmodulin.* Science, 1991. **252**(5008): p. 951-954.

42. Zhang, T., S. Chen, and A.C. Harmon, *Protein phosphorylation in stomatal movement.* Plant Signaling & Behavior, 2014. **9**(11): p. e972845.

43. Gilroy, S., N.D. Read, and A.J. Trewavas, *Elevation of cytoplasmic calcium by caged calcium or caged inositol triphosphate initiates stomatal closure.* Nature, 1990. **346**(6286): p. 769-71.

44. Chen, Z.H., et al., *Dynamic regulation of guard cell anion channels by cytosolic free Ca2+ concentration and protein phosphorylation.* Plant J, 2010. **61**(5): p. 816-25.

45. Siegel, R.S., et al., *Calcium elevation-dependent and attenuated resting calcium-dependent abscisic acid induction of stomatal closure and abscisic acid-induced enhancement of calcium sensitivities of S-type anion and inward-rectifying K channels in Arabidopsis guard cells.* Plant J, 2009. **59**(2): p. 207-20.

46. Vahisalu, T., et al., *SLAC1 is required for plant guard cell S-type anion channel function in stomatal signalling.* Nature, 2008. **452**(7186): p. 487-91.

47. Mori, I.C., et al., *CDPKs CPK6 and CPK3 function in ABA regulation of guard cell S-type anion- and Ca(2+)-permeable channels and stomatal closure.* PLoS Biol, 2006. **4**(10): p. e327.

48. Guse, A.H., *Cyclic ADP-ribose: a novel Ca2+-mobilising second messenger.* Cell Signal, 1999. **11**(5): p. 309-16.

49. Leckie, C.P., et al., *Abscisic acid-induced stomatal closure mediated by cyclic ADP-ribose.* Proc Natl Acad Sci U S A, 1998. **95**(26): p. 15837-42.

50. Schroeder, J.I. and S. Hagiwara, *Repetitive increases in cytosolic Ca2+ of guard cells by abscisic acid activation of nonselective Ca2+ permeable channels.* Proc Natl Acad Sci U S A, 1990. **87**(23): p. 9305-9.

51. Gilroy, S., et al., *Role of Calcium in Signal Transduction of Commelina Guard Cells.* Plant Cell, 1991. **3**(4): p. 333-344.

52. Grabov, A. and M.R. Blatt, *Membrane voltage initiates Ca2+ waves and potentiates Ca2+ increases with abscisic acid in stomatal guard cells.* Proc Natl Acad Sci U S A, 1998. **95**(8): p. 4778-83.

53. Acharya, B.R., et al., *Open Stomata 1 (OST1) is limiting in abscisic acid responses of Arabidopsis guard cells.* New Phytol, 2013. **200**(4): p. 1049-63.

54. Staxen, I., et al., *Abscisic acid induces oscillations in guard-cell cytosolic free calcium that involve phosphoinositide-specific phospholipase C.* Proc Natl Acad Sci U S A, 1999. **96**(4): p. 1779-84.

55. Lemtiri-Chlieh, F., et al., *Inositol hexakisphosphate mobilizes an endomembrane store of calcium in guard cells.* Proc Natl Acad Sci U S A, 2003. **100**(17): p. 10091-5.

56. Meimoun, P., et al., *Intracellular Ca2+ stores could participate to abscisic acid-induced depolarization and stomatal closure in Arabidopsis thaliana.* Plant Signal Behav, 2009. **4**(9): p. 830-5.

57. Swatek, K.N., et al., *Multisite phosphorylation of 14-3-3 proteins by calcium-dependent protein kinases.* Biochem J, 2014. **459**(1): p. 15-25.

58. Brandt, B., et al., *Reconstitution of abscisic acid activation of SLAC1 anion channel by CPK6 and OST1 kinases and branched ABI1 PP2C phosphatase action.* Proc Natl Acad Sci U S A, 2012. **109**(26): p. 10593-8.

59. Hosy, E., et al., *The Arabidopsis outward K+ channel GORK is involved in regulation of stomatal movements and plant transpiration.* Proc Natl Acad Sci U S A, 2003. **100**(9): p. 5549-54.

60. Hubbard, K.E., et al., *Abscisic acid and CO2 signalling via calcium sensitivity priming in guard cells, new CDPK mutant phenotypes and a method for improved resolution of stomatal stimulus-response analyses.* Ann Bot, 2012. **109**(1): p. 5-17.

61. Sierla, M., et al., *The Receptor-like Pseudokinase GHR1 Is Required for Stomatal Closure.* Plant Cell, 2018. **30**(11): p. 2813-2837.

62. Hua, D., et al., *A plasma membrane receptor kinase, GHR1, mediates abscisic acid- and hydrogen peroxide-regulated stomatal movement in Arabidopsis.* Plant Cell, 2012. **24**(6): p. 2546-61.

63. Mulaudzi, T., et al., *Identification of a novel Arabidopsis thaliana nitric oxide-binding molecule with guanylate cyclase activity in vitro.* FEBS Lett, 2011. **585**(17): p. 2693-7.

64. Li, S., S.M. Assmann, and R. Albert, *Predicting essential components of signal transduction networks: a dynamic model of guard cell abscisic acid signaling.* PLoS Biol, 2006. **4**(10): p. e312.

65. Merlot, S., et al., *Constitutive activation of a plasma membrane H+-ATPase prevents abscisic acid-mediated stomatal closure.* EMBO J, 2007. **26**(13): p. 3216-26.

66. Jammes, F., et al., *MAP kinases MPK9 and MPK12 are preferentially expressed in guard cells and positively regulate ROS-mediated ABA signaling.* Proc Natl Acad Sci U S A, 2009. **106**(48): p. 20520-5.

67. Hunt, L., F. Lerner, and M. Ziegler, *NAD - new roles in signalling and gene regulation in plants.* New Phytologist, 2004. **163**(1): p. 31-44.

68. Desikan, R., et al., *A new role for an old enzyme: nitrate reductase-mediated nitric oxide generation is required for abscisic acid-induced stomatal closure in Arabidopsis thaliana.* Proc Natl Acad Sci U S A, 2002. **99**(25): p. 16314-8.

69. Bright, J., et al., *ABA-induced NO generation and stomatal closure in Arabidopsis are dependent on H2O2 synthesis.* Plant J, 2006. **45**(1): p. 113-22.

70. Sokolovski, S. and M.R. Blatt, *Nitric oxide block of outward-rectifying K+ channels indicates direct control by protein nitrosylation in guard cells.* Plant Physiol, 2004. **136**(4): p. 4275-84.

71. Distefano, A.M., et al., *Nitric oxide-induced phosphatidic acid accumulation: a role for phospholipases C and D in stomatal closure.* Plant Cell Environ, 2008. **31**(2): p. 187-94.

72. Garcı́a-Mata, C. and L. Lamattina, *Nitric Oxide Induces Stomatal Closure and Enhances the Adaptive Plant Responses against Drought Stress.* Plant Physiology, 2001. **126**(3): p. 1196-1204.

73. Dubovskaya, L.V., et al., *cGMP-dependent ABA-induced stomatal closure in the ABA-insensitive Arabidopsis mutant abi1-1.* New Phytol, 2011. **191**(1): p. 57-69.

74. Hsu, P.K., et al., *Abscisic acid-independent stomatal CO2 signal transduction pathway and convergence of CO2 and ABA signaling downstream of OST1 kinase.* Proc Natl Acad Sci U S A, 2018. **115**(42): p. E9971-E9980.

75. Imes, D., et al., *Open stomata 1 (OST1) kinase controls R-type anion channel QUAC1 in Arabidopsis guard cells.* Plant J, 2013. **74**(3): p. 372-82.

76. Sasaki, T., et al., *Closing plant stomata requires a homolog of an aluminum-activated malate transporter.* Plant Cell Physiol, 2010. **51**(3): p. 354-65.

77. Sirichandra, C., et al., *Phosphorylation of the Arabidopsis AtrbohF NADPH oxidase by OST1 protein kinase.* FEBS Lett, 2009. **583**(18): p. 2982-6.

78. Ogasawara, Y., et al., *Synergistic activation of the Arabidopsis NADPH oxidase AtrbohD by Ca2+ and phosphorylation.* J Biol Chem, 2008. **283**(14): p. 8885-92.

79. Kimura, S., et al., *Protein phosphorylation is a prerequisite for the Ca2+-dependent activation of Arabidopsis NADPH oxidases and may function as a trigger for the positive feedback regulation of Ca2+ and reactive oxygen species.* Biochim Biophys Acta, 2012. **1823**(2): p. 398-405.

80. Grondin, A., et al., *Aquaporins Contribute to ABA-Triggered Stomatal Closure through OST1-Mediated Phosphorylation.* Plant Cell, 2015. **27**(7): p. 1945-54.

81. Hedrich, R., *Ion channels in plants.* Physiol Rev, 2012. **92**(4): p. 1777-811.

82. Kohler, B., A. Hills, and M.R. Blatt, *Control of guard cell ion channels by hydrogen peroxide and abscisic acid indicates their action through alternate signaling pathways.* Plant Physiol, 2003. **131**(2): p. 385-8.

83. Zhang, X., et al., *Inhibition of blue light-dependent H+ pumping by abscisic acid through hydrogen peroxide-induced dephosphorylation of the plasma membrane H+-ATPase in guard cell protoplasts.* Plant Physiol, 2004. **136**(4): p. 4150-8.

84. Meinhard, M. and E. Grill, *Hydrogen peroxide is a regulator of ABI1, a protein phosphatase 2C from Arabidopsis.* FEBS Lett, 2001. **508**(3): p. 443-6.

85. Sridharamurthy, M., et al., *H2O2 inhibits ABA-signaling protein phosphatase HAB1.* PLoS One, 2014. **9**(12): p. e113643.

86. Meinhard, M., P.L. Rodriguez, and E. Grill, *The sensitivity of ABI2 to hydrogen peroxide links the abscisic acid-response regulator to redox signalling.* Planta, 2002. **214**(5): p. 775-82.

87. Zhang, X., et al., *Hydrogen peroxide is involved in abscisic acid-induced stomatal closure in Vicia faba.* Plant Physiol, 2001. **126**(4): p. 1438-48.

88. Zhang, W., B.W. Jeon, and S.M. Assmann, *Heterotrimeric G-protein regulation of ROS signalling and calcium currents in Arabidopsis guard cells.* J Exp Bot, 2011. **62**(7): p. 2371-9.

89. Geiger, D., et al., *Stomatal closure by fast abscisic acid signaling is mediated by the guard cell anion channel SLAH3 and the receptor RCAR1.* Sci Signal, 2011. **4**(173): p. ra32.

90. Bak, G., et al., *Rapid structural changes and acidification of guard cell vacuoles during stomatal closure require phosphatidylinositol 3,5-bisphosphate.* Plant Cell, 2013. **25**(6): p. 2202-16.

91. Islam, M.M., et al., *Cytosolic alkalization and cytosolic calcium oscillation in Arabidopsis guard cells response to ABA and MeJA.* Plant Cell Physiol, 2010. **51**(10): p. 1721-30.

92. Savchenko, G., et al., *pH regulation in apoplastic and cytoplasmic cell compartments of leaves.* Planta, 2000. **211**(2): p. 246-255.

93. Miedema, H. and S.M. Assmann, *A membrane-delimited effect of internal pH on the K+ outward rectifier of Vicia faba guard cells.* J Membr Biol, 1996. **154**(3): p. 227-37.

94. Luo, H., P. Morsomme, and M. Boutry, *The two major types of plant plasma membrane H+-ATPases show different enzymatic properties and confer differential pH sensitivity of yeast growth.* Plant Physiol, 1999. **119**(2): p. 627-34.

95. Leube, M.P., E. Grill, and N. Amrhein, *ABI1 of Arabidopsis is a protein serine/threonine phosphatase highly regulated by the proton and magnesium ion concentration.* FEBS Lett, 1998. **424**(1-2): p. 100-4.

96. Puli, M.R., et al., *Stomatal closure induced by phytosphingosine-1-phosphate and sphingosine-1-phosphate depends on nitric oxide and pH of guard cells in Pisum sativum.* Planta, 2016. **244**(4): p. 831-41.

97. Suhita, D., et al., *Cytoplasmic alkalization precedes reactive oxygen species production during methyl jasmonate- and abscisic acid-induced stomatal closure.* Plant Physiol, 2004. **134**(4): p. 1536-45.

98. Wang, X.Q., et al., *G protein regulation of ion channels and abscisic acid signaling in Arabidopsis guard cells.* Science, 2001. **292**(5524): p. 2070-2.

99. Gonugunta, V.K., et al., *Nitric oxide production occurs after cytosolic alkalinization during stomatal closure induced by abscisic acid.* Plant Cell Environ, 2008. **31**(11): p. 1717-24.

100. Gary, J.D., et al., *Fab1p is essential for PtdIns(3)P 5-kinase activity and the maintenance of vacuolar size and membrane homeostasis.* J Cell Biol, 1998. **143**(1): p. 65-79.

101. Baars, T.L., et al., *Role of the V-ATPase in regulation of the vacuolar fission-fusion equilibrium.* Mol Biol Cell, 2007. **18**(10): p. 3873-82.

102. Park, S.Y., et al., *Abscisic acid inhibits type 2C protein phosphatases via the PYR/PYL family of START proteins.* Science, 2009. **324**(5930): p. 1068-71.

103. Gonzalez-Guzman, M., et al., *Arabidopsis PYR/PYL/RCAR receptors play a major role in quantitative regulation of stomatal aperture and transcriptional response to abscisic acid.* Plant Cell, 2012. **24**(6): p. 2483-96.

104. Ma, Y., et al., *Regulators of PP2C phosphatase activity function as abscisic acid sensors.* Science, 2009. **324**(5930): p. 1064-8.

105. Du, Z., K. Aghoram, and W.H. Outlaw, Jr., *In vivo phosphorylation of phosphoenolpyruvate carboxylase in guard cells of Vicia faba L. is enhanced by fusicoccin and suppressed by abscisic acid.* Arch Biochem Biophys, 1997. **337**(2): p. 345-50.

106. Zhang, S.Q., W.H. Outlaw, and R. Chollet, *Lessened malate inhibition of guard-cell phosphoenolpyruvate carboxylase velocity during stomatal opening.* FEBS Lett, 1994. **352**(1): p. 45-8.

107. Coursol, S., et al., *Sphingolipid signalling in Arabidopsis guard cells involves heterotrimeric G proteins.* Nature, 2003. **423**(6940): p. 651-4.

108. Guo, L., et al., *Connections between sphingosine kinase and phospholipase D in the abscisic acid signaling pathway in Arabidopsis.* J Biol Chem, 2012. **287**(11): p. 8286-96.

109. Worrall, D., et al., *Involvement of sphingosine kinase in plant cell signalling.* Plant J, 2008. **56**(1): p. 64-72.

110. Guo, L., et al., *Phosphatidic acid binds and stimulates Arabidopsis sphingosine kinases.* J Biol Chem, 2011. **286**(15): p. 13336-45.

111. Lemichez, E., et al., *Inactivation of AtRac1 by abscisic acid is essential for stomatal closure.* Genes Dev, 2001. **15**(14): p. 1808-16.

112. Dittrich, P. and K. Raschke, *Malate metabolism in isolated epidermis of Commelina communis L. in relation to stomatal functioning.* Planta, 1977. **134**(1): p. 77-81.

113. Eisinger, W., D. Ehrhardt, and W. Briggs, *Microtubules are essential for guard-cell function in Vicia and Arabidopsis.* Mol Plant, 2012. **5**(3): p. 601-10.

114. Hugouvieux, V., J.M. Kwak, and J.I. Schroeder, *An mRNA cap binding protein, ABH1, modulates early abscisic acid signal transduction in Arabidopsis.* Cell, 2001. **106**(4): p. 477-87.

115. Murata, Y., et al., *Abscisic acid activation of plasma membrane Ca(2+) channels in guard cells requires cytosolic NAD(P)H and is differentially disrupted upstream and downstream of reactive oxygen species production in abi1-1 and abi2-1 protein phosphatase 2C mutants.* Plant Cell, 2001. **13**(11): p. 2513-23.

116. Zhang, W., L.M. Fan, and W.H. Wu, *Osmo-sensitive and stretch-activated calcium-permeable channels in Vicia faba guard cells are regulated by actin dynamics.* Plant Physiol, 2007. **143**(3): p. 1140-51.

117. Jeon, B.W., B.R. Acharya, and S.M. Assmann, *The Arabidopsis heterotrimeric G-protein β subunit, AGB1, is required for guard cell calcium sensing and calcium-induced calcium release.* The Plant Journal, 2019. **99**(2): p. 231-244.

118. Jiang, K., et al., *The ARP2/3 complex mediates guard cell actin reorganization and stomatal movement in Arabidopsis.* Plant Cell, 2012. **24**(5): p. 2031-40.

119. Szymanski, D.B., *Breaking the WAVE complex: the point of Arabidopsis trichomes.* Curr Opin Plant Biol, 2005. **8**(1): p. 103-12.

120. Nakagawa, N., et al., *Degradation of long-chain base 1-phosphate (LCBP) in Arabidopsis: functional characterization of LCBP phosphatase involved in the dehydration stress response.* J Plant Res, 2012. **125**(3): p. 439-49.

121. Kim, Y.M., et al., *Overexpression of Arabidopsis translationally controlled tumor protein gene AtTCTP enhances drought tolerance with rapid ABA-induced stomatal closure.* Mol Cells, 2012. **33**(6): p. 617-26.

122. Pappan, K.L. and X. Wang, *Assaying different types of plant phospholipase D activities in vitro.* Methods Mol Biol, 2013. **1009**: p. 205-17.

123. Otterhag, L., M. Sommarin, and C. Pical, *N-terminal EF-hand-like domain is required for phosphoinositide-specific phospholipase C activity in Arabidopsis thaliana.* FEBS Lett, 2001. **497**(2-3): p. 165-70.

124. Tang, R.J., et al., *Tonoplast calcium sensors CBL2 and CBL3 control plant growth and ion homeostasis through regulating V-ATPase activity in Arabidopsis.* Cell Res, 2012. **22**(12): p. 1650-65.

125. MacRobbie, E.A., *ABA activates multiple Ca2+ fluxes in stomatal guard cells, triggering vacuolar K+(Rb+) release.* Proc Natl Acad Sci U S A, 2000. **97**(22): p. 12361-8.

126. Macrobbie, E.A., *Signalling in guard cells and regulation of ion channel activity.* J Exp Bot, 1997. **48 Spec No**: p. 515-28.

127. Munnik, T., R.F. Irvine, and A. Musgrave, *Phospholipid signalling in plants.* Biochim Biophys Acta, 1998. **1389**(3): p. 222-72.

128. Huang, J., et al., *Carbonic Anhydrases Function in Anther Cell Differentiation Downstream of the Receptor-Like Kinase EMS1.* Plant Cell, 2017. **29**(6): p. 1335-1356.

129. Zheng, Z.L., et al., *Plasma membrane-associated ROP10 small GTPase is a specific negative regulator of abscisic acid responses in Arabidopsis.* Plant Cell, 2002. **14**(11): p. 2787-97.

130. Allen, G.J., et al., *Hypersensitivity of abscisic acid-induced cytosolic calcium increases in the Arabidopsis farnesyltransferase mutant era1-2.* Plant Cell, 2002. **14**(7): p. 1649-62.

131. Guo, L., et al., *Cytosolic glyceraldehyde-3-phosphate dehydrogenases interact with phospholipase Ddelta to transduce hydrogen peroxide signals in the Arabidopsis response to stress.* Plant Cell, 2012. **24**(5): p. 2200-12.

132. Pandey, S. and S.M. Assmann, *The Arabidopsis putative G protein-coupled receptor GCR1 interacts with the G protein alpha subunit GPA1 and regulates abscisic acid signaling.* Plant Cell, 2004. **16**(6): p. 1616-32.

133. Yu, F., et al., *FERONIA receptor kinase pathway suppresses abscisic acid signaling in Arabidopsis by activating ABI2 phosphatase.* Proc Natl Acad Sci U S A, 2012. **109**(36): p. 14693-8.

134. Li, Z. and D. Liu, *ROPGEF1 and ROPGEF4 are functional regulators of ROP11 GTPase in ABA-mediated stomatal closure in Arabidopsis.* FEBS Lett, 2012. **586**(9): p. 1253-8.

135. Zhao, J. and X. Wang, *Arabidopsis phospholipase Dalpha1 interacts with the heterotrimeric G-protein alpha-subunit through a motif analogous to the DRY motif in G-protein-coupled receptors.* J Biol Chem, 2004. **279**(3): p. 1794-800.

136. Boss, W.F. and Y.J. Im, *Phosphoinositide signaling.* Annu Rev Plant Biol, 2012. **63**: p. 409-29.

137. Jiang, Y., et al., *Phosphatidic acid integrates calcium signaling and microtubule dynamics into regulating ABA-induced stomatal closure in Arabidopsis.* Planta, 2014. **239**(3): p. 565-75.

138. Gardner, M.K., M. Zanic, and J. Howard, *Microtubule catastrophe and rescue.* Curr Opin Cell Biol, 2013. **25**(1): p. 14-22.

139. Suh, S.J., et al., *The ATP binding cassette transporter AtMRP5 modulates anion and calcium channel activities in Arabidopsis guard cells.* J Biol Chem, 2007. **282**(3): p. 1916-24.

140. Sokolovski, S., et al., *Functional interaction of the SNARE protein NtSyp121 in Ca2+ channel gating, Ca2+ transients and ABA signalling of stomatal guard cells.* Mol Plant, 2008. **1**(2): p. 347-58.

141. Maheshwari, P., et al., *Model-driven discovery of calcium-related protein-phosphatase inhibition in plant guard cell signaling.* PLoS Comput Biol, 2019. **15**(10): p. e1007429.

142. Mishra, G., et al., *A bifurcating pathway directs abscisic acid effects on stomatal closure and opening in Arabidopsis.* Science, 2006. **312**(5771): p. 264-6.

143. Zhang, W., et al., *Phospholipase D alpha 1-derived phosphatidic acid interacts with ABI1 phosphatase 2C and regulates abscisic acid signaling.* Proc Natl Acad Sci U S A, 2004. **101**(25): p. 9508-13.

144. Zhang, Y., et al., *Phospholipase dalpha1 and phosphatidic acid regulate NADPH oxidase activity and production of reactive oxygen species in ABA-mediated stomatal closure in Arabidopsis.* Plant Cell, 2009. **21**(8): p. 2357-77.

145. Jung, J.Y., et al., *Phosphatidylinositol 3- and 4-phosphate are required for normal stomatal movements.* Plant Cell, 2002. **14**(10): p. 2399-412.

146. Uraji, M., et al., *Cooperative function of PLDdelta and PLDalpha1 in abscisic acid-induced stomatal closure in Arabidopsis.* Plant Physiol, 2012. **159**(1): p. 450-60.

147. Distefano, A.M., et al., *Phospholipase Ddelta is involved in nitric oxide-induced stomatal closure.* Planta, 2012. **236**(6): p. 1899-907.

148. Akiyama, M., et al., *Type 2C protein phosphatase clade D family members dephosphorylate guard cell plasma membrane H+-ATPase.* Plant Physiology, 2021. **188**(4): p. 2228-2240.

149. Lee, S.C., et al., *A protein kinase-phosphatase pair interacts with an ion channel to regulate ABA signaling in plant guard cells.* Proc Natl Acad Sci U S A, 2009. **106**(50): p. 21419-24.

150. Kuhn, J.M., et al., *The protein phosphatase AtPP2CA negatively regulates abscisic acid signal transduction in Arabidopsis, and effects of abh1 on AtPP2CA mRNA.* Plant Physiology, 2006. **140**(1): p. 127-39.

151. Park, K.Y., et al., *A role for phosphatidylinositol 3-phosphate in abscisic acid-induced reactive oxygen species generation in guard cells.* Plant Physiol, 2003. **132**(1): p. 92-8.

152. Choi, Y., et al., *Phosphatidylinositol 3- and 4-phosphate modulate actin filament reorganization in guard cells of day flower.* Plant Cell Environ, 2008. **31**(3): p. 366-77.

153. Ellson, C.D., et al., *PtdIns(3)P regulates the neutrophil oxidase complex by binding to the PX domain of p40(phox).* Nat Cell Biol, 2001. **3**(7): p. 679-82.

154. Antoni, R., et al., *Selective inhibition of clade A phosphatases type 2C by PYR/PYL/RCAR abscisic acid receptors.* Plant Physiol, 2012. **158**(2): p. 970-80.

155. Saito, N., et al., *Roles of RCN1, regulatory A subunit of protein phosphatase 2A, in methyl jasmonate signaling and signal crosstalk between methyl jasmonate and abscisic acid.* Plant Cell Physiol, 2008. **49**(9): p. 1396-401.

156. Kwak, J.M., et al., *Disruption of a guard cell-expressed protein phosphatase 2A regulatory subunit, RCN1, confers abscisic acid insensitivity in Arabidopsis.* Plant Cell, 2002. **14**(11): p. 2849-61.

157. Li, Z., et al., *ROP11 GTPase negatively regulates ABA signaling by protecting ABI1 phosphatase activity from inhibition by the ABA receptor RCAR1/PYL9 in Arabidopsis.* J Integr Plant Biol, 2012. **54**(3): p. 180-8.

158. Li, Z., et al., *ROP11 GTPase is a negative regulator of multiple ABA responses in Arabidopsis.* J Integr Plant Biol, 2012. **54**(3): p. 169-79.

159. Wu, F., et al., *Hydrogen peroxide sensor HPCA1 is an LRR receptor kinase in Arabidopsis.* Nature, 2020. **578**(7796): p. 577-581.

160. Ng, C.K., et al., *Drought-induced guard cell signal transduction involves sphingosine-1-phosphate.* Nature, 2001. **410**(6828): p. 596-9.

161. Zhao, Y., et al., *The plant-specific actin binding protein SCAB1 stabilizes actin filaments and regulates stomatal movement in Arabidopsis.* Plant Cell, 2011. **23**(6): p. 2314-30.

162. Guo, L. and X. Wang, *Crosstalk between Phospholipase D and Sphingosine Kinase in Plant Stress Signaling.* Front Plant Sci, 2012. **3**: p. 51.

163. Chakravorty, D., et al., *An atypical heterotrimeric G-protein gamma-subunit is involved in guard cell K+-channel regulation and morphological development in Arabidopsis thaliana.* Plant J, 2011. **67**(5): p. 840-51.

164. Lee, M., et al., *The ABC transporter AtABCB14 is a malate importer and modulates stomatal response to CO2.* Nat Cell Biol, 2008. **10**(10): p. 1217-23.

165. He, J., et al., *The BIG protein distinguishes the process of CO2 -induced stomatal closure from the inhibition of stomatal opening by CO2.* The New phytologist, 2018. **218**(1): p. 232-241.

166. Gookin, T.E. and S.M. Assmann, *Significant reduction of BiFC non-specific assembly facilitates in planta assessment of heterotrimeric G-protein interactors.* Plant J, 2014. **80**(3): p. 553-67.

167. Isner, J.C., et al., *KIN7 Kinase Regulates the Vacuolar TPK1 K.* Curr Biol, 2018. **28**(3): p. 466-472.e4.

168. Hedrich, R., et al., *Malate-sensitive anion channels enable guard cells to sense changes in the ambient CO2 concentration.* The Plant Journal, 1994. **6**(5): p. 741-748.

169. Dittrich, M., et al., *The role of Arabidopsis ABA receptors from the PYR/PYL/RCAR family in stomatal acclimation and closure signal integration.* Nat Plants, 2019. **5**(9): p. 1002-1011.
