# Supplementary material for: A network-based modeling framework reveals the core signal transduction network underlying high carbon dioxide-induced stomatal closure in guard cells
Source: PLoS Biol. 2024 May 1;22(5):e3002592. doi: 10.1371/journal.pbio.3002592 (PMC11090369; doi:10.1371/journal.pbio.3002592)
Supplement: S2 Table — (DOCX) [file pbio.3002592.s002.docx]

**S2 Table. Description of the 47 nodes and 95 edges of the CO_2_ network**

**Table S2A. The full name of the abbreviated node names in the network, and evidence of each node’s involvement in high CO_2_ induced closure**

The 3^rd^ column is the specific evidence that supports the inclusion each node in the CO_2_ signaling network. A reference indicates experimental evidence confirming the node’s role in CO_2_ signaling. “G” is short for “Generic evidence**”**, indicating there is generic evidence (e.g. a chemical reaction or physical process) for this node to be involved in CO_2_ signaling. “A” is short for “Assumed”, meaning the node is assumed to be involved in CO_2_ signaling, e.g., based on extrapolation of known processes, and the model is less consistent with experimental observations without this node, yet there is no direct experimental evidence or confirmation of this node’s involvement. For example, the participation of ADPRc in the network is assumed because without it, NO cannot form a pathway to regulate closure. More details can be found in S1 Table.

Only evidence specific to CO_2_ signaling is included. For example, NO donor is known to cause stomatal closure, but this evidence is not CO_2_-specific and thus is not included. The CO_2_-specific evidence for NO is the observation that NO is produced under high CO_2_.

To represent signaling elements with different functions, we deploy a multi-node representation for the nodes OST1, MPKs and SLAC1. OST1 is a known essential regulator of high CO_2_ induced closure, but OST1 kinase activity is not elevated during high CO_2_ induced closure [1, 2]. Thus, we create a node pair, “OST1 minimum function” and “OST1 activated”, to represent the minimum function of OST1 necessary for high CO_2_ induced closure (see S1 Text for details), and the elevation of OST1 kinase activity observed in e.g., ABA-induced closure but not observed under high CO_2_, respectively. Similarly, MPKs were found to form a complex with HT1 in the presence of bicarbonate (HCO_3_^–^); this CO_2_-specific process does not depend on the kinase activity of MPKs [3-6]. Nevertheless, MPKs were also found to be necessary in Ca^2+^ induced SLAC1 activity [7]; we assume that this role depends on a CO_2_-independent activity of MPKs, which could potentially be a canonical mitogen activated protein kinase activity. This assumption fills a gap of understanding regarding closure driven by providing ROS, cADPR, 8-nitro-cGMP, or external Ca^2+^ under ambient CO_2_. For example, the experimental observation of external Ca^2+^-induced closure in ambient CO_2_ can only be recapitulated if assuming a CO_2_-independent mechanism of inhibition of HT1 by MPKs or by another Ca^2+^-activated node. We assume that this inhibition depends on the MPK activity. So, we create two nodes, “MPKs-HT1 complex” and “MPK activity”, to reflect these two functions respectively (see main text Figure 2C). We also split SLAC1 into a node pair, wherein “SLAC1 CO_2_” represents the high-CO_2_ specific mechanisms to induce anion flow through the SLAC1 channel, influenced by bicarbonate [8] and CBC1/2 [9], and “SLAC1 generic”, which captures SLAC1 activation by other mechanisms.

| **Node name** | **Full name or explanation** | **CO_2_-specific evidence** |
| --- | --- | --- |
| 8-nitro-cGMP | 8-nitro-cyclic guanosine monophosphate | G |
| ABI1 | ABA (abscisic acid)-insensitive protein phosphatase 1 | [10, 11] |
| ABI2 | ABA (abscisic acid)-insensitive protein phosphatase 2 | [11] |
| ADPRc | ADP (adenosine diphosphate)-ribosyl cyclase | A |
| AnionEM | Anion efflux across the plasma membrane | G |
| Aquaporins | Water channel proteins that form pores in the membrane facilitating water transport, including the plasma membrane intrinsic protein 2;1 (PIP2;1) | G |
| Ca^2+^ATPase | Ca^2+^ ATPases and Ca^2+/^H^+^ antiporters responsible for Ca^2+^ efflux from the cytosol | [12] |
| CAs | Carbonic anhydrase enzymes CA1, CA4 and CA7 | [13-15] |
| Ca^2+^_cyt_ | Cytosolic calcium | [12, 16] |
| cADPR | cyclic ADP-ribose | A |
| CaIM | Ca^2+^ influx across the plasma membrane | [12] |
| CBC1/2 | Protein kinases CBC1/2 (CONVERGENCE OF BLUE LIGHT (BL) AND CO_2_ 1/2) | [5, 9] |
| cGMP | Cyclic guanosine monophosphate | G |
| CIS | Ca^2+^ influx to the cytosol from intracellular stores | G |
| Closure | Stomatal closure | G |
| CO_2_ entry of cell | A conceptual node representing CO_2_ entry into the cell | G |
| CO_2_ porins | CO_2_ porins that allow the CO_2_ molecule to cross the guard cell membrane | G |
| CPKs | Calcium-dependent protein kinases involved in high CO_2_ induced stomatal closure, including CPK3, CPK5, CPK6, CPK11, CPK21, CPK23. | [17] |
| Depolarization | Plasma membrane depolarization | G |
| GHR1 | The leucine-rich repeat receptor-like pseudokinase GUARD CELL HYDROGEN PEROXIDE-RESISTANT1 | [18] |
| GTP | Guanosine 5'-triphosphate | G |
| H^+^ ATPase | H^+^ ATPase at the plasma membrane | [19] |
| H_2_O efflux | water efflux across the plasma membrane | G |
| HAB1 | Hypersensitive to ABA 1 phosphatase | A |
| HCO_3_^-^ | Bicarbonate | [8, 14, 20] |
| High CO_2_ | High carbon dioxide concentration | G |
| HT1 | Arabidopsis kinase high leaf temperature 1 | [4, 21, 22] |
| K^+^ efflux | K^+^ efflux across the plasma membrane | [23] |
| KEV | K^+^ efflux from the vacuole to the cytosol | [23] |
| KOUT | K^+^ efflux through slowly activating outwardly-rectifying K^+^ channels through the plasma membrane | [23] |
| MPKs | Mitogen-activated protein kinases, including MPK4, MPK12, MPK9. | [3, 7, 24] |
| MPKs-HT1 complex | The complex of MPKs and HT1 formed under bicarbonate and independent of MPKs’ kinase activity | [5, 6] |
| MPK activity | MPKs’ activity (including kinase activity) related to stomatal closure but not specific to high CO_2_-induced closure | [5-7, 24] |
| NAD^+^ | Nicotinamide adenine dinucleotide | G |
| NADPH | Nicotinamide adenine dinucleotide phosphate | G |
| NIA1/2 | Nitrate reductase 1/2 | G |
| Nitrite | Nitrite | G |
| NO | Nitric Oxide | [25] |
| NOGC | Nitric Oxide dependent Guanylate Cyclase | A |
| OST1 activated | The activated level of Open Stomata 1 protein kinase, which is observed in ABA induced closure but not in high CO_2_ induced closure | Confirmed not in CO_2_ signaling [1, 2] |
| OST1 minimum function | The minimum function of the Open Stomata1 protein kinase that is necessary to promote closure | [14] |
| QUAC1 | Quickly activating Anion Channel1 (also known as AtALMT12) | [26] |
| RbohD/F | The Respiratory burst oxidase homolog (Rboh) gene family encodes the key enzymatic subunit of the plant NADPH oxidase. In our network model RbohD/F denotes AtRBOH D and F. | [27] |
| RHC1 | Arabidopsis MATE-type transporter RESISTANT TO HIGH CO_2_ | [28] |
| ROS | Reactive oxygen species | [27] |
| SLAC1 CO_2_ | SLOW ANION CHANNEL-ASSOCIATED 1, high CO_2_ specific contribution | [26, 29] |
| SLAC1 generic | The generic (i.e. not specific to CO_2_) contribution to the activation of SLOW ANION CHANNEL-ASSOCIATED 1 | [8] |

**Table S2B. Edge list**

Edges with straightforward reasons, e.g. high CO_2_ → CO_2_ entry of cell, do not have a reference.

| **Start node** | **End node** | **Edge sign** | **Reference** | **Edge type (direct/indirect) and assumption** |
| --- | --- | --- | --- | --- |
| 8-nitro-cGMP | ADPRc | + | [30-32] | Assumed based on the observation that ADPRc antagonists inhibit 8-nitro-cGMP induced stomatal closure |
| ABI1 | OST1 minimum function | - | [33-35] | Direct |
| ABI1 | OST1 activated | - | [33-35] | Direct |
| ABI1 | SLAC1 generic | - | [10, 36, 37] | Indirect |
| ABI1 | SLAC1 CO_2_ | - | [10, 36, 37] | Indirect |
| ABI2 | OST1 minimum function | - | [35] | Direct |
| ABI2 | OST1 activated | - | [35] | Direct |
| ABI2 | GHR1 | - | [38] | Direct |
| ABI2 | SLAC1 generic | - | [10, 36, 37] | Indirect |
| ABI2 | SLAC1 CO_2_ | - | [10, 36, 37] | Indirect |
| ADPRc | cADPR | + |  | Direct (elementary reaction) |
| AnionEM | Depolarization | + |  | Direct physical process |
| AnionEM | H_2_O Efflux | + |  | Direct |
| Aquaporins | H_2_O Efflux | + | [20, 39] | Direct |
| Ca^2+^ATPase | Ca^2+^_cyt_ | - |  | Direct |
| CAs | HCO_3_^-^ | + | [13-15] | Direct |
| CAs | RHC1 | + | [28] | Direct |
| Ca^2+^_cyt_ | MPK activity | + | [4, 7, 24] | Indirect |
| Ca^2+^_cyt_ | Ca^2+^ATPase | + | [40] | Direct |
| Ca^2+^_cyt_ | CPKs | + | [36] | Direct |
| Ca^2+^_cyt_ | QUAC1 | + | [41] | Assumed based on the observation that the *quac1* loss-of-function mutant shows hyposensitivity to Ca^2+^-induced stomatal closure |
| Ca^2+^_cyt_ | H^+^ ATPase | - | [42] | Direct |
| Ca^2+^_cyt_ | KEV | + | [43] | Direct |
| Ca^2+^_cyt_ | Depolarization | + |  | Direct physical process |
| cADPR | CIS | + | [44] | Indirect |
| CaIM | ABI1 | - | [45] | Indirect |
| CaIM | ABI2 | - | [45] | Indirect |
| CaIM | HAB1 | - | [45] | Indirect |
| CaIM | Ca^2+^_cyt_ | + |  | Direct physical process |
| CBC1/2 | SLAC1 CO_2_ | - | [9] | Indirect |
| cGMP | 8-nitro-cGMP | + |  | Direct (elementary reaction) |
| CIS | Ca^2+^_cyt_ | + |  | Direct physical process |
| CO_2_ entry of cell | HCO_3_^-^ | + | [13, 14] | Direct |
| CO_2_ porins | CO_2_ entry of cell | + | [20] | Direct |
| CPKs | CPKs | + | [46] | Direct, assumed autophosphorylation |
| CPKs | SLAC1 generic | + | [36, 37, 47] | Direct |
| Depolarization | KOUT | + |  | Direct physical process |
| GHR1 | CaIM | + | [38] | Assumed based on the observation that *ghr1* loss of function mutants show impaired Ca^2+^ influx |
| GHR1 | CPKs | + | [18] | Direct |
| GHR1 | SLAC1 generic | - | [38] | Direct |
| GHR1 | SLAC1 CO_2_ | - | [38] | Direct |
| GTP | cGMP | + | [48] | Direct |
| H^+^ ATPase | Depolarization | - |  | Direct physical process |
| H_2_O Efflux | Closure |  |  | Direct physical process |
| HAB1 | OST1 minimum function | - | [35] | Direct |
| HAB1 | OST1 activated | - | [35] | Direct |
| HCO_3_^-^ | MPKs-HT1 complex | + | [3-6, 24] | Direct |
| HCO_3_^-^ | RHC1 | + | [28] | Indirect |
| HCO_3_^-^ | SLAC1 CO_2_ | + | [8] | Direct |
| High CO_2_ | CO_2_ entry of cell | + |  | Direct |
| HT1 | CBC1/2 | + | [5] | Direct |
| HT1 | GHR1 | - | [4, 18, 28] | Direct |
| HT1 | OST1 minimum function | - | [4, 28] | Direct |
| HT1 | OST1 activated | - | [4, 28] | Direct |
| K^+^ efflux | Depolarization | - |  | Direct physical process |
| K^+^ efflux | H_2_O Efflux | + |  | Direct physical process |
| K^+^ efflux | K^+^ efflux | + |  | Assumed to ensure that K^+^ efflux does not oscillate |
| KEV | Depolarization | + |  | Direct physical process |
| KEV | K^+^ efflux | + |  | Direct physical process |
| KOUT | K^+^ efflux | + |  | Direct physical process |
| MPKs | MPKs-HT1 complex | + |  | Direct requirement |
| MPKs | MPK activity | + |  | Direct requirement |
| MPKs-HT1 complex | HT1 | - | [3-6, 24] | Direct |
| MPK activity | HT1 | - | [4, 7, 24] | Assumed because MPKs are necessary in Ca^2+^ induced closure, and HT1 must be inhibited in the model to achieve closure |
| MPK activity | MPK activity | + | [49] | Assumed to stabilize the effect of calcium oscillation, for more model consistency with observation |
| MPK activity | SLAC1 generic | + | [7] | Assumed based on the lack of activation of SLAC1 in *mpk9/mpk12* double mutants |
| NAD^+^ | cADPR | + |  | Direct (elementary reaction) |
| NADPH | NO | + | [50] | Direct (elementary reaction) |
| NADPH | ROS | + |  | Direct (elementary reaction) |
| NIA1/2 | NO | + | [50] | Direct (elementary reaction) |
| Nitrite | NO | + | [50] | Direct (elementary reaction) |
| NO | 8-nitro-cGMP | + | [51] | Direct (elementary reaction) |
| NO | NOGC | + | [48] | Direct (elementary reaction) |
| NO | KOUT | - | [52] | Indirect |
| NOGC | cGMP | + | [48] | Direct (elementary reaction) |
| OST1 activated | SLAC1 generic | + | [10] | Direct |
| OST1 minimum function | OST1 activated | + |  | Direct requirement |
| OST1 minimum function | RbohD/F | + | [53] | Direct |
| OST1 minimum function | SLAC1 generic | + | [10, 54] | Direct |
| OST1 minimum function | SLAC1 CO_2_ | + | [10, 54] | Direct |
| OST1 minimum function | QUAC1 | + | [55] | Direct |
| OST1 minimum function | Aquaporins | + | [39] | Direct |
| QUAC1 | AnionEM | + | [56] | Direct physical process |
| RbohD/F | ROS | + |  | Direct (elementary reaction) |
| RHC1 | HT1 | - | [28] | Direct |
| ROS | GHR1 | + | [38] | Indirect |
| ROS | ABI1 | - | [57] | Direct |
| ROS | ABI2 | - | [58] | Direct |
| ROS | HAB1 | - | [59] | Direct |
| ROS | 8-nitro-cGMP | + | [51] | Indirect |
| ROS | NIA1/2 | + | [60] | Assumed based on the observation that ROS fails to induce NO production in the *nia1/nia2* double mutant. |
| ROS | H^+^ATPase | - | [61] | Indirect |
| ROS | KOUT | - | [62] | Indirect |
| SLAC1 CO_2_ | AnionEM | + |  | Direct physical process |
| SLAC1 generic | AnionEM | + |  | Direct physical process |

**References**

1. Hsu, P.K., et al., *Abscisic acid-independent stomatal CO2 signal transduction pathway and convergence of CO2 and ABA signaling downstream of OST1 kinase.* Proc Natl Acad Sci U S A, 2018. **115**(42): p. E9971-E9980.

2. Zhang, L., et al., *FRET kinase sensor development reveals SnRK2/OST1 activation by ABA but not by MeJA and high CO.* Elife, 2020. **9**.

3. Jakobson, L., et al., *Natural Variation in Arabidopsis Cvi-0 Accession Reveals an Important Role of MPK12 in Guard Cell CO2 Signaling.* PLoS Biol, 2016. **14**(12): p. e2000322.

4. Hõrak, H., et al., *A Dominant Mutation in the HT1 Kinase Uncovers Roles of MAP Kinases and GHR1 in CO2-Induced Stomatal Closure.* Plant Cell, 2016. **28**(10): p. 2493-2509.

5. Takahashi, Y., et al., *Stomatal CO2/bicarbonate sensor consists of two interacting protein kinases, Raf-like HT1 and non-kinase-activity requiring MPK12/MPK4.* Science Advances, 2022. **8**(49): p. eabq6161.

6. Yeh, C.-Y., et al., *MPK12 in stomatal CO2 signaling: function beyond its kinase activity.* New Phytologist, 2023. **239**(1).

7. Jammes, F., et al., *MAP kinases MPK9 and MPK12 are preferentially expressed in guard cells and positively regulate ROS-mediated ABA signaling.* Proc Natl Acad Sci U S A, 2009. **106**(48): p. 20520-5.

8. Zhang, J., et al., *Identification of SLAC1 anion channel residues required for CO2/bicarbonate sensing and regulation of stomatal movements.* Proc Natl Acad Sci U S A, 2018. **115**(44): p. 11129-11137.

9. Hayashi, M., et al., *Raf-like kinases CBC1 and CBC2 negatively regulate stomatal opening by negatively regulating plasma membrane H.* Photochem Photobiol Sci, 2020. **19**(1): p. 88-98.

10. Geiger, D., et al., *Activity of guard cell anion channel SLAC1 is controlled by drought-stress signaling kinase-phosphatase pair.* Proc Natl Acad Sci U S A, 2009. **106**(50): p. 21425-30.

11. Webb, A.A. and A.M. Hetherington, *Convergence of the abscisic acid, CO2, and extracellular calcium signal transduction pathways in stomatal guard cells.* Plant Physiol, 1997. **114**(4): p. 1557-60.

12. Young, J.J., et al., *CO(2) signaling in guard cells: calcium sensitivity response modulation, a Ca(2+)-independent phase, and CO(2) insensitivity of the gca2 mutant.* Proc Natl Acad Sci U S A, 2006. **103**(19): p. 7506-11.

13. Hu, H., et al., *Carbonic anhydrases are upstream regulators of CO2-controlled stomatal movements in guard cells.* Nat Cell Biol, 2010. **12**(1): p. 87-93; sup pp 1-18.

14. Xue, S., et al., *Central functions of bicarbonate in S-type anion channel activation and OST1 protein kinase in CO2 signal transduction in guard cell.* EMBO J, 2011. **30**(8): p. 1645-58.

15. Sun, P., et al., *Countering elevated CO2 induced Fe and Zn reduction in Arabidopsis seeds.* New Phytologist, 2022. **235**(5): p. 1796-1806.

16. Hubbard, K.E., et al., *Abscisic acid and CO2 signalling via calcium sensitivity priming in guard cells, new CDPK mutant phenotypes and a method for improved resolution of stomatal stimulus-response analyses.* Ann Bot, 2012. **109**(1): p. 5-17.

17. Schulze, S., et al., *A Role for Calcium-Dependent Protein Kinases in Differential CO2- and ABA-Controlled Stomatal Closing and low CO2-induced Stomatal Opening in Arabidopsis.* New Phytol, 2021. **229**(5): p. 2765-2779.

18. Sierla, M., et al., *The Receptor-like Pseudokinase GHR1 Is Required for Stomatal Closure.* Plant Cell, 2018. **30**(11): p. 2813-2837.

19. Ando, E., et al., *Elevated CO2 induces rapid dephosphorylation of plasma membrane H+-ATPase in guard cells.* New Phytologist, 2022. **236**(6): p. 2061-2074.

20. Wang, C., et al., *Reconstitution of CO2 Regulation of SLAC1 Anion Channel and Function of CO2-Permeable PIP2;1 Aquaporin as CARBONIC ANHYDRASE4 Interactor.* Plant Cell, 2016. **28**(2): p. 568-82.

21. Hashimoto, M., et al., *Arabidopsis HT1 kinase controls stomatal movements in response to CO2.* Nat Cell Biol, 2006. **8**(4): p. 391-7.

22. Hashimoto-Sugimoto, M., et al., *Dominant and recessive mutations in the Raf-like kinase HT1 gene completely disrupt stomatal responses to CO2 in Arabidopsis.* J Exp Bot, 2016. **67**(11): p. 3251-61.

23. Brearley, J., M.A. Venis, and M.R. Blatt, *The effect of elevated CO2 concentrations on K+ and anion channels of Vicia faba L. guard cells.* Planta, 1997. **203**(2): p. 145-154.

24. Tõldsepp, K., et al., *Mitogen-activated protein kinases MPK4 and MPK12 are key components mediating CO2-induced stomatal movements.* Plant J, 2018. **96**(5): p. 1018-1035.

25. Shi, K., et al., *Guard cell hydrogen peroxide and nitric oxide mediate elevated CO2 -induced stomatal movement in tomato.* New Phytol, 2015. **208**(2): p. 342-53.

26. Jalakas, P., et al., *Combined action of guard cell plasma membrane rapid- and slow-type anion channels in stomatal regulation.* Plant Physiol, 2021. **187**(4): p. 2126-2133.

27. Chater, C., et al., *Elevated CO2-Induced Responses in Stomata Require ABA and ABA Signaling.* Curr Biol, 2015. **25**(20): p. 2709-16.

28. Tian, W., et al., *A molecular pathway for CO2 response in Arabidopsis guard cells.* Nature Communications, 2015. **6**(1): p. 6057.

29. Negi, J., et al., *CO2 regulator SLAC1 and its homologues are essential for anion homeostasis in plant cells.* Nature, 2008. **452**(7186): p. 483-6.

30. Sethi, J.K., R.M. empson, and A. galione, *Nicotinamide inhibits cyclic ADP-ribose-mediated calcium signalling in sea urchin eggs.* Biochemical Journal, 1996. **319**(2): p. 613-617.

31. Walseth, T.F. and H.C. Lee, *Synthesis and characterization of antagonists of cyclic-ADP-ribose-induced Ca2+ release.* Biochim Biophys Acta, 1993. **1178**(3): p. 235-42.

32. Rakovic, S., et al., *An antagonist of cADP-ribose inhibits arrhythmogenic oscillations of intracellular Ca2+ in heart cells.* J Biol Chem, 1999. **274**(25): p. 17820-7.

33. Nishimura, N., et al., *PYR/PYL/RCAR family members are major in-vivo ABI1 protein phosphatase 2C-interacting proteins in Arabidopsis.* Plant J, 2010. **61**(2): p. 290-9.

34. Umezawa, T., et al., *Type 2C protein phosphatases directly regulate abscisic acid-activated protein kinases in Arabidopsis.* Proc Natl Acad Sci U S A, 2009. **106**(41): p. 17588-93.

35. Vlad, F., et al., *Protein phosphatases 2C regulate the activation of the Snf1-related kinase OST1 by abscisic acid in Arabidopsis.* Plant Cell, 2009. **21**(10): p. 3170-84.

36. Scherzer, S., et al., *Multiple calcium-dependent kinases modulate ABA-activated guard cell anion channels.* Mol Plant, 2012. **5**(6): p. 1409-12.

37. Geiger, D., et al., *Guard cell anion channel SLAC1 is regulated by CDPK protein kinases with distinct Ca2+ affinities.* Proc Natl Acad Sci U S A, 2010. **107**(17): p. 8023-8.

38. Hua, D., et al., *A plasma membrane receptor kinase, GHR1, mediates abscisic acid- and hydrogen peroxide-regulated stomatal movement in Arabidopsis.* Plant Cell, 2012. **24**(6): p. 2546-61.

39. Grondin, A., et al., *Aquaporins Contribute to ABA-Triggered Stomatal Closure through OST1-Mediated Phosphorylation.* Plant Cell, 2015. **27**(7): p. 1945-54.

40. Li, S., S.M. Assmann, and R. Albert, *Predicting essential components of signal transduction networks: a dynamic model of guard cell abscisic acid signaling.* PLoS Biol, 2006. **4**(10): p. e312.

41. Sasaki, T., et al., *Closing plant stomata requires a homolog of an aluminum-activated malate transporter.* Plant Cell Physiol, 2010. **51**(3): p. 354-65.

42. Kinoshita, T., M. Nishimura, and K. Shimazaki, *Cytosolic Concentration of Ca2+ Regulates the Plasma Membrane H+-ATPase in Guard Cells of Fava Bean.* Plant Cell, 1995. **7**(8): p. 1333-1342.

43. Ward, J.M. and J.I. Schroeder, *Calcium-Activated K+ Channels and Calcium-Induced Calcium Release by Slow Vacuolar Ion Channels in Guard Cell Vacuoles Implicated in the Control of Stomatal Closure.* Plant Cell, 1994. **6**(5): p. 669-683.

44. Guse, A.H., *Cyclic ADP-ribose: a novel Ca2+-mobilising second messenger.* Cell Signal, 1999. **11**(5): p. 309-16.

45. Maheshwari, P., et al., *Model-driven discovery of calcium-related protein-phosphatase inhibition in plant guard cell signaling.* PLoS Comput Biol, 2019. **15**(10): p. e1007429.

46. Swatek, K.N., et al., *Multisite phosphorylation of 14-3-3 proteins by calcium-dependent protein kinases.* Biochem J, 2014. **459**(1): p. 15-25.

47. Brandt, B., et al., *Reconstitution of abscisic acid activation of SLAC1 anion channel by CPK6 and OST1 kinases and branched ABI1 PP2C phosphatase action.* Proc Natl Acad Sci U S A, 2012. **109**(26): p. 10593-8.

48. Mulaudzi, T., et al., *Identification of a novel Arabidopsis thaliana nitric oxide-binding molecule with guanylate cyclase activity in vitro.* FEBS Lett, 2011. **585**(17): p. 2693-7.

49. Nagy, S.K., et al., *Activation of AtMPK9 through autophosphorylation that makes it independent of the canonical MAPK cascades.* Biochem J, 2015. **467**(1): p. 167-75.

50. Desikan, R., et al., *A new role for an old enzyme: nitrate reductase-mediated nitric oxide generation is required for abscisic acid-induced stomatal closure in Arabidopsis thaliana.* Proc Natl Acad Sci U S A, 2002. **99**(25): p. 16314-8.

51. Joudoi, T., et al., *Nitrated cyclic GMP modulates guard cell signaling in Arabidopsis.* Plant Cell, 2013. **25**(2): p. 558-71.

52. Sokolovski, S. and M.R. Blatt, *Nitric oxide block of outward-rectifying K+ channels indicates direct control by protein nitrosylation in guard cells.* Plant Physiol, 2004. **136**(4): p. 4275-84.

53. Sirichandra, C., et al., *Phosphorylation of the Arabidopsis AtrbohF NADPH oxidase by OST1 protein kinase.* FEBS Lett, 2009. **583**(18): p. 2982-6.

54. Acharya, B.R., et al., *Open Stomata 1 (OST1) is limiting in abscisic acid responses of Arabidopsis guard cells.* New Phytol, 2013. **200**(4): p. 1049-63.

55. Imes, D., et al., *Open stomata 1 (OST1) kinase controls R-type anion channel QUAC1 in Arabidopsis guard cells.* Plant J, 2013. **74**(3): p. 372-82.

56. Meyer, S., et al., *AtALMT12 represents an R-type anion channel required for stomatal movement in Arabidopsis guard cells.* Plant J, 2010. **63**(6): p. 1054-62.

57. Meinhard, M. and E. Grill, *Hydrogen peroxide is a regulator of ABI1, a protein phosphatase 2C from Arabidopsis.* FEBS Lett, 2001. **508**(3): p. 443-6.

58. Meinhard, M., P.L. Rodriguez, and E. Grill, *The sensitivity of ABI2 to hydrogen peroxide links the abscisic acid-response regulator to redox signalling.* Planta, 2002. **214**(5): p. 775-82.

59. Sridharamurthy, M., et al., *H2O2 inhibits ABA-signaling protein phosphatase HAB1.* PLoS One, 2014. **9**(12): p. e113643.

60. Bright, J., et al., *ABA-induced NO generation and stomatal closure in Arabidopsis are dependent on H2O2 synthesis.* Plant J, 2006. **45**(1): p. 113-22.

61. Zhang, X., et al., *Inhibition of blue light-dependent H+ pumping by abscisic acid through hydrogen peroxide-induced dephosphorylation of the plasma membrane H+-ATPase in guard cell protoplasts.* Plant Physiol, 2004. **136**(4): p. 4150-8.

62. Kohler, B., A. Hills, and M.R. Blatt, *Control of guard cell ion channels by hydrogen peroxide and abscisic acid indicates their action through alternate signaling pathways.* Plant Physiol, 2003. **131**(2): p. 385-8.
