# Supplementary material for: A network-based modeling framework reveals the core signal transduction network underlying high carbon dioxide-induced stomatal closure in guard cells
Source: PLoS Biol. 2024 May 1;22(5):e3002592. doi: 10.1371/journal.pbio.3002592 (PMC11090369; doi:10.1371/journal.pbio.3002592)
Supplement: S1 Text — (DOCX) [file pbio.3002592.s005.docx]

**S1 Text. Regulatory functions of the dynamic model and their justifications**

In this document we indicate the assumed state of the source nodes (inputs), explain the Boolean regulatory functions of the model, and describe the assumed initial state of the nodes. In each simulation, we initialize all input nodes as their assumed states corresponding to open stomata (described in section B).

1. **Summary table of regulatory functions**

| **Node** | **Regulatory function** |
| --- | --- |
| CO_2_ entry of cell | CO_2_ porins and high CO_2_ |
| HCO_3_^-^ | CAs and CO_2_ entry of cell |
| RHC1 | CAs and HCO_3_^-^ |
| HT1 | not MPKs-HT1 complex and not MPK activity |
| OST1 minimum function | not ABI1 and not ABI2 and not HAB1 or not HT1 |
| OST1 activated | OST1 minimum function and not ABI1 and not ABI2 and not HAB1 and not HT1 and ABA |
| GHR1 | ROS and not HT1 and not ABI2 |
| MPKs-HT1 complex | MPKs and HCO_3_^-^ |
| MPK | MPKs and (Ca^2+^_cyt_ or MPK activity) |
| CBC1/2 | HT1 |
| ABI1 | not ROS and not CaIM |
| ABI2 | not ROS and not CaIM |
| HAB1 | not ROS and not CaIM |
| RbohDF | OST1 minimum function |
| CIS | cADPR |
| cADPR | NAD^+^ and ADPRc |
| ADPRc | 8-nitro-cGMP |
| 8-nitro-GMP | cGMP and NO |
| cGMP | NOGC and GTP |
| NOGC | NO |
| NIA1/2 | ROS |
| NO | NIA1/2 and Nitrite and NADPH |
| ROS | RbohD/F and NADPH |
| CaIM | GHR1 |
| Ca^2+^_cyt_ | (CIS or CaIM) and not Ca^2+^ATPase |
| Ca^2+^ ATPase | Ca^2+^_cyt_ |
| CPKs | Ca^2+^_cyt_ or GHR1 or CPKs |
| SLAC1 generic | not ABI1 and not ABI2 and GHR1 and MPKs kinase and OST1 minimum function and (CPKs or OST1 activated) |
| SLAC1 CO_2_ | not ABI1 and not ABI2 and GHR1 and OST1 minimum function and HCO_3_^-^ and not CBC1/2 |
| QUAC1 | Ca^2+^_cyt_ and OST1 minimum function |
| H^+^ATPase | not Ca^2+^_cyt_ and not ROS |
| AnionEM | SLAC1 general or SLAC1 CO_2_ or QUAC1 |
| KEV | Ca^2+^_cyt_ |
| Kout | Depolarization |
| Depolarization | (AnionEM or Ca^2+^_cyt_ or KEV) and (not H^+^ATPase or not K^+^ efflux) |
| K^+^ efflux | Kout and KEV or Kout and K^+^ efflux |
| Aquaporins | OST1 minimum function |
| H_2_O efflux | AnionEM and K^+^ efflux and Aquaporins |
| Closure | H_2_O efflux |

1. **Source nodes, not regulated by any other node of the network**

The input signal High CO_2_ may be OFF (representing ambient CO_2_ of around 450 ppm) or ON (representing elevated CO_2_ of around 800 ppm). ABA is an input node necessary for capturing responses not observed under high CO_2_, e.g. OST1 activation. ABA is assumed to be OFF. The rest of the input nodes are assumed to take the default state that helps activate closure; namely ON.

**High CO_2_**

**ABA (assumed OFF)**

**CO_2_ porins**

**CAs**

**GTP**

**MPKs**

**NAD^+^**

**NADPH**

**Nitrite**

1. **Boolean regulatory functions of regulated nodes**

**CO_2_ entry of cell * = CO_2_ porins and high CO_2_**

We assume CO_2_ enters the cell by CO_2_ porins, which are conceptually similar to aquaporins, with PIP2;1 being one of them [1].

**HCO_3_^-^ * = CAs and CO_2_ entry of cell**

The CO_2_-binding carbonic anhydrase proteins CA1 and CA4 catalyze the reversible reaction of CO_2_ + H_2_O → HCO_3_ ^–^ + H^+^. This reaction likely functions early in CO_2_ signaling [2, 3]. This process requires CO_2_ entry into the cell. Arabidopsis double mutant plants in the β-carbonic anhydrases, βCA1 and βCA4, display impaired CO_2_-regulation of stomatal movements [2, 3]. Carbonic anhydrase protein *ca7* mutant alleles also display a compromised response to elevated CO_2_ [4].

**RHC1 * = CAs and HCO_3_^-^**

The *rhc1* mutant is found irresponsive to CO_2_ [5, 6]. RHC1 can interact with CA4 and HT1 in the plasma membrane, perceives HCO_3_^-^ and removes the inhibitory effect of HT1 [5, 7]. Since CAs is necessary in high CO_2_ induced closure, we use the “AND” operator to connect the two regulators of RHC1.

**HT1 * = not MPKs-HT1 complex and not MPK activity**

Multiple *ht1* mutants show irresponsiveness to high CO_2_ [8-10]. An important consideration for the regulatory function of the protein kinase HT1 is the interpretation of its active (ON) and inactive (OFF) state. There’s no clear evidence showing which biochemical states HT1’s binary states correspond to. For example, the *ht1-8D* mutant has normal kinase activity yet loses all sensitivity to CO_2_ [10], meaning that it is not (or at least not completely) the kinase activity that mediates CO_2_ response. To address this challenge, we define HT1’s binary state to reflect its effect on stomatal closure, ON being the “closure-inhibiting state” and OFF being the “closure promoting/non-inhibiting state”. Experimental evidence show that the *ht1-1* and *ht1-2* mutants have closed stomata with insensitivity to CO_2_ [8]; on the other hand, the *ht1-3D* and *ht1-8D* dominant mutants have open stomata with insensitivity to CO_2_ [9, 10]. According to these observations, we represent the *ht1-3/ht-8* mutants as HT1=ON and assign HT1=OFF to the *ht1-2* mutant.

MPK4 and 12 interact with HT1 and function as an inhibitor of HT1 [10, 11]. Furthermore, recent studies found that elevated HCO_3_^-^ triggers an interaction and complex formation between MPKs and HT1, inhibiting HT1 kinase activity, and this interaction does not require MPK kinase activity [12, 13]. So, we define a node named “MPKs-HT1complex” (activated by HCO_3_^-^) as a regulator of HT1. We also assume a CO_2_ -independent MPK activity (possibly a kinase activity, but not necessarily so) that can inhibit HT1 as this is required for the model to recapitulate the closure observed in interventions under ambient CO_2_ (e.g. under exogenous Ca^2+^). Note that the node “MPK activity” does not function as a CO_2_ receptor in the network, and model simulation recapitulates the experimental observation that loss of MPK kinase activity doesn’t impair high CO_2_ induced closure.

RHC1 perceives HCO_3_^-^ and was found to remove the inhibitory effect of HT1 on the OST1-induced activation of SLAC1 [5]. In contrast, Tõldsepp et al. found that RHC1’s function in CO_2_ signaling is minor [6]. We tested both versions of the HT1 regulatory function, with and without RHC1, and found the function without RHC1 (as presented above) has a higher accuracy in capturing experimental observations. Therefore, we adopt the HT1 function without RHC1, consistent with Tõldsepp et al. The interpretation is that RHC1’s regulation of HT1 is weaker than that of MPKs’, thus insufficient to include in a Boolean model; it should not be interpreted as RHC1 having no regulatory effect on HT1.

**OST1 minimum function *= not ABI1 and not ABI2 and not HAB1 or not HT1**

**OST1 activated *= OST1 minimum function and not ABI1 and not ABI2 and not HAB1 and not HT1 and ABA**

In contrast to ABA signaling, the kinase activity of OST1 is found not to elevate under high CO_2_ [14]. This inspired us to split OST1 into two separate nodes, “OST1 minimum function” and “OST1 activated”, to better recapitulate these observations. “OST1 activated” represents the strong kinase activity of OST1 observed in ABA signaling. We define OST1 minimum function as the minimum activity of OST1 necessary for promotion of closure. The constant OFF state of OST1 minimum function represents the *ost1* KO mutant, which loses closure response to ABA and CO_2_ [3, 15]. The ON state of OST1 minimum function may represent a basal OST1 kinase activity (as observed under low CO_2_ or high CO_2_ [14]) or it may arise from OST1’s complex-forming capacity, or may be a combination of these two mechanisms, as long as this function is necessary to promote closure.

The PP2C protein phosphatases (ABI1, ABI2 and HAB1) physically interact with OST1 and inhibit its kinase activity [16-18]. ABI1 and ABI2 inhibit SLAC1 by inhibiting the OST1-induced SLAC1 activation [19]. Because *abi1/abi2* mutants show an impaired closure response consistent with the loss of closure response in the *ost1* KO mutant, we assume PP2Cs inhibit both OST1 nodes. By a similar logic, because HT1 inhibits OST1 and inhibits the phosphorylation of SLAC1 by OST1 [5, 10], and the *ht1* mutants lose high CO_2_ response, we assume that HT1 inhibits both OST1 nodes as well. In addition, to ensure that OST1 activated is OFF under high CO_2_ and ON in response to other signals (e.g. ABA), it must have at least one other signal input. Thus, we implement that ABA activates OST1 activated (e.g., via RCARs).

To maximize model consistency with observations, we connect HT1 with an “AND” operator in the regulatory function of OST1 activated (thus, OST1 activated is OFF under high CO_2_), and we connect HT1 with an “OR” operator in the regulatory function of OST1 minimum function (otherwise high CO_2_ would not be able to induce closure). It is common modeling practice to have a looser condition (logic “OR” gate) in the low/minimum activity function, and a more stringent one (logic “AND” gate) in the high activity function.

**GHR1 *= ROS and not HT1 and not ABI2**

The *ghr1* loss-of-function mutant does not show ROS-induced stomatal closure [20], thus we assume that ROS activates GHR1. ABI2 inhibits GHR1 and GHR1-mediated ROS signal propagation. GHR1 physically interacted with, phosphorylated, and activated SLAC1 when co-expressed in *Xenopus laevis* oocytes, and this activation was inhibited by ABA-INSENSITIVE2 (ABI2) but not ABI1 [20]. In addition, HT1 is found to inhibit SLAC1 by inhibiting GHR1 in *Xenopus laevis* oocytes [5, 10]. According to the mutant experiments, perturbing any of the inputs will cause reduced CO_2_ induced closure, so they are connected with “AND” operators.

**MPKs-HT1 complex *= MPKs and HCO_3_^-^**

Lack of MPK4 and MPK12 in guard cells abolishes stomatal CO_2_ responses [7, 10, 21]. MPK4/12 interact with HT1 and inhibit its kinase activity, when triggered by bicarbonate [13]. This inhibition does not require MPKs’ kinase activity. We implement the MPKs-HT1 inhibition mechanism as separate node MPKs-HT1 complex, to distinguish from the MPKs’ kinase activity. The formation of this complex requires functional MPK proteins and HCO_3_^-^.

**MPK activity *= MPKs and (Ca^2+^_cyt_ or MPK activity)**

The node “MPK activity” aims to fill a gap of understanding regarding closure driven by providing ROS, cADPR, 8-nitro-cGMP, or external Ca^2+^ under ambient CO_2._ The mechanism of HT1 inactivation via HCO_3_^-^ - driven MPK-HT1 complex formation [12, 13] does not apply in these settings. Yet, a mechanism must exist to inactivate HT1 in these conditions, to disrupt its inhibitory effect on anion flow and closure. This node represents MPKs activity (including kinase activity) related to stomatal closure but not specific to high CO_2_-induced closure. Lack of MPK4 and MPK12 in guard cells abolishes stomatal CO_2_ responses [7, 10, 21]. Furthermore, Ca^2+^ fails to activate anion currents in *mpk9/mpk12* double mutants [21], indicating that cytosolic Ca^2+^ affects the expression or function of the MPKs. In the absence of further mechanistic information, we assume that MPKs mediate the Ca^2+^ induced SLAC1 activation via their kinase activity, which is distinct from MPKs’ role as a CO_2_ receptor. There is evidence of autophosphorylation for MPKs [22], so we include the node itself in its regulatory function, representing the assumption that continued kinase activity can be maintained if there is sufficient protein abundance.

We note that an alternative mediator of an inhibition of HT1 by Ca^2+^ would yield equivalent results in the model.

**CBC1/2 *= HT1**

CBC1/CBC2 interact with and are phosphorylated by HT1; the *cbc1-cbc2* mutant phenocopies the *ht1-2* mutant in being irresponsive to high CO_2_ and having a low stomatal conductance [13, 23]. This evidence supports HT1 being the sole regulator of CBC1/2.

**ABI1 *= not ROS and not CaIM**

ROS inhibit ABI1 activity [24]. Calcium is found to inhibit PP2Cs [25]. We assume CaIM (Ca^2+^ influx through the membrane) inhibits PP2Cs, because Ca^2+^_cyt_ (cytosolic calcium) oscillates in this model and thus cannot provide a stable inhibition. This assumption is equivalent with assuming a sustained effect of Ca^2+^_cyt_=ON. We assume “AND” operators between ABI1 inhibitors so that any of the inputs can inhibit ABI1. A weaker inhibition of ABI1 would obstruct closure in response to external Ca^2+^ or ROS.

**ABI2 *= not ROS and not CaIM**

ROS inhibit ABI2 activity [26]. Calcium is found to inhibit PP2Cs [25], so we assume CaIM inhibits PP2Cs, because Ca^2+^_cyt_ oscillates in this model thus cannot provide a stable inhibition. We assume “AND” operators between ABI2 inhibitors so that any of the inputs can inhibit ABI2, otherwise ABI2 would inhibit closure.

**HAB1 *= not ROS and not CaIM**

H_2_O_2_ (ROS) inhibits HAB1’s catalytic activity [27]. Calcium was found to inhibit PP2Cs [25], so we assume CaIM inhibits PP2Cs, because Ca^2+^_cyt_ oscillates in this model thus cannot provide a stable inhibition. We assume “AND” operators between inhibitors so that any of the inputs can inhibit HAB1, otherwise HAB1 would inhibit closure.

**RbohDF * = OST1 minimum function**

RBOH enzymes catalyze ROS production in guard cells in response to ABA-activated signaling. We extract the generic evidence relating to RBOH and ROS production, and do not include ABA specific evidence. OST1 physically interacts with both RbohD and RbohF, phosphorylates them and increases their enzyme activity [28]. We assume that the OST1 minimum function is sufficient for the activation of RBOH.

**CIS *= cADPR**

cADPR is an important signaling molecule leading to calcium release from internal stores [29].

**cADPR *= NAD^+^ and ADPRc**

ADPRc (enzyme) uses NAD^+^ (substrate) for production of cADPR.

**ADPRc *= 8-nitro-cGMP**

ADPRc antagonist nicotinamide [30] and cADPR antagonist 8-bromo-cADPR [31, 32] inhibit 8-nitro-cGMP mediated stomatal closure, suggesting that cADPR and ADPRc are downstream of 8-nitro-cGMP. We therefore assume 8-nitro-cGMP regulates ADPRc.

**8-nitro-GMP *= cGMP and NO**

NO activates 8-nitro-cGMP synthesis [33]. cGMP is the substrate in this reaction and is required to be present. ROS is found to be necessary for ABA-induced 8-nitro-cGMP synthesis [33]. Since ROS is required to activate NO via NIA1/2, we assume only NO is directly required in 8-nitro-cGMP function (ROS is still required implicitly, as ROS is required for NO). Both inputs are necessary, thus they are connected with the “AND” operator.

**cGMP *= NOGC and GTP**

NOGC1 (enzyme) uses GTP (substrate) for the production of cGMP [34].

**NOGC *= NO**

NO promotes the enzyme activity of the guanylate cyclase NOGC1 [34].

**NIA1/2 *= ROS**

NIA1 and NIA2 are nitrate reductases. In the *nia1* *nia2* double mutant (loss-of-function) ROS fails to induce NO production [35], so we assume ROS regulates NIA1/2.

**NO *= NIA1/2 and Nitrite and NADPH**

Nitrite, NIA1/2, and NADPH comprise a substrate, enzyme, and coenzyme relationship respectively [36]; all three are required for NO production. Nitrite and NADPH are input nodes assumed ON.

**ROS *= RbohD/F and NADPH**

RBOH and NADPH have an enzyme and coenzyme relationship that is explained by an AND rule. NADPH is an input node assumed ON.

**CaIM* = GHR1**

*ghr1* loss-of-function mutants show impaired Ca^2+^ influx through non-stretch-activated Ca^2+^ channels [20], so we assume GHR1 regulates CaIM.

**Ca^2+^_cyt_ * = (CIS or CaIM) and not Ca^2+^ATPase**

Both CIS and CaIM (Ca­­^2+^ influx from stores and across the plasma membrane, respectively) can cause increase of cytosolic Ca^2+^. The Ca­­^2+^ATPase pumps Ca­­^2+^ from the cytosol to the apoplast.

**Ca^2+^ ATPase* = Ca^2+^_cyt_**

The mechanisms that mediate Ca^2+^ efflux from the cytosolic compartment are incorporated by including Ca^2+^ATPases and Ca^2+^/H^+^ antiporters as the node “Ca^2+^ATPase” in our model. Calcium oscillations occur in response to ABA. This rule is the simplest way to incorporate Ca^2+^_cyt_ concentration oscillations in our model [37].

**CPKs* = Ca^2+^_cyt_ or GHR1 or CPKs**

The node CPKs represents multiple Ca^2+^-dependent protein kinases. Schulze et al. in [38] found that cpk3/5/6/11/23 quintuple mutant plants were defective in high CO_2_-induced stomatal closure. Cytosolic calcium activates CPK3 and CPK21 by binding to their EF hand calcium binding motifs, so we implement Ca^2+^_cyt_ as CPKs’ regulator. Multiple CPKs are known to auto-phosphorylate [39], so we implement this self-sustaining effect as including “or CPKs” in the function. GHR1 is found to interact with CPK3 [40]; as there are no further information about regulators of other CPKs, we assume GHR1 regulates the CPKs node in our model.

**SLAC1 generic* = not ABI1 and not ABI2 and GHR1 and MPKs kinase and OST1 minimum function and (CPKs or OST1 activated)**

**SLAC1 CO_2_* = not ABI1 and not ABI2 and GHR1 and OST1 minimum function and HCO_3_^-^ and not CBC1/2**

We adopt a two-node representation for SLAC1, to better capture its CO_2_-specific and general (not CO_2_ specific) responses. The HCO_3_^-^-induced activation of S-type anion channels is reduced in the dominant negative PP2C mutants *abi1-1* and *abi2-1* [41]. ABI1 and ABI2 have been shown to inhibit SLAC1 by inhibiting OST1-mediated SLAC1 activation [19], and CPK23-, CPK21- and CPK6-mediated activation of SLAC1 in *Xenopus* oocytes [42]. We implement these as ABI1 and ABI2 inhibiting both SLAC1 nodes. Guard cells of the *mpk9 mpk12* double mutant do not show Ca^2+^ activation of slow anion channel activity [21], so we assume cytosolic Ca^2+^ regulates “SLAC1 general” via the MPKs’ kinase activity, and used an “AND” operator to connect MPKs kinase to the other regulators. GHR1 activates SLAC1 activity. The *ghr1* knockout mutant shows impaired ABA and ROS activation of slow anion channels [20]. Guard cells of the *ost1* mutant show impaired closure response under both ABA and CO_2_, so OST1 minimum function is a required activator of SLAC1 [15, 19]. Because the mutants of GHR1 and OST1 show reduced SLAC1 activity, we interpreted these inputs as necessary for SLAC1 and connect them with the “AND” operator.

Elevation of OST1 kinase activity is not observed under high CO_2_ induced closure, meaning that OST1 activation is not necessary for SLAC1 activation. All indicated CPKs activate SLAC1 activity by phosphorylation [42-44]. However, CPK and OST1 phosphorylate the N terminal of SLAC1, whose removal does not affect CO_2_ induced SLAC1 activation. Based on these we implement CPKs and OST1 activated only in node “SLAC1 generic”.

The R256 residue of SLAC1 is required for CO_2_ regulation of stomatal movements, but not for ABA-induced stomatal closing. Patch clamp analyses show that activation of S-type anion channels by high CO_2_/HCO_3_^-^, but not by ABA, was impaired when deleting the R256 residue [45]. We incorporate this mechanism of CO_2_-specific SLAC1 activation by including HCO_3_^-^ as a necessary regulator of “SLAC1 CO_2_”. CBC1/2 are known inhibitors of SLAC1 activity [23], and we included them as inhibitors specific to CO_2_ based on the conclusion that they are at the intersection of blue light and CO_2_ signaling [13].

**QUAC1* = Ca^2+^_cyt_ and OST1 minimum function**

Plants lacking QUAC1 (i.e. AtALMT12) display slower CO₂ -induced stomatal closure [46], [47]. OST1 physically interacts with QUAC1 and activates this R-type anion channel [48]. The QUAC1 loss-of-function mutant does not close in response to external Ca^2+^ [49], indicating that QUAC1 is an effector in Ca^2+^**-**mediated stomatal closure, so we implement Ca^2+^_cyt_ as an input for QUAC1. We connect the inputs with “AND” operator to maximize model consistency with observations. If we assumed that OST1 minimum function alone can fully activate QUAC1, causing AnionEM, the observation of impaired closure in response to high CO_2_ in the *slac1* KO mutants would not be recapitulated.

**H^+^ATPase *= not Ca^2+^_cyt_ and not ROS**

The H^+^ ATPase has been shown to be inhibited independently by reactive oxygen species [50] and cytosolic Ca^2+^concentration increase [51]. Since either input can inhibit the H^+^ ATPase, we use the “AND” operator to connect them.

**AnionEM* = SLAC1 general or SLAC1 CO_2_ or QUAC1**

Anion efflux across the membrane (AnionEM) is the result of anion flows from SLAC1, QUAC1 and SLAH3 channels. There is no specific evidence showing SLAH3’s function under CO_2_ signaling, so we do not include SLAH3 in AnionEM’s function. The *slac1* mutations impair CO_2_-, ABA- and dark-induced stomatal closure [52]. The *quac*1 loss-of-function mutants show delayed stomatal closure in response to CO_2_ [46]. The *slac1 quac1* double mutans shows more impairment in CO_2_-induced stomatal closure than either single mutant [47]. We connect SLAC1 and QUAC1 with the ‘OR’ operator as this maximizes model consistency with observations, as shown in the validation SI file.

**KEV* = Ca^2+^_cyt_**

Calcium induces K^+^ release through K^+^-permeable channels in the tonoplast [53].

**Kout *= Depolarization**

Membrane depolarization drives K^+^ efflux from the guard cell. Outwardly rectifying K^+^ channels are also inhibited by ROS [54] and nitric oxide [55], but we assume these inhibitions are weaker than the threshold implicit in Boolean models. Assuming otherwise would cause Kout to be ON under ambient CO_2_, which is not consistent with observations. So, the regulatory function of Kout contains only Depolarization.

**Depolarization * = (AnionEM or Ca^2+^_cyt_ or KEV) and (not H^+^ATPase or not K^+^ efflux)**

Efflux of cations or anions will hyperpolarize or depolarize the cell membrane, respectively. Therefore, we collect the relevant nodes as inputs here. To connect them, we assume at least one positive regulator (Anion efflux, Ca­­^2+^ influx across the plasma membrane, or release of K^+^ from the vacuole) must be ON and at least one negative regulator (K^+^ efflux across the membrane or H^+^ ATPase activity) must be off [37].

**K^+^ efflux* = Kout and KEV or Kout and K^+^ efflux**

Sustained efflux of K^+^ from the guard cell requires K^+^ efflux from the vacuole to the cytosol (KEV) followed by K^+^ efflux from the cytosol to the apoplast, mediated by outwardly rectifying K^+^ channels (Kout). So we use “AND” operator to connect Kout and KEV. In addition, we assume that K^+^ efflux can be maintained under constant Kout (this is represented by the “Kout and K^+^ efflux” part of the function). This assumed maintenance is necessary as cytosolic Ca^2+^ is known to oscillate, which would cause KEV to oscillate, preventing a long enough activation of K^+^ efflux necessary for stomatal closure.

**Aquaporins *= OST1 minimum function**

Aquaporins are water channels that are necessary for water efflux from the cell. OST1-mediated phosphorylation activates aquaporin Plasma membrane Intrinsic Protein 2;1 (PIP2;1) in guard cells in response to ABA [56]. We assume OST1 minimum function is sufficient for Aquaporins; otherwise high CO_2_ induced closure would not be possible.

**H_2_O efflux* = AnionEM and K^+^ efflux and Aquaporins**

**Closure* = H_2_O efflux**

Both anion and K^+^ efflux are required for H_2_O efflux, which drives stomatal closure. In addition, Aquaporins (e.g. PIP2;1) facilitate water efflux, and *pip2;1* mutant displays impaired closure under ABA and CO2 [1, 56]. Finally, Closure is caused by water efflux.

1. **Initial states of nodes in simulation**

We define the initial states of (non-source) nodes as their expected state in open stomata. As a result, all closure inhibitors, namely CBC1/2, HT1, ABI1, ABI2, HAB1, and H^+^ ATPase, are initialized in their ON state. The rest of the nodes are initialized OFF. These initial states are the opposite of the attractor with Closure ON (the first row of Figure 3C).

Because under high CO_2_ there’s a unique attractor, trajectories from any initial condition converge to this closure ON attractor. Conversely, multiple attractors exist under ambient CO_2_ (see Figure 4D), thus trajectories that start from different initial states of the system could converge to different attractors. For example, initializing HT1 at OFF instead of ON under ambient CO_2_ has a chance to yield a closure ON attractor. As a confirmation, we simulate the network model with one inhibitor initialized OFF and the other five initialized ON, and considered each inhibitor in turn to be the one initialized OFF. We confirmed that HT1=OFF is the only initial condition that leads to a nonzero percentage of closure, as shown in the table below. The reason HT1=OFF can lead to closure in certain simulations is that HT1=OFF is a driver of the Main Stable Motif. If HT1 is OFF for sufficiently long, it will lock-in the MSM, leading to a closure ON attractor. Stable motif-based attractor identification yields all the attractors of the system; thus, it implicitly considers all the initial conditions.

|  | CBC1/2 | HT1 | ABI1 | ABI2 | HAB1 | H_+_ ATPase |
| --- | --- | --- | --- | --- | --- | --- |
| Percentage Closure, if the inhibitor is initialized OFF | 0% | 19.4% | 0% | 0% | 0% | 0% |

1. **Pseudo-code of all regulatory functions**

Below we include a comprehensive list of all Boolean functions explained above, in a slightly modified format that facilitates computer simulation, e.g., HCO3 for HCO_3_^-^.

CO2_entry_of_cell * = CO2_porins and high_CO2

HCO3 * = CAs and CO2_entry_of_cell

RHC1 * = CAs and HCO3

HT1 * = not MPK_kinase_activity and not MPKsHT1complex

OST1_minimum_function *= not ABI1 and not ABI2 and not HAB1 or not HT1

OST1_activated *= OST1_minimum_function and not ABI1 and not ABI2 and not HAB1 and not HT1 and ABA

GHR1 *= ROS and not HT1 and not ABI2

MPK_kinase_activity *= MPKs and (Ca2+_cyt or MPK_kinase_activity)

MPKsHT1complex * = MPKs and HCO3

CBC12 *= HT1

ABI1 *= not ROS and not CaIM

ABI2 *= not ROS and not CaIM

HAB1 *= not ROS and not CaIM

RbohDF * = OST1_minimum_function

CIS *= cADPR

cADPR *= NAD+ and ADPRc

ADPRc *= 8nitro_cGMP

8nitro_cGMP *= cGMP and NO

cGMP *= NOGC and GTP

NOGC *= NO

NIA1/2 *= ROS

NO *= NIA1/2 and Nitrite and NADPH

ROS *= RbohDF and NADPH

CaIM* = GHR1

Ca2+_cyt* = (CIS or CaIM) and not Ca_ATPase

Ca_ATPase* = Ca2+_cyt

CPKs* = Ca2+_cyt or GHR1 or CPKs

SLAC1_generic* = not ABI1 and not ABI2 and GHR1 and MPK_kinase_activity and OST1_minimum_function and (CPKs or OST1_activated)

SLAC1_CO2* = not ABI1 and not ABI2 and GHR1 and OST1_minimum_function and HCO3 and not CBC12

QUAC1* = Ca2+_cyt and OST1_minimum_function

H+_ATPase *= not Ca2+_cyt and not ROS

AnionEM* = SLAC1_generic or SLAC1_CO2 or QUAC1

KEV* = Ca2+_cyt

Kout *= Depolarization

Depolarization * = (AnionEM or Ca2+_cyt or KEV) and (not H+_ATPase or not K+_efflux)

K+_efflux* = Kout and KEV or Kout and K+_efflux

Aquaporins *= OST1_minimum_function

H2O_Efflux* = AnionEM and K+_efflux and Aquaporins

Closure* = H2O_Efflux

**References**

1. Wang, C., et al., *Reconstitution of CO2 Regulation of SLAC1 Anion Channel and Function of CO2-Permeable PIP2;1 Aquaporin as CARBONIC ANHYDRASE4 Interactor.* Plant Cell, 2016. **28**(2): p. 568-82.

2. Hu, H., et al., *Carbonic anhydrases are upstream regulators of CO2-controlled stomatal movements in guard cells.* Nat Cell Biol, 2010. **12**(1): p. 87-93; sup pp 1-18.

3. Xue, S., et al., *Central functions of bicarbonate in S-type anion channel activation and OST1 protein kinase in CO2 signal transduction in guard cell.* EMBO J, 2011. **30**(8): p. 1645-58.

4. Sun, P., et al., *Countering elevated CO2 induced Fe and Zn reduction in Arabidopsis seeds.* New Phytologist, 2022. **235**(5): p. 1796-1806.

5. Tian, W., et al., *A molecular pathway for CO2 response in Arabidopsis guard cells.* Nature Communications, 2015. **6**(1): p. 6057.

6. Tõldsepp, K., et al., *Mitogen-activated protein kinases MPK4 and MPK12 are key components mediating CO2-induced stomatal movements.* Plant J, 2018. **96**(5): p. 1018-1035.

7. Tõldsepp, K., et al., *Mitogen-activated protein kinases MPK4 and MPK12 are key components mediating CO.* Plant J, 2018. **96**(5): p. 1018-1035.

8. Hashimoto, M., et al., *Arabidopsis HT1 kinase controls stomatal movements in response to CO2.* Nat Cell Biol, 2006. **8**(4): p. 391-7.

9. Hashimoto-Sugimoto, M., et al., *Dominant and recessive mutations in the Raf-like kinase HT1 gene completely disrupt stomatal responses to CO2 in Arabidopsis.* J Exp Bot, 2016. **67**(11): p. 3251-61.

10. Hõrak, H., et al., *A Dominant Mutation in the HT1 Kinase Uncovers Roles of MAP Kinases and GHR1 in CO2-Induced Stomatal Closure.* Plant Cell, 2016. **28**(10): p. 2493-2509.

11. Jakobson, L., et al., *Natural Variation in Arabidopsis Cvi-0 Accession Reveals an Important Role of MPK12 in Guard Cell CO2 Signaling.* PLoS Biol, 2016. **14**(12): p. e2000322.

12. Yeh, C.-Y., et al., *MPK12 in stomatal CO2 signaling: function beyond its kinase activity.* New Phytologist, 2023. **n/a**(n/a).

13. Takahashi, Y., et al., *Stomatal CO2/bicarbonate sensor consists of two interacting protein kinases, Raf-like HT1 and non-kinase-activity requiring MPK12/MPK4.* Science Advances, 2022. **8**(49): p. eabq6161.

14. Hsu, P.K., et al., *Abscisic acid-independent stomatal CO2 signal transduction pathway and convergence of CO2 and ABA signaling downstream of OST1 kinase.* Proc Natl Acad Sci U S A, 2018. **115**(42): p. E9971-E9980.

15. Acharya, B.R., et al., *Open Stomata 1 (OST1) is limiting in abscisic acid responses of Arabidopsis guard cells.* New Phytol, 2013. **200**(4): p. 1049-63.

16. Nishimura, N., et al., *PYR/PYL/RCAR family members are major in-vivo ABI1 protein phosphatase 2C-interacting proteins in Arabidopsis.* Plant J, 2010. **61**(2): p. 290-9.

17. Umezawa, T., et al., *Type 2C protein phosphatases directly regulate abscisic acid-activated protein kinases in Arabidopsis.* Proc Natl Acad Sci U S A, 2009. **106**(41): p. 17588-93.

18. Vlad, F., et al., *Protein phosphatases 2C regulate the activation of the Snf1-related kinase OST1 by abscisic acid in Arabidopsis.* Plant Cell, 2009. **21**(10): p. 3170-84.

19. Geiger, D., et al., *Activity of guard cell anion channel SLAC1 is controlled by drought-stress signaling kinase-phosphatase pair.* Proc Natl Acad Sci U S A, 2009. **106**(50): p. 21425-30.

20. Hua, D., et al., *A plasma membrane receptor kinase, GHR1, mediates abscisic acid- and hydrogen peroxide-regulated stomatal movement in Arabidopsis.* Plant Cell, 2012. **24**(6): p. 2546-61.

21. Jammes, F., et al., *MAP kinases MPK9 and MPK12 are preferentially expressed in guard cells and positively regulate ROS-mediated ABA signaling.* Proc Natl Acad Sci U S A, 2009. **106**(48): p. 20520-5.

22. Nagy, S.K., et al., *Activation of AtMPK9 through autophosphorylation that makes it independent of the canonical MAPK cascades.* Biochem J, 2015. **467**(1): p. 167-75.

23. Hayashi, M., et al., *Raf-like kinases CBC1 and CBC2 negatively regulate stomatal opening by negatively regulating plasma membrane H.* Photochem Photobiol Sci, 2020. **19**(1): p. 88-98.

24. Meinhard, M. and E. Grill, *Hydrogen peroxide is a regulator of ABI1, a protein phosphatase 2C from Arabidopsis.* FEBS Lett, 2001. **508**(3): p. 443-6.

25. Maheshwari, P., et al., *Model-driven discovery of calcium-related protein-phosphatase inhibition in plant guard cell signaling.* PLoS Comput Biol, 2019. **15**(10): p. e1007429.

26. Meinhard, M., P.L. Rodriguez, and E. Grill, *The sensitivity of ABI2 to hydrogen peroxide links the abscisic acid-response regulator to redox signalling.* Planta, 2002. **214**(5): p. 775-82.

27. Sridharamurthy, M., et al., *H2O2 inhibits ABA-signaling protein phosphatase HAB1.* PLoS One, 2014. **9**(12): p. e113643.

28. Sirichandra, C., et al., *Phosphorylation of the Arabidopsis AtrbohF NADPH oxidase by OST1 protein kinase.* FEBS Lett, 2009. **583**(18): p. 2982-6.

29. Guse, A.H., *Cyclic ADP-ribose: a novel Ca2+-mobilising second messenger.* Cell Signal, 1999. **11**(5): p. 309-16.

30. Sethi, J.K., R.M. Empson, and A. Galione, *Nicotinamide inhibits cyclic ADP-ribose-mediated calcium signalling in sea urchin eggs.* Biochem J, 1996. **319 ( Pt 2)**: p. 613-7.

31. Walseth, T.F. and H.C. Lee, *Synthesis and characterization of antagonists of cyclic-ADP-ribose-induced Ca2+ release.* Biochim Biophys Acta, 1993. **1178**(3): p. 235-42.

32. Rakovic, S., et al., *An antagonist of cADP-ribose inhibits arrhythmogenic oscillations of intracellular Ca2+ in heart cells.* J Biol Chem, 1999. **274**(25): p. 17820-7.

33. Joudoi, T., et al., *Nitrated cyclic GMP modulates guard cell signaling in Arabidopsis.* Plant Cell, 2013. **25**(2): p. 558-71.

34. Mulaudzi, T., et al., *Identification of a novel Arabidopsis thaliana nitric oxide-binding molecule with guanylate cyclase activity in vitro.* FEBS Lett, 2011. **585**(17): p. 2693-7.

35. Bright, J., et al., *ABA-induced NO generation and stomatal closure in Arabidopsis are dependent on H2O2 synthesis.* Plant J, 2006. **45**(1): p. 113-22.

36. Desikan, R., et al., *A new role for an old enzyme: nitrate reductase-mediated nitric oxide generation is required for abscisic acid-induced stomatal closure in Arabidopsis thaliana.* Proc Natl Acad Sci U S A, 2002. **99**(25): p. 16314-8.

37. Li, S., S.M. Assmann, and R. Albert, *Predicting essential components of signal transduction networks: a dynamic model of guard cell abscisic acid signaling.* PLoS Biol, 2006. **4**(10): p. e312.

38. Schulze, S., et al., *A Role for Calcium-Dependent Protein Kinases in Differential CO2- and ABA-Controlled Stomatal Closing and low CO2-induced Stomatal Opening in Arabidopsis.* New Phytol, 2021. **229**(5): p. 2765-2779.

39. Swatek, K.N., et al., *Multisite phosphorylation of 14-3-3 proteins by calcium-dependent protein kinases.* Biochem J, 2014. **459**(1): p. 15-25.

40. Sierla, M., et al., *The Receptor-like Pseudokinase GHR1 Is Required for Stomatal Closure.* Plant Cell, 2018. **30**(11): p. 2813-2837.

41. Merilo, E., et al., *PYR/RCAR receptors contribute to ozone-, reduced air humidity-, darkness-, and CO2-induced stomatal regulation.* Plant Physiol, 2013. **162**(3): p. 1652-68.

42. Geiger, D., et al., *Guard cell anion channel SLAC1 is regulated by CDPK protein kinases with distinct Ca2+ affinities.* Proc Natl Acad Sci U S A, 2010. **107**(17): p. 8023-8.

43. Brandt, B., et al., *Reconstitution of abscisic acid activation of SLAC1 anion channel by CPK6 and OST1 kinases and branched ABI1 PP2C phosphatase action.* Proc Natl Acad Sci U S A, 2012. **109**(26): p. 10593-8.

44. Scherzer, S., et al., *Multiple calcium-dependent kinases modulate ABA-activated guard cell anion channels.* Mol Plant, 2012. **5**(6): p. 1409-12.

45. Zhang, J., et al., *Identification of SLAC1 anion channel residues required for CO.* Proc Natl Acad Sci U S A, 2018. **115**(44): p. 11129-11137.

46. Meyer, S., et al., *AtALMT12 represents an R-type anion channel required for stomatal movement in Arabidopsis guard cells.* Plant J, 2010. **63**(6): p. 1054-62.

47. Jalakas, P., et al., *Combined action of guard cell plasma membrane rapid- and slow-type anion channels in stomatal regulation.* Plant Physiol, 2021. **187**(4): p. 2126-2133.

48. Imes, D., et al., *Open stomata 1 (OST1) kinase controls R-type anion channel QUAC1 in Arabidopsis guard cells.* Plant J, 2013. **74**(3): p. 372-82.

49. Sasaki, T., et al., *Closing plant stomata requires a homolog of an aluminum-activated malate transporter.* Plant Cell Physiol, 2010. **51**(3): p. 354-65.

50. Zhang, X., et al., *Inhibition of blue light-dependent H+ pumping by abscisic acid through hydrogen peroxide-induced dephosphorylation of the plasma membrane H+-ATPase in guard cell protoplasts.* Plant Physiol, 2004. **136**(4): p. 4150-8.

51. Kinoshita, T., M. Nishimura, and K. Shimazaki, *Cytosolic Concentration of Ca2+ Regulates the Plasma Membrane H+-ATPase in Guard Cells of Fava Bean.* Plant Cell, 1995. **7**(8): p. 1333-1342.

52. Negi, J., et al., *CO2 regulator SLAC1 and its homologues are essential for anion homeostasis in plant cells.* Nature, 2008. **452**(7186): p. 483-6.

53. Ward, J.M. and J.I. Schroeder, *Calcium-Activated K+ Channels and Calcium-Induced Calcium Release by Slow Vacuolar Ion Channels in Guard Cell Vacuoles Implicated in the Control of Stomatal Closure.* Plant Cell, 1994. **6**(5): p. 669-683.

54. Kohler, B., A. Hills, and M.R. Blatt, *Control of guard cell ion channels by hydrogen peroxide and abscisic acid indicates their action through alternate signaling pathways.* Plant Physiol, 2003. **131**(2): p. 385-8.

55. Sokolovski, S. and M.R. Blatt, *Nitric oxide block of outward-rectifying K+ channels indicates direct control by protein nitrosylation in guard cells.* Plant Physiol, 2004. **136**(4): p. 4275-84.

56. Grondin, A., et al., *Aquaporins Contribute to ABA-Triggered Stomatal Closure through OST1-Mediated Phosphorylation.* Plant Cell, 2015. **27**(7): p. 1945-54.
