# Supplementary material for: A network-based modeling framework reveals the core signal transduction network underlying high carbon dioxide-induced stomatal closure in guard cells
Source: PLoS Biol. 2024 May 1;22(5):e3002592. doi: 10.1371/journal.pbio.3002592 (PMC11090369; doi:10.1371/journal.pbio.3002592)
Supplement: S2 Text — (DOCX) [file pbio.3002592.s006.docx]

**S2 Text. Supporting Information**

**I. Description of ABA signaling elements whose participation in high CO_2_ signaling has not been tested**

Processes that have not been evaluated in CO_2_ signaling but whose role in ABA-induced closure is documented include vacuolar acidification, actin reorganization, and microtubule depolymerization (and the elements involved in the regulation of these processes). Indeed, knockout of the proton pumps that induce vacuolar acidification leads to slower or reduced ABA-induced stomatal closure [1], mutations that lead to impaired actin reorganization lead to reduced ABA-induced stomatal closure [2, 3], and conditions that inhibit microtubule depolymerization lead to reduced ABA-induced closure [4]. Furthermore, loss of the phospholipases PLC, PLDα and PLDδ, or of the products of the reactions they catalyze, leads to reduced closure in response to ABA [5-9]. The documented lack of cytosolic pH increase [10-12] in response to elevated CO_2_ serves as indirect evidence against the involvement of vacuolar acidification in high CO_2_ induced closure.

**II. Description of additional (conditionally) stable motifs of the CO_2_ network model and their effect on closure**

As shown in the main text analyses, the Main State Motif (MSM) includes the activity of RbohD/F and ROS, the minimum functional level of OST1, and the inactivity of the three PP2C protein phosphatases (ABI1, ABI2 and HAB1). Three overlapping conditionally stable motifs express the opposite states of the nodes involved in the MSM, namely the inactivity of RbohD/F, ROS, CaIM and GHR1, the lack of minimum functional level of OST1, and the activity of one of the PP2Cs. In addition to these decision-making motifs, the model contains several minor motifs, each of which arises from a node’s assumed self-sustaining activity. As described in S1 Text, these assumptions of self-sustained activity are needed to translate the oscillations in Ca^2+^_cyt_ into sustained closure.

The activity of the node CPKs forms a stable motif, indicating that after their activation by an increase in Ca^2+^_cyt_ the CPKs can sustain their activity. The inactivity of the node CPKs forms a conditionally stable motif, which depends on the inactivity (OFF state) of GHR1 simultaneously with a low level of Ca^2+^_cyt_. In settings in which Ca^2+^_cyt_ = GHR1 = 0 (for example, in the wild type system under ambient CO_2_), the OFF state of CPKs becomes a stable motif. Importantly, in these settings the ON state of CPKs is still also a stable motif. Depending on the initial state and trajectory of the system, either of these stable motifs can lock in, creating two attractors that only differ in the state of CPKs (see the third row of Figure 4D).

The ON state of the node “MPK activity” forms a conditionally stable motif conditioned on the presence of a sufficient amount of MPK proteins. We assume that a sufficient amount of MPK protein is present unless MPKs are knocked out (e.g. in the *mpk9/mpk12* double mutant studied in [13]). In such a setting an increase of Ca^2+^_cyt_ drives the MPK activity node into the ON state. The OFF state of MPK activity is a conditionally stable motif, which needs a sustained OFF state of Ca^2+^_cyt_.

The ON state of the node “K^+^ efflux” forms a conditionally stable motif that depends on the sustained activity of the Kout channel. The OFF state of the node is also a conditionally stable motif and depends on the sustained inactivity of the KEV channel.

The attractor that corresponds to high CO_2_ induced closure (first row of Figure 4D in main text) involves the lock-in of the MSM, the CPKs=ON stable motif, as well as the MPK activity=ON and K^+^ efflux=ON conditionally stable motifs. As shown in Figure 6D and explained in the Methods, the MSM leads to the activation of the CPKs=ON, MPK activity=ON, and K^+^ efflux=ON motifs.

Note that although the CPKs and MPK activity motifs participate in the process of high CO_2_ induced stomatal closure, they are not necessary for the process. We find that disruption of the CPKs and MPK activity motifs yields an attractor that is close to the wild type attractor and has Closure=ON. This is consistent with the observation of high CO_2_ induced stomatal closure in an MPK kinase dead mutant [14, 15] and in multiple *cpk* mutants [16]. On the other hand, the lock-in of the K^+^ efflux=ON motif is needed for sustained closure, otherwise the node Closure would have oscillations driven by Ca^2+^_cyt_.

The MPK activity=ON conditionally stable motif is notable in its ability to inhibit HT1 and thus drive the MSM. Thus, interventions under ambient CO_2_ that can activate even a transient increase in Ca^2+^_cyt_ lead to the lock-in of the MPK activity motif, and then to the lock-in of the MSM and an attractor with Closure=ON. This result is supported by the experimental observations of stomatal closure in response to providing ROS [17], cADPR [18], 8-nitro-cGMP [19], or external Ca^2+^ [20].

Lock-in of any of the three conditionally stable motifs shown in Figure 4B leads to sustained low Ca^2+^_cyt_, satisfying the condition of the CPKs=OFF, MPK activity = OFF, and K^+^ efflux=OFF motifs, and precluding the activation of the MPKs activity=ON and K^+^ efflux=ON motifs. The resulting attractor is shown in the second row of Fig 4D.

In conclusion, the decision between the lock-in of the Main Stable Motif and the three conditionally stable motifs that contradict it determines which of the minor conditionally stable motifs lock in, and drives the ultimate outcome (see Fig 4C).

**III. Experimental validation of the effect of NO on high CO_2_-induced stomatal closure by applying SNAP, an alternative NO donor**

In the main text, we presented the experimental validation of our model-predicted effects of NO on high CO_2_-induced stomatal closure using the NO donor SNP (sodium nitroprusside). Here we present an additional experiment using an alternative NO donor, SNAP (*S*-nitroso-*N*-acetylpenicillamine). We found that the effect of SNAP (Fig S1) is very similar to that of SNP. Under high (800 ppm) CO_2_, the average stomatal aperture after a 10 minute treatment with 150 μM SNAP (grey bars) is significantly lower than that without SNAP treatment (blue bars, p=1.8e-2, Student’s t-test). Furthermore, SNAP treatment can lead to stomatal closure in ambient CO_2_, as a 10 min treatment with 150 μM SNAP at 400 ppm CO_2_ led to a significant reduction of stomatal apertures (grey vs. blue in the left bar set, p=2.1e-2, Student’s t-test). These experimental findings confirm that the effect of SNAP is similar to that of SNP, and validate our model’s predicted effects of NO on high CO_2_-induced stomatal closure.


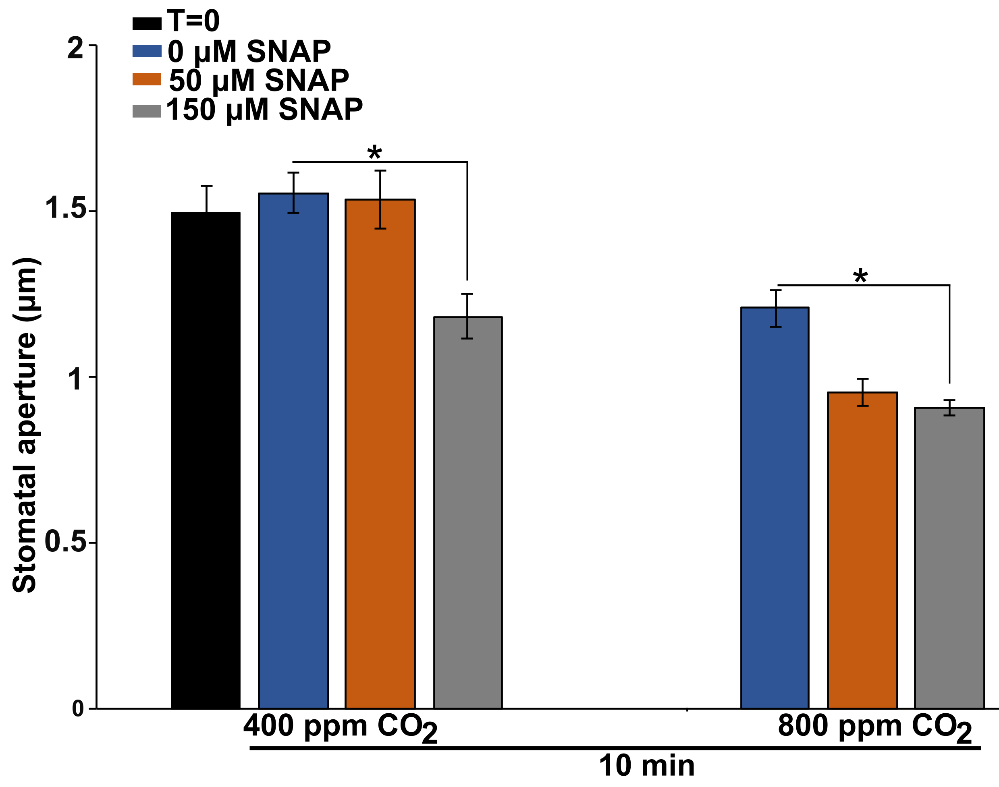


**Fig S1. Experimental validation of the effect of NO on high CO_2_-induced stomatal closure by applying SNAP, an alternative NO donor.** As predicted by our model, and similar to the effect of SNP, applying SNAP causes hypersensitivity in high CO_2_-induced stomatal closure: the combined treatment (orange and grey bars in the 800 ppm CO*_2_* condition) leads to a lower mean aperture than high CO_2_ alone (blue bars), and 10 min treatment of 150 μM SNAP at 400 ppm CO_2_ led to a significant reduction of aperture (grey vs. blue in the left bar set), showing SNAP treatment can lead to stomatal closure under ambient CO_2_. The bars indicate the mean ± SE aperture from at least three independent experiments with 30-40 apertures measured per treatment. The statistical significance of the aperture differences is determined by Student's t test: * P < 0.05. The data underlying the graphs are provided in S1 Data.

**IV. Description of how to add endogenous ABA and RCARs into the current model of high CO_2_-induced stomatal closure**

It is possible to add ABA and RCARs into our current model of high CO_2_-induced stomatal closure *if additional assumptions* are made. For example, based on Dittrich et al. [21], one can assume two additional nodes, " Endogenous ABA " (to represent an endogenous level of ABA) and “RCARs minimum function” (Fig S2). Endogenous ABA is always ON, unless specifically manipulated (such as in the *nced3;nced5* mutant [22]). Endogenous ABA activates the node “RCARs minimum function”, which is thus always ON, unless knocked out. Two additional assumptions are necessary for the model to capture high CO_2_-induced closure and the effect of RCAR knockout: (1) “RCARs minimum function” is insufficient to inactivate the PP2C phosphatases (ABI1, ABI2, HAB1), and (2) “RCARs minimum function” is necessary for the node “OST1 minimum function”. The first assumption is necessary because otherwise, endogenous ABA would cause stomata to be constitutively closed in the model, regardless of other conditions. The second assumption is necessary so that the model would yield impaired high CO_2_-induced closure in the case of RCAR knockout or in the *nced3;nced5* mutant. These assumptions could help our model capture the impairment of closure and also ROS production under high CO_2_ [22] observed in the *RCAR* mutants. We provide the relevant Boolean regulatory functions below.

Endogenous ABA * = ON (source node)

RCARs minimum function * = Endogenous ABA

OST1 minimum function * = RCARs minimum function and not ABI1 and not ABI2 and not HAB1 or not HT1


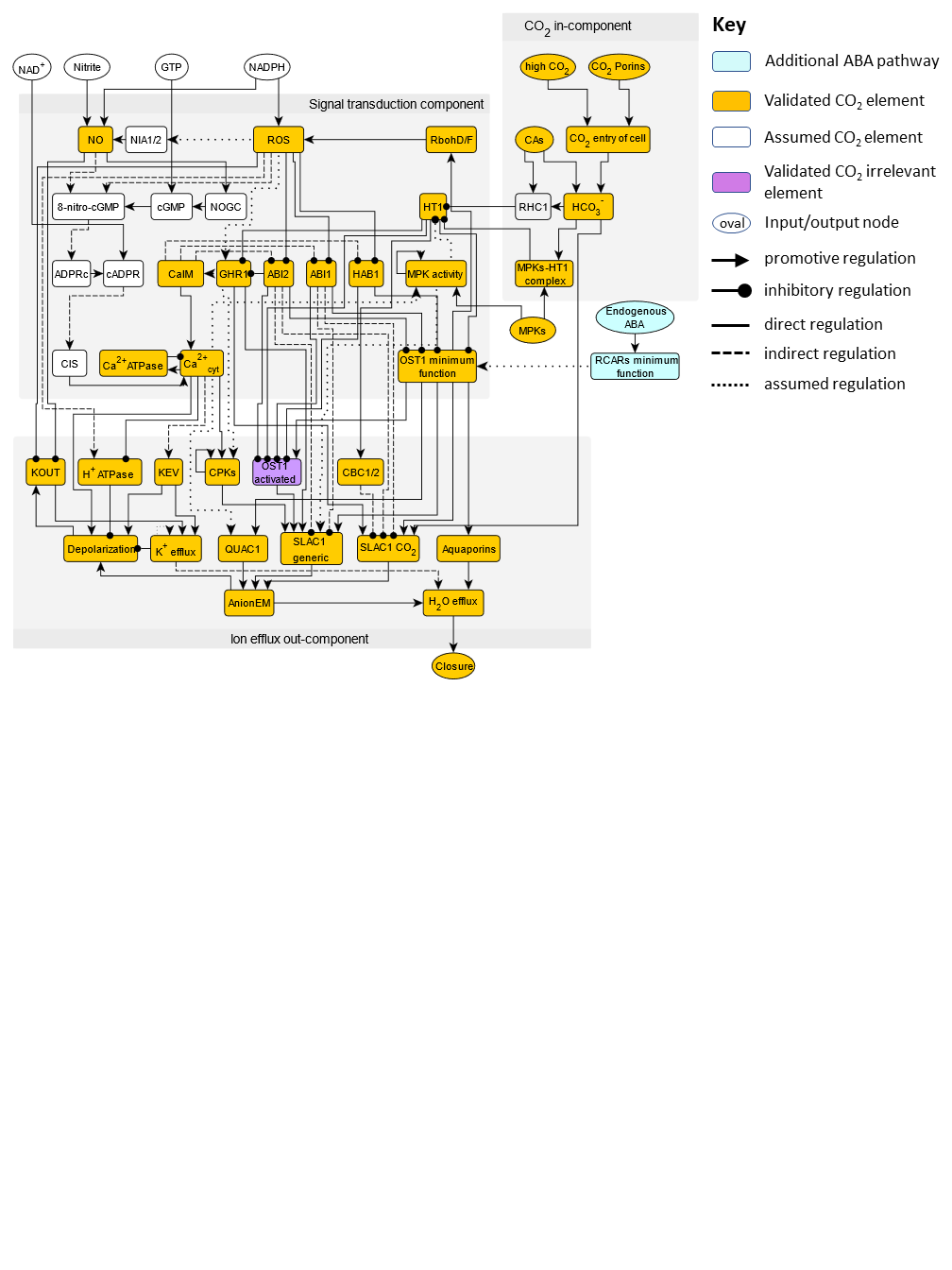


**Fig S2.** Adding endogenous ABA into the model of high CO_2_ induced stomatal closure. This node, when perceived by the PYL/PYR/RCARs receptors (represented by the “RCARs minimum function” node), forms a pathway necessary to activate OST1 minimum function. One also needs to assume that “RCARs minimum function” is insufficient to inactivate the PP2C phosphatases (ABI1, ABI2, HAB1), i.e. there are no edges from “RCARs minimum function” to any of the PP2C nodes. With these additional assumptions, the model would yield impaired high CO_2_-induced closure in the case of knockout of the RCAR receptors.

**References**

1. Bak, G., et al., *Rapid structural changes and acidification of guard cell vacuoles during stomatal closure require phosphatidylinositol 3,5-bisphosphate.* Plant Cell, 2013. **25**(6): p. 2202-16.

2. Zhao, Y., et al., *The plant-specific actin binding protein SCAB1 stabilizes actin filaments and regulates stomatal movement in Arabidopsis.* Plant Cell, 2011. **23**(6): p. 2314-30.

3. Jiang, K., et al., *The ARP2/3 complex mediates guard cell actin reorganization and stomatal movement in Arabidopsis.* Plant Cell, 2012. **24**(5): p. 2031-40.

4. Jiang, Y., et al., *Phosphatidic acid integrates calcium signaling and microtubule dynamics into regulating ABA-induced stomatal closure in Arabidopsis.* Planta, 2014. **239**(3): p. 565-75.

5. Mishra, G., et al., *A bifurcating pathway directs abscisic acid effects on stomatal closure and opening in Arabidopsis.* Science, 2006. **312**(5771): p. 264-6.

6. Staxen, I., et al., *Abscisic acid induces oscillations in guard-cell cytosolic free calcium that involve phosphoinositide-specific phospholipase C.* Proc Natl Acad Sci U S A, 1999. **96**(4): p. 1779-84.

7. Guo, L., et al., *Cytosolic glyceraldehyde-3-phosphate dehydrogenases interact with phospholipase Ddelta to transduce hydrogen peroxide signals in the Arabidopsis response to stress.* Plant Cell, 2012. **24**(5): p. 2200-12.

8. Meimoun, P., et al., *Intracellular Ca2+ stores could participate to abscisic acid-induced depolarization and stomatal closure in Arabidopsis thaliana.* Plant Signal Behav, 2009. **4**(9): p. 830-5.

9. Jacob, T., et al., *Abscisic acid signal transduction in guard cells is mediated by phospholipase D activity.* Proc Natl Acad Sci U S A, 1999. **96**(21): p. 12192-7.

10. Brearley, J., M.A. Venis, and M.R. Blatt, *The effect of elevated CO2 concentrations on K+ and anion channels of Vicia faba L. guard cells.* Planta, 1997. **203**(2): p. 145-154.

11. Savchenko, G., et al., *pH regulation in apoplastic and cytoplasmic cell compartments of leaves.* Planta, 2000. **211**(2): p. 246-255.

12. Xue, S., et al., *Central functions of bicarbonate in S-type anion channel activation and OST1 protein kinase in CO2 signal transduction in guard cell.* EMBO J, 2011. **30**(8): p. 1645-58.

13. Jammes, F., et al., *MAP kinases MPK9 and MPK12 are preferentially expressed in guard cells and positively regulate ROS-mediated ABA signaling.* Proc Natl Acad Sci U S A, 2009. **106**(48): p. 20520-5.

14. Takahashi, Y., et al., *Stomatal CO2/bicarbonate sensor consists of two interacting protein kinases, Raf-like HT1 and non-kinase-activity requiring MPK12/MPK4.* Science Advances, 2022. **8**(49): p. eabq6161.

15. Yeh, C.-Y., et al., *MPK12 in stomatal CO2 signaling: function beyond its kinase activity.* New Phytologist, 2023. **n/a**(n/a).

16. Schulze, S., et al., *A Role for Calcium-Dependent Protein Kinases in Differential CO2- and ABA-Controlled Stomatal Closing and low CO2-induced Stomatal Opening in Arabidopsis.* New Phytol, 2021. **229**(5): p. 2765-2779.

17. Kwak, J.M., et al., *NADPH oxidase AtrbohD and AtrbohF genes function in ROS-dependent ABA signaling in Arabidopsis.* EMBO J, 2003. **22**(11): p. 2623-33.

18. Leckie, C.P., et al., *Abscisic acid-induced stomatal closure mediated by cyclic ADP-ribose.* Proc Natl Acad Sci U S A, 1998. **95**(26): p. 15837-42.

19. Joudoi, T., et al., *Nitrated cyclic GMP modulates guard cell signaling in Arabidopsis.* Plant Cell, 2013. **25**(2): p. 558-71.

20. Hubbard, K.E., et al., *Abscisic acid and CO2 signalling via calcium sensitivity priming in guard cells, new CDPK mutant phenotypes and a method for improved resolution of stomatal stimulus-response analyses.* Ann Bot, 2012. **109**(1): p. 5-17.

21. Dittrich, M., et al., *The role of Arabidopsis ABA receptors from the PYR/PYL/RCAR family in stomatal acclimation and closure signal integration.* Nat Plants, 2019. **5**(9): p. 1002-1011.

22. Chater, C., et al., *Elevated CO2-Induced Responses in Stomata Require ABA and ABA Signaling.* Curr Biol, 2015. **25**(20): p. 2709-16.
